# Liver diseases: epidemiology, causes, trends and predictions

**DOI:** 10.1038/s41392-024-02072-z

**Published:** 2025-02-05

**Authors:** Can Gan, Yuan Yuan, Haiyuan Shen, Jinhang Gao, Xiangxin Kong, Zhaodi Che, Yangkun Guo, Hua Wang, Erdan Dong, Jia Xiao

**Affiliations:** 1 Department of Gastroenterology, West China Hospital, Sichuan University, Chengdu, China; 2 Aier Institute of Ophthalmology, Central South University, Changsha, China; 3 Department of Oncology, the First Affiliated Hospital; The Key Laboratory of Anti-inflammatory and Immune Medicine, Ministry of Education, Anhui Medical University, Hefei, China; 4 Engineering and Translational Medicine, Medical College, Tianjin University, Tianjin, China; 5 Clinical Medicine Research Institute and Department of Anesthesiology, The First Affiliated Hospital of Jinan University, Guangzhou, China; 6 Research Center for Cardiopulmonary Rehabilitation, University of Health and Rehabilitation Sciences Qingdao Hospital, School of Health and Life Sciences, University of Health and Rehabilitation Sciences, Qingdao, China; 7 Department of Cardiology and Institute of Vascular Medicine, Peking University Third Hospital, State Key Laboratory of Vascular Homeostasis and Remodeling, Peking University, Beijing, China; 8 Department of Gastroenterology, Qingdao Central Hospital, University of Health and Rehabilitation Sciences, Qingdao, China

**Keywords:** Gastrointestinal diseases, Gastrointestinal diseases

## Abstract

As a highly complex organ with digestive, endocrine, and immune-regulatory functions, the liver is pivotal in maintaining physiological homeostasis through its roles in metabolism, detoxification, and immune response. Various factors including viruses, alcohol, metabolites, toxins, and other pathogenic agents can compromise liver function, leading to acute or chronic injury that may progress to end-stage liver diseases. While sharing common features, liver diseases exhibit distinct pathophysiological, clinical, and therapeutic profiles. Currently, liver diseases contribute to approximately 2 million deaths globally each year, imposing significant economic and social burdens worldwide. However, there is no cure for many kinds of liver diseases, partly due to a lack of thorough understanding of the development of these liver diseases. Therefore, this review provides a comprehensive examination of the epidemiology and characteristics of liver diseases, covering a spectrum from acute and chronic conditions to end-stage manifestations. We also highlight the multifaceted mechanisms underlying the initiation and progression of liver diseases, spanning molecular and cellular levels to organ networks. Additionally, this review offers updates on innovative diagnostic techniques, current treatments, and potential therapeutic targets presently under clinical evaluation. Recent advances in understanding the pathogenesis of liver diseases hold critical implications and translational value for the development of novel therapeutic strategies.

## Introduction

The liver, a multifaceted organ, is central to regulating physiological processes including metabolism, detoxification, protein synthesis, and immune response.^1^ These functions are primarily mediated by hepatocytes, the major parenchymal cells within the liver. Supporting these are liver non-parenchymal cells (NPCs)— liver sinusoidal endothelial cells (LSECs), hepatic stellate cells (HSCs), cholangiocytes, Kupffer cells (KCs), and other immune cell types that maintain liver homeostasis.^2^ Liver sinusoids are lined by LSECs with characteristic fenestrations, which facilitate substantial exchanges between sinusoids and hepatocytes. HSCs reside in the space of Disse, secreting cytokines and growth factors that nurture neighboring cells. Cholangiocytes line the intra- and extrahepatic ducts of the biliary tree and contribute to the modification of hepatocyte-derived bile. KCs, along with other immune cells, play a pivotal role in defending against pathogens from the portal circulation.^2^

Liver diseases represent a wide array of disorders characterized by hepatocyte injury, inflammatory cell infiltration, and HSC activation, which cumulatively impair liver function and disrupt its architecture.^3^ Annually, liver diseases are linked to approximately 2 million deaths and account for 4% of global mortality.^4^ Acute liver diseases often result from hepatotropic virus infections, though drug-induced liver injury (DILI) is also becoming increasingly prevalent worldwide. Chronic liver conditions, on the other hand, typically arise from factors like alcohol consumption, hepatitis B virus (HBV), and hepatitis C virus (HCV) infections, along with a rising incidence of metabolic dysfunction-associated steatotic liver disease (MASLD) globally.^5^ Progression from such chronic conditions to end-stage liver diseases, including cirrhosis and liver cancer, contributes significantly to morbidity and mortality.^4^

Despite often presenting similar clinicopathological features—ranging from asymptomatic stages to nonspecific digestive symptoms—these liver diseases share biochemical and histological profiles that complicate their differentiation based on a single diagnostic parameter.^6^ Accurate diagnosis typically requires a combination of clinical presentation, specific biomarkers, and liver biopsy. Currently, clinical management of liver diseases largely focuses on hepatocyte protection, cause elimination, and symptom alleviation.^7^ The removal of causative agents such as ethanol and viruses does not always prevent progression to cirrhosis, suggesting that the underlying mechanisms driving disease onset and progression are incompletely understood.^8^ Thus, this review aims to provide an updated, comprehensive overview of the epidemiology and characteristics of liver diseases, highlight the complex pathogenetic mechanisms involved, and summarize the current clinical treatments and investigational drugs in clinical trials that hold potential for future therapeutic management.

## Epidemiology of liver diseases

### Global mortality

**Fig. 1:**
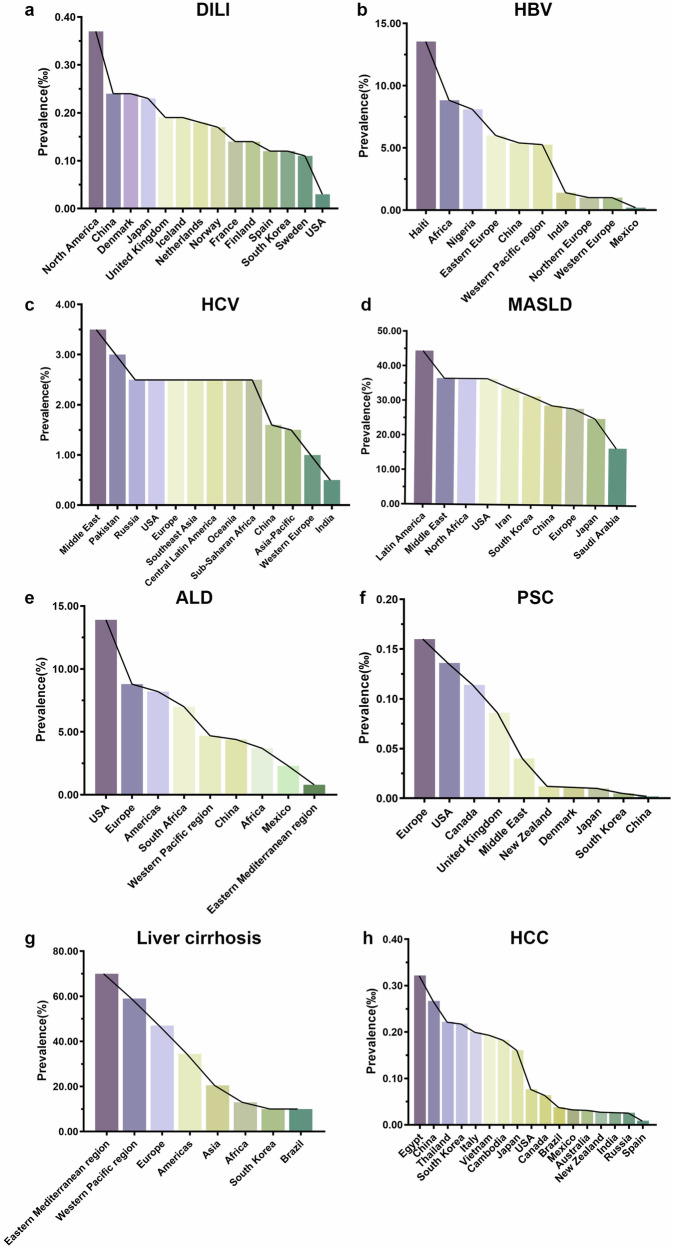
Worldwide distribution of liver disease prevalence. Prevalence of (**a**) drug-induced liver injury (DILI), (**b**) hepatitis B virus infection (HBV), (**c**) hepatitis C virus infection (HCV), (**d**) metabolic dysfunction-associated steatotic liver disease (MASLD), (**e**) alcohol-associated liver disease (ALD), (**f**) primary sclerosing cholangitis (PSC), (**g**) liver cirrhosis, and (**h**) hepatocellular carcinoma (HCC) are displayed

Liver disease stands as a leading cause of global mortality. The Global Burden of Disease 2019 study reported that 1.26 million individuals succumbed to cirrhosis and other chronic liver diseases in 2019, marking a 13% increase since 1990 (Fig. 1 and Table 1).^9^ Liver cancer, a terminal outcome of liver disease, accounted for approximately 830,000 deaths in 2020, representing 8.3% of global cancer-related deaths.^10^ Viral hepatitis, especially HBV and HCV, annually leads to around 1.3 million deaths.^11^ Moreover, approximately 3.3 million people are diagnosed with alcohol-associated liver disease (ALD) annually, accounting for 5.9% of global deaths.^12^ The rising fatalities from MASLD are also noteworthy, with an estimated 280,000 deaths in 2019.^13^ Notably, liver disease mortality rates show significant regional disparities; for example, Mongolia reports the highest liver cancer mortality rate at 71.0 per 100,000 individuals, compared to 6.6 in the United States (U.S.).^10^ This stark contrast arises primarily from the higher prevalence of HBV and HCV, limited healthcare resources, and elevated levels of alcohol consumption in Mongolia. Conversely, the U.S. benefits from effective hepatitis vaccination programs, comprehensive screening, and advanced treatment options, resulting in significantly lower mortality rates. While global trends indicate an increase in liver disease mortality, some high-income countries such as the U.S. have observed a decline since peaking in 2013, with an annual decrease of 3.2%.^14^ These variances underscore differences in disease burden, healthcare access, and public health strategies across different regions and countries.

**Table 1:** Global incidence and mortality of major liver diseases

Liver disease	Country/region	Incidence, per 100,000 person-years	Mortality, per 100,000 person-years	Years	References
HAV	Global	159,000,000 new infections (2019)^a^	39,000^a^	2019	^27^
	Sub-Saharan Africa	>90%	–	2018	^33^
	South Asia	>90%	–	2018	^33^
	Latin America	Intermediate	–	2010	^34^
	Middle East	Intermediate	–	2010	^34^
	North Africa	Intermediate	–	2010	^34^
	Eastern Europe	Intermediate	–	2010	^34^
	Asia	Intermediate	–	2010	^34^
	Western Europe	Low	–	2015	^551^
	Australia	Low	–	2023	^35^
	New Zealand	Low	–	2017	^552^
	Canada	Low	–	2021	^553^
	USA	Low	–	2016-2020	^554^
	Japan	Low	–	2021	^36^
HEV	Global	20,000,000 new infections (2017)^a^, 3,300,000 symptomatic	50,000–70,000^a^	2017	^28^
	South Asia	High	–	2016, 2021	^37,38^
	Africa	High		2016, 2021	^37,38^
	China	High		2016, 2021	^37,38^
	Latin America	High		2016, 2021	^37,38^
	Europe	Increasing (21.9–71.3%)	–	2015, 2018	^40,41,555^
DILI	Europe	2.3–19.1	–	2002, 2013,	^46,47^
	USA	2.7 (adults)	1.0 (idiosyncratic DILI causing ALF)	2017, 2019	^48,174^
	Asia	12.0–23.8	–	2012, 2019	^43,49^
HBV	Global	1,500,000 new infections (2019)^a^	820,000^a^	2019	^51^
	Western Pacific	526	–	2019, 2020	^54,56^
	Africa	883	–	2019, 2020	^54,56^
	Europe	<10 (Western & Northern), 40–80 (Eastern)	–	2018	^57^
	Americas	2 (Mexico), 135.5 (Haiti)	–	2015	^556^
HCV	Global	1,500,000 new infections (2022)^a^	–	2017, 2022	^26,62^
	Central Asia	>350	–	2013	^63^
	East Asia	>350	–	2013	^63^
	Southeast Asia	150–350	–	2013	^63^
	sub-Saharan Africa	150–350	–	2013	^63^
	Europe	150–350	–	2013	^63^
	Western Europe	<100	–	2013	^63^
	Asia-Pacific	<150	–	2013	^63^
	Tropical Latin America	<150	–	2013	^63^
	North America	<150	–	2013	^63^
HDV	North America	<10 (general population)	–	2019, 2020	^60,61^
	Northern Europe	<10 (general population)	–	2019, 2020	^60,61^
	Sub-Saharan Africa	>20	–	2019, 2020	^60,61^
	Central Asia	>20	–	2019, 2020	^60,61^
	Eastern Europe	>20	–	2019, 2020	^60,61^
ALD	Global	480	3,3	2010	^12^
	Europe	–	4.6 (females), 9.7 (males)	2010	^80^
	Mongolia	Highest burden	–	2019	^21^
	Kazakhstan	Highest burden	–	2019	^21^
	Guatemala	Highest burden	–	2019	^21^
	Greenland	Highest burden	–	2019	^21^
	Kyrgyzstan	Highest burden	–	2019	^21^
	Poland	Highest burden	–	2019	^21^
	Rwanda	Highest burden	–	2019	^21^
	Ireland	Highest burden	–	2019	^21^
	Brazil	Highest burden	–	2019	^21^
MASLD	Global	2530 (1990–2006), 3800 (2016–2019)	2.8	2019	^64^
	Latin America	4440	–	2016-2019	^64^
	Middle East and North Africa	3650	–	2016	^75^
	USA	1900 (1988–1994), 5400 (2005–2016)	–	1988-2016	^66^
PBC	Global	0.33–5.8	–	2013	^557^
	North America	218	–	1981-2020	^88^
	Europe	146	–	1971-2020	^88^
	Asia-Pacific	98	–	1991-2020	^88^
PSC	Global	0–1.3	–	2012	^91^
	Norway	162	–	2012	^91^
	Spain	6	–	2012	^89^
	USA	136 (Olmsted County)	–	2000	^558^
AIH	Global	1.4	–	2019	^92^
	Europe	19.4	–	2019	^92^
	Americas	22.8	–	2019	^92^
	Asia	13.0	–	2019	^92^
WD	Global	2.5–3.3	–	2012, 2017	^98,559^
	Europe	1.2–2	–	2012	^100^
	Asia	5.9 (China), 2.7 (South Korea), 1.9 (Japan)	–	2017	^98^
	Middle East	3.2 (Iran), 6.7 (Saudi Arabia)	–	2017	^560^
	U.S.	2.5–3.3	–	1999	^561^
AATD	Europe	25–50	–	2019	^102,562^
	North America	20.0–33.3	–	2019	^103,563^
	Asia	0.3 (Japan), 0.4 (South Korea)	–	2006-2007	^564^
	Africa	3.3 (African Americans)	–	2008	^565^
Cirrhosis	Global	20.7	110.6	2000	^5,9^
	Europe	–	150.00 (Western), 350.00 (Eastern)	2021	^8^
	North America	300–1000	–	2019	^103,563^
	Asia	165 (East Asia), 236 (Southeast Asia)	–	2019	^5^
	Africa	100–200	–	2020	^9^
	Latin America	250–300	–	2020	^9^
HCC	Global	15–30	830	2020	^10,111^
	Mongolia	856	–	2020	^10^
	China	267	–	2020	^111^
	South Korea	218	–	2020	^107^
	Japan	161	–	2021	^566^
	Egypt	322	–	2021	^112^
	Italy	109	–	2020	^111^
	Spain	86	–	2020	^567^

*AATD* alpha-1 antitrypsin deficiency, *AIH* autoimmune hepatitis, *ALD* alcohol-associated liver disease, *DILI* drug-induced liver injury, *HBV* hepatitis B virus infection, *HCC* hepatocellular carcinoma, *HCV* hepatitis C virus infection, *HDV* hepatitis D virus infection, *HEV* hepatitis E virus infection, *MASLD* metabolic dysfunction-associated steatotic liver disease, *PBC* primary biliary cholangitis, *PSC* primary sclerosing cholangitis, *WD* Wilson disease. ^a^If the total population was unavailable, the total number of cases is shown

### Global morbidity

Liver disease incidence is on the rise worldwide, posing an increasing risk of morbidity. Despite intensified global public health interventions, liver diseases continue to represent a significant portion of the global disease burden, underscoring the complexity and multidimensionality of liver disease epidemiology. The shift towards lifestyle-associated liver diseases, such as MASLD and ALD, is particularly alarming. This trend is closely linked to changes in global dietary habits, sedentary behavior, and rising obesity rates.^15,16^ Recent meta-analyses have identified MASLD as the most common chronic liver disease, affecting 38.0% of the global adult population between 2016-2019.^17^ Additionally, the incidence of ALD is climbing, paralleling increases in global alcohol consumption.^18^ This trend is evident in regional data that show significant correlations between increasing alcohol consumption and ALD rates. In the U.S., the age-adjusted death rate from ALD increased by 34.4% from 2009 to 2016, rising from 9.6 to 12.9 per 100,000 population, corresponding with a 7% increase in per capita alcohol consumption.^19^ Similarly, the United Kingdom (U.K.) experienced a 43% increase in hospital admissions for ALD between 2002 and 2019, accompanied by a 10% increase in alcohol sales.^20^ In South Korea, the age-standardized prevalence of ALD nearly doubled from 3.8% in 1998 to 7.4% in 2016, corresponding with a 43% increase in per capita alcohol consumption.^21^ Moreover, China has also seen a significant surge, the prevalence of ALD among patients with chronic liver diseases increased from 4.3% in 2000 to 8.7% in 2015, concurrent with a 70% increase in recorded alcohol consumption between 2005 and 2016.^22^ While new infections of HBV and HCV are declining in many regions due to effective public health interventions, chronic infections continue to pose a global challenge. The reduction in new infections can be attributed primarily to several key health measures beyond vaccination. Enhanced screening protocols for blood and organ donors have significantly reduced the risk of transfusion-associated hepatitis.^23^ Furthermore, harm reduction programs targeting high-risk populations, such as needle and syringe exchange programs, have been instrumental in preventing transmission among intravenous drug users.^24^ Moreover, the introduction of direct-acting antivirals (DAAs) for HCV has revolutionized treatment, achieving cure rates over 95% across all HCV genotypes.^25^ Improved infection control practices in healthcare settings have also minimized iatrogenic transmissions, underscoring the effectiveness of a comprehensive approach to combating these viral infections. The World Health Organization (WHO) estimates that there are 1.5 million new HBV and HCV infections annually.^26^ Acute hepatitis forms, such as hepatitis A (HAV) and hepatitis E (HEV), remain prevalent in developing countries, with approximately 10 million and 20 million new infections each year, respectively.^27,28^ Socioeconomic factors exacerbate disease progression, as evidenced by the rising global incidence of cirrhosis from 20.7 to 23.4 per 100,000 people between 2000 and 2015.^9^ The global incidence of cirrhosis, the end-stage of various chronic liver conditions, increased from 20.7 per 100,000 people in 2000 to 23.4 per 100,000 in 2015.^9^ Moreover, the incidence of liver cancer continues to escalate, with around 20 million new cases reported globally in 2022.^29^ Although improved diagnostics have enhanced early detection, they may also contribute to apparent increases in incidence. Emerging risk factors, such as environmental pollution and hepatotoxic drug use, further complicate efforts to reduce the global burden of liver diseases.^30,31^

### Acute liver disease

#### Acute viral hepatitis

HAV remains a significant global health concern despite strong vaccination recommendations, with an estimated 10 million new infections annually.^27^ In 2019, around 159 million acute HAV infections were reported worldwide.^32^ HAV prevalence varies considerably across different regions, with developing countries and low-income areas experiencing higher seroprevalence rates, particularly in sub-Saharan Africa and South Asia. In these regions, almost all children encounter HAV early in life, reducing susceptibility in adulthood.^33^ Conversely, in middle-income areas such as Latin America, the Middle East, North Africa, Eastern Europe, and parts of Asia, the prevalence exhibits transitional characteristics, posing a risk to unexposed adolescents and adults.^34^ High-income nations such as Western Europe, Australia, New Zealand, Canada, the U.S., Japan, and Singapore have very low HAV seroprevalence, with infections primarily confined to specific high-risk groups including travelers, men who have sex with men, drug users, the homeless, and the incarcerated.^35,36^

HEV leads to approximately 3.3 million symptomatic cases of acute hepatitis globally. The distribution of HEVs by genotype and geographic location is uneven and particularly prevalent in developing regions like South Asia, Africa, rural China, and Latin America. Here, genotypes 1 and 2 of HEV, which infect only humans and are transmitted through contaminated water sources, precipitate major outbreaks in resource-limited settings.^37,38^ In recent decades, there has been a significant increase in HEV cases in Europe, with seropositivity rates ranging from 20% to 30% in countries including France, Germany, and the Netherlands.^39–41^ While the overall mortality from HEV is low, specific high-risk groups, such as immunocompromised individuals, face greater health threats and higher case fatality rates.^42^ Targeted monitoring and protective measures for these vulnerable groups remain crucial.

#### Drug-induced liver injury

Determining the precise incidence of DILI is challenging due to diagnostic complexities and widespread underreporting. The estimates of DILI incidence fluctuate significantly, from 1 in 10,000 to 1 in 1,000,000 cases, influenced by variable factors such as diagnostic criteria, detection capabilities, population demographics, drug types, cultural factors, and reporting practices.^42,43^

In Europe, retrospective studies from the U.K. and Sweden suggest an annual incidence of DILI around 2.3-2.4 per 100,000 individuals.^44,45^ France reported a higher figure from a prospective study, at 13.9 cases per 100,000, equating to over 8,000 cases annually.^46^ In Iceland, prospective data revealed a rate of 19.1 cases per 100,000 people.^47^ In the U.S., a Delaware-based study noted an annual incidence of 2.7 cases per 100,000 adults, with nearly 43% linked to herbal and dietary supplements.^48^ A comparative analysis shows that DILI incidence is generally higher in Asian countries; a nationwide prospective study in South Korea documented an annual rate of 12 hospitalizations per 100,000 due to DILI,^49^ while a retrospective study in China reported 23.8 cases per 100,000.^43^ Notably, the proportion of DILI cases attributed to herbal and dietary supplements is on the rise globally, indicating an evolving trend in the epidemiology of DILI and pointing towards the need for enhanced awareness and regulation.

### Chronic liver diseases

#### Chronic hepatitis B/D

Chronic hepatitis B (CHB) remains a significant public health challenge globally. As per the 2019 WHO data, the worldwide seroprevalence of hepatitis B surface antigen (HBsAg) is 3.8%, accounting for approximately 296 million people living with CHB.^50^ The GBD study from the same year estimated CHB prevalence at around 4.1%, translating to approximately 316 million cases globally.^51^ Over the three decades leading up to 2019, CHB prevalence saw a notable decrease of 31.3%.^21^ This notable decline is primarily due to the global implementation of universal HBV vaccination programs, which have significantly reduced new infections among newborns and children, the most vulnerable to chronic infection.^52^ Additionally, targeted public health initiatives, including enhanced maternal and perinatal healthcare services, have effectively prevented vertical transmission from mothers to infants. Increased public awareness through education campaigns, along with improved access to healthcare, has further contributed to the reduction in CHB prevalence.^53^ The highest CHB prevalence rates occur in the Western Pacific region (5.26%) and Africa (8.83%), with the disease burden in West and Central Africa accounting for 82.8% and 17.2%, respectively.^54,55^ The countries most afflicted by CHB are China, India, and Nigeria, harboring 74 million, 17 million, and 15 million cases respectively.^56^ Europe exhibits significant intra-regional variation in CHB prevalence, with less than 1% in Western and Northern Europe, contrasting sharply with 4-8% in Eastern Europe. Prevalence rates in the Americas are also diversified, with Mexico at 0.20% and Haiti at 13.55%.^57^

Among specific populations, approximately 2.7 million people living with human immunodeficiency virus (HIV) are co-infected with HBV, 71% of whom are in sub-Saharan Africa. Furthermore, around 0.5% (1.3 million) of injection drug users globally are HBV-positive.^58^ Hepatitis delta (HDV) co-infection has been significantly impacted by vaccination efforts^59^; although an estimated 15-20 million people worldwide are infected with HDV, the exact prevalence rates are elusive due to a dearth of comprehensive studies. The most affected countries include Benin, Gabon, Mauritania, Nauru, and Mongolia, with the latter exhibiting the highest rate at 36.9% of HBsAg-positive individuals co-infected with HDV. Despite a global decline in HDV due to vaccination initiatives, persistent high prevalence in regions like Moldova and parts of Africa and Asia underscore an ongoing health burden.^60,61^

#### Chronic hepatitis C

In 2020, approximately 57 million people were estimated to be living with chronic HCV infection globally, exhibiting a viremia prevalence rate of 0.7%. Over 70% of these cases were concentrated in low- and middle-income countries. The highest burdens of HCV are seen in China, India, Pakistan, Russia, and the U.S., with 30 countries collectively accounting for 80% of the global HCV burden.^62^

High HCV prevalence regions include Central Asia, East Asia, and the North Africa-Middle East, each with over 3.5% of the population affected. Moderate prevalence rates (1.5-3.5%) are noted in Southeast Asia, the Andes, Central and South Latin America, Oceania, the Caribbean, Europe, and sub-Saharan Africa. Contrastingly, some Western European nations like the U.K., Denmark, France, Germany, Sweden, and Switzerland report HCV seroprevalence rates of less than 1%. The Asia-Pacific region, tropical Latin America, and North America each have relatively low prevalence rates, under 1.5%.^63^ Despite a decreasing global trend in HCV prevalence, there is a critical need for focused interventions and increased attention in high-burden countries and regions.

#### Chronic metabolic liver diseases—MASLD and ALD

MASLD has rapidly become the most prevalent chronic liver disease worldwide. Its global prevalence escalated from 25.3% between 1990-2006 to 38.0% between 2016-2019, heavily influenced by the rising rates of obesity and type 2 diabetes mellitus (T2DM).^64^ MASLD exhibits substantial regional variations; in the Americas, Latin America reports the highest prevalence at 44.4%. In the U.S., the prevalence surged from 19% in 1988-1994 to 54% in 2005-2016.^65,66^ In Europe, there is variability in the prevalence of MASLD. A recent meta-analysis, which includes data updated until 2019, indicates an increase in prevalence to 30.9%.^13^ Western European nations like Germany and the U.K. displaying higher rates (25%-30%), whereas Eastern countries such as Hungary and Romania have slightly lower prevalence (around 20%).^67–69^ The trend is similarly upward in the Asia-Pacific region: China saw an increase from 25.4% in 2008-2010 to 32.3% in 2015-2018,^70,71^ and the latest nationwide study with 5.7 million showed that the prevalence of steatosis reached 44.39% in 2022.^72^ Japan experienced a rise from 20.69% in 1983 to 29.61% in 2011-2016;^73^ and South Korea reports a prevalence of approximately 31.5%.^74^ In Africa and the Middle East, data on MASLD incidence and prevalence in sub-Saharan Africa are largely missing, but this burden is expected to grow in the coming decades.^75^ Although data are sparse for Africa and the Middle East, the prevalence in sub-Saharan Africa is anticipated to climb in the coming decades. In the Middle East and North Africa region, high obesity and diabetes rates have significantly driven up MASLD prevalence, currently estimated at 36.5%.^75,76^

Notably, lean MASLD, a subtype of MASLD characterized by a BMI < 25 kg/m², shows a significantly higher prevalence in China (approximately 20%) compared to 5-10% in Western nations.^77,78^ This disparity is influenced by factors such as genetic variations (e.g., *PNPLA3* gene variants), differences in body composition (higher percentage of body fat and visceral adipose tissue at lower BMI in Asians), dietary habits, environmental factors, and gut microbiome variations.^79^ Despite normal BMI, lean MASLD patients in Asian populations face similar metabolic risks and liver disease progression as their obese counterparts. The high prevalence of lean MASLD underscores the need for tailored diagnostic and screening approaches in Asian countries like China to prevent underdiagnosis, considering unique regional and ethnic characteristics.^79^

ALD remains a major global health concern. Roughly 2.4 billion people consume alcohol worldwide, contributing to about 2 million deaths from liver disease annually, half of which are related to cirrhosis from alcohol consumption. ALD ranks among the top 30 causes of death globally, with a death rate from alcohol-attributable cirrhosis of 7.2 per 100,000 people in 2010 (4.6 in females and 9.7 in males).^80^ The relationship between liver-related death rates and alcohol consumption levels varies by country. European countries have historically been the largest per capita consumers of alcohol, though consumption decreased from 12.3 to 9.8 liters annually between 2005 and 2016. Conversely, alcohol consumption has been increasing in the Western Pacific, South-East Asia, and the Americas, with future growth projected until at least 2025.^81^ In China, alcohol consumption has increased more rapidly over the past 30 years than in any other country.^82^ Countries like the U.S. are also reporting rising rates of harmful drinking. In 2019, nations severely affected by ALD included Mongolia, Kazakhstan, El Salvador, Guatemala, Greenland, Kyrgyzstan, Poland, Rwanda, Ireland, and Brazil, all of which exhibit high alcohol consumption correlating with elevated ALD incidence and mortality rates.^21^

During the COVID-19 pandemic, social isolation and psychological stress significantly boosted alcohol consumption in certain populations in the U.S. and Europe, potentially exacerbating ALD prevalence.^83–85^ Despite intensified public health efforts, MASLD and ALD continue to pose significant challenges due to their complex etiology involving genetic predisposition, environmental factors, and lifestyle choices such as diet and alcohol use.^16,86^ These diseases often progress asymptomatically, complicating early detection and treatment. The COVID-19 pandemic has further exacerbated these challenges, increasing risk factors linked to lifestyle changes and stress.^87^ Consequently, there is a pressing need for adaptive public health strategies that go beyond traditional interventions. This includes not only developing targeted screening and comprehensive lifestyle interventions but also exploring innovative therapeutic options and addressing social determinants of health to effectively reduce the global disease burden.

#### Autoimmune liver diseases

Autoimmune liver diseases, including primary biliary cholangitis (PBC), primary sclerosing cholangitis (PSC), and autoimmune hepatitis (AIH), form a significant part of the global spectrum of liver diseases, displaying notable regional and demographic variations in prevalence.

PBC predominantly affects middle-aged and older women, with a global prevalence around 14.6 per 100,000. The highest prevalence rates are seen in North America (approximately 21.8 per 100,000), Europe (14.6 per 100,000), and the Asia-Pacific region (9.8 per 100,000). Within continents, discrepancies exist; for instance, in Europe, the prevalence varies from 13.8 per 100,000 in Northern Europe to 10.3 per 100,000 in Western Europe, with scant data from Southern and Eastern Europe. In the Asia-Pacific region, Japan and South Korea report higher prevalence rates (10.4 and 8.5 per 100,000, respectively) compared to mainland China (5.8 per 100,000) and Taiwan district (3.7 per 100,000).^88^

PSC primarily affects young adult males (male-to-female ratio approximately 2:1) and shows significant regional prevalence distinctions. For instance, Northern European countries like Sweden report a high prevalence of 10.3 per 100,000, considerably greater than Southern nations like Spain (0.6 per 100,000).^89^ The U.K. saw a rising prevalence from 3.2 per 100,000 in 1998 to 7.4 per 100,000 in 2014.^90^ In North America, PSC prevalence (e.g., 13.6 per 100,000 in Olmsted County, Minnesota, U.S.) typically surpasses that in Europe. Conversely, Asian countries generally report lower rates, with Japan, South Korea, and Singapore exhibiting prevalence of 0.95, 0.45, and 0.15 per 100,000, respectively.^89^ Notably, around 65% of PSC patients also have inflammatory bowel disease (IBD), particularly ulcerative colitis, underscoring a significant association between PSC and intestinal immune disorders.^91^

AIH has a global prevalence of 17.44 per 100,000, with regional prevalence rates at 12.99 in Asia, 19.44 in Europe, and 22.80 in the Americas per 100,000 respectively.^92^ AIH incidence in Denmark rose from 1.37 per 100,000 in 1994 to 2.33 per 100,000 in 2014.^93^ In the U.K., the incidence doubled from 1.27 in 1997 to 2.56 per 100,000 in 2015, with higher latitudes correlating with increased incidence.^94^ Sweden saw an increase from 10.7 per 100,000 in 2003 to 17.3 per 100,000 in 2009.^95^ Japan reported a substantial increase in AIH prevalence from 8.1 per 100,000 in 2004 to 23.9 per 100,000 in 2016.^96^ While the incidence has remained relatively stable in South Korea, there has been a gradual increase in prevalence from 2009 to 2013.^97^ The precise etiology of AIH remains elusive, though there is speculation that environmental changes may act as triggers.^88^ These diseases exemplify the complex interplay of genetic, environmental, and immunological factors that characterize autoimmune pathologies affecting the liver.

#### Genetic and rare liver diseases

Wilson disease (WD), an autosomal recessive disorder affecting copper metabolism, leads to significant liver and neurological damage. The estimated global prevalence of WD lies between 1:30,000 and 1:40,000 but shows notable ethnic and regional variability. In Europe, WD prevalence spans from approximately 1.2 to 2.0 per 100,000. It is comparatively higher in Asia, with China reporting a rate of 5.87 per 100,000, South Korea at 2.7 per 100,000, and Japan at 1.9 per 100,000.^98^ A U.K. study highlighted a potentially higher-than-expected risk, suggesting a carrier rate for pathogenic mutations in WD at 1/7,026-indicative of an underestimation of WD prevalence within the general population.^99^ The Middle East also exhibits significant prevalence, with Iran reporting 1/31,000 and Saudi Arabia at 1/15,000. In the U.S., the prevalence is around 1:30,000 to 1:40,000, but specific subpopulations like Armenians in New York City show a higher prevalence of 1/22,000. In Brazil, the prevalence aligns closely with the global average at about 1:36,000. It is notably higher in isolated regions with high rates of consanguineous marriages, such as the Canary Islands and Sardinia, with prevalence of 1/2,600 and 1/7,000, respectively.^99,100^ The frequently underestimated prevalence of WD may be attributed to misdiagnosis, phenotypic diversity, inadequate sensitivity of copper metabolism tests, and low detection rates of *ATP7B* gene mutations.^101^

Alpha-1 antitrypsin deficiency (AATD) predominantly affects the lungs and liver and is another prevalent genetic condition with distinct genetic variations influencing its distribution. The most prevalent AATD genotype among people of European descent is Pi*ZZ, occurring at a rate of about 1/2,000 to 1/4,000.^102^ North America reports a Pi*ZZ prevalence ranging from 1/3,000 to 1/5,000, with specific populations like Newfoundland experiencing higher rates, around 1/1,100.^103^ Conversely, Pi*ZZ prevalence is markedly lower in Asia and Africa: Japan reports a prevalence of 1/300,000, South Korea at 1/280,000, and African Americans at 1/30,000. Notably, the prevalence of AATD in Caucasian populations is generally higher compared to other genetic liver diseases, such as AIH, PSC, and WD.^104^

### End-stage liver diseases

#### Cirrhosis

Cirrhosis, representing the end stage of diverse chronic liver disorders, has experienced a global surge in incidence, from 20.7 per 100,000 people in 2000 to 23.4 per 100,000 in 2015-a 13% increase.^9^ The leading causes of cirrhosis include MASLD (60%), HBV (29%), HCV (9%), and ALD (2%).^105^

Regionally, HBV is most prevalent among cirrhosis patients in the Western Pacific (59%) and least prevalent in the Americas (5%). The highest proportion of cirrhosis due to HCV occurs in the Eastern Mediterranean (70%), while it is lowest in Africa and the Western Pacific (13% each). In terms of alcohol-related cirrhosis, Europe (16–78%) and the Americas (17–52%) report higher rates compared to Asia (0-41%). Data on MASLD as a cause of cirrhosis show that its prevalence varies, from 2% in South Korea and Brazil to 18% in Canada.^8^

In North America and Europe, MASLD is increasingly acknowledged as a primary cause of cirrhosis. For example, the prevalence of cirrhosis in the U.S. has increased between 1.5 to 2 fold over the past two decades, especially among younger populations due to prevalent obesity, diabetes, and metabolic syndrome.^103^ In Germany, MASLD-related cirrhosis cases saw a fourfold increase from 2005 to 2018.^106^ Similarly, in Japan, cirrhosis due to MASLD rose from 2% in 2007 to 9% in 2016^107^; South Korea also reports rising cirrhosis cases caused by MASLD, HCV, and alcohol.^108^ Despite these trends, viral hepatitis remains the dominant cause of cirrhosis in the Middle East and Africa, particularly HCV.^9,109^ Overall, NAFLD and ALD-related cirrhosis are becoming more common globally, although HBV and HCV infections remain the primary causes in many developing countries.

#### Hepatobiliary cancer

Hepatocellular carcinoma (HCC) is the most prevalent primary liver cancer globally and often develops within the context of chronic liver diseases such as cirrhosis. In 2020, the global prevalence of HCC ranged from approximately 15-30 per 100,000 with over 70% of cases in Asia.^110^ Mongolia reports the highest prevalence (85.6 per 100,000) in East Asia, followed by China (26.7 per 100,000), South Korea (21.8 per 100,000), and Japan (16.1 per 100,000).^10,111^ Southeast Asia also experiences a high HCC prevalence, with numbers like Thailand (22.2 per 100,000), Vietnam (19.4 per 100,000), and Cambodia (18.3 per 100,000). In Africa, the HCC prevalence ranges from 10-20 per 100,000, with Egypt reporting the highest at 32.2 per 100,000.^112^ Southern European nations like Italy (10.9 per 100,000) and Spain (8.6 per 100,000) report higher HCC rates compared to other European regions. In North America, HCC prevalence stands at about 6-8 per 100,000, while in Latin America, rates are slightly lower, ranging from 3-5 per 100,000, with Brazil and Mexico recording the highest numbers within this range. Oceania reports significantly lower HCC prevalence, as seen in Australia (3.2 per 100,000) and New Zealand (2.8 per 100,000).^113,114^

The global heterogeneity in HCC prevalence reflects the complex interplay of diverse etiological factors. In East and Southeast Asia, chronic HBV infection remains a major contributor, historically exacerbated by vaccination gaps and vertical transmission.^111,115^ Conversely, Western countries typically exhibit lower HCC rates due to effective HBV vaccination programs and improved antiviral therapy access.^114,116^ In Africa, particularly Egypt, high HCV prevalence is linked to elevated HCC rates, with limited healthcare access and socioeconomic challenges playing significant roles.^117^ In Southern Europe, cultural practices such as traditional alcohol consumption patterns also contribute to increased HCC risk.^118^ Moreover, the global rise in obesity and associated metabolic dysfunction represents an emerging HCC risk factor, particularly in Western regions.^119^ Environmental factors, including aflatoxin exposure in specific African and Asian regions, further modulate HCC risk.^120^ These multifaceted influences underscore the urgent need for region-specific prevention and screening strategies to effectively mitigate the global HCC burden.

Cholangiocarcinoma (CCA), though less common than HCC, has exhibited an increasing trend in the U.K. and the U.S., particularly with intrahepatic forms, while extrahepatic CCA has seen a decline.^121,122^ The U.S. recorded an increase in intrahepatic CCA incidence from 0.44 per 100,000 in 1973 to 1.18 per 100,000 in 2012.^123^ This rise is largely attributed to the increasing prevalence of obesity and metabolic syndrome in Western countries.^124^ In contrast, CCA incidence in Southeast Asian countries like Thailand is much higher than HCC, reaching 14.6 per 100,000.^125^ Factors such as liver fluke infections prevalent in Southeast Asia significantly contribute to the high regional CCA incidence. Notably, while CCA primarily affects middle-aged and older men, the rising incidence among women and younger individuals calls for further research into its evolving epidemiology.^126^

## Clinical and pathological features of liver diseases

### Clinical features

**Fig. 2:**
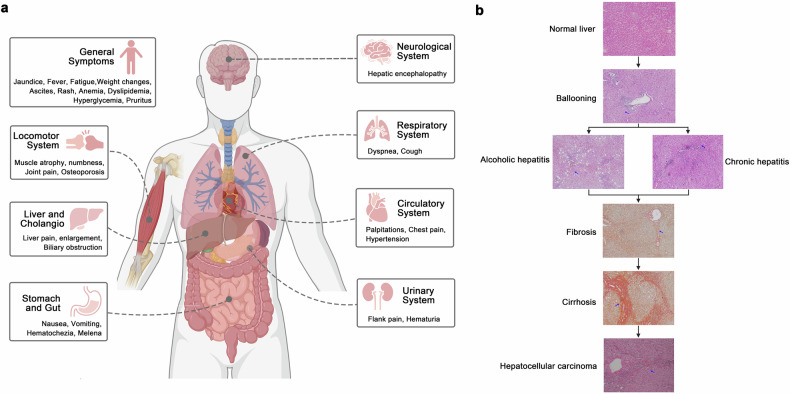
**a** Hepatic and extrahepatic manifestations associated with various liver diseases. **b** Histological progression from normal liver to hepatocellular carcinoma (HCC). Representative hematoxylin and eosin (H&E) stained sections illustrate the stages from normal liver to ballooning degeneration, alcoholic hepatitis, chronic hepatitis, and HCC. Sirius Red staining was used to visualize fibrosis and cirrhosis. Written informed consent was obtained from all patients involved. The study was approved by the Ethical Committee of West China Hospital and registered in the Chinese Clinical Trial Registry (ChiCTR2200063108). Created in BioRender. Yuan, Y. (2024) BioRender.com/o74p618

Liver diseases manifest a broad spectrum of symptoms, ranging from early nonspecific signs to advanced multisystem complications (Fig. 2A). Typically, at their onset, conditions such as acute viral hepatitis, mild DILI, early-stage chronic viral hepatitis, MASLD, and initial-phase ALD manifest with mild, nonspecific symptoms.^127,128^ Patients may experience minor fatigue, upper right abdominal discomfort, and a decreased appetite. Those with acute viral hepatitis might exhibit transient fever, nausea, and slight jaundice. MASLD patients often present with features of metabolic syndrome such as obesity, dyslipidemia, and hypertension.^129,130^ Early ALD may manifest as indigestion and abdominal discomfort. Mild DILI might lead to slight elevations in transaminases without prominent symptoms.^131^ Notably, many patients with early-stage liver disease are asymptomatic, being discovered incidentally during routine examinations or investigations for other reasons.

As liver diseases progress to the intermediate stage, symptoms previously mild or intermittent become more pronounced across various conditions. Patients with chronic viral hepatitis often experience ongoing fatigue, intermittent jaundice, and increasingly severe right upper quadrant pain, often accompanied by general malaise and mild hepatomegaly.^35^ In cases of chronic HCV, approximately 70% of patients may develop systemic complications such as mixed cryoglobulinemia and cardiovascular issues, underscoring the extensive impact of the disease.^132^ Concurrently, immune-mediated liver diseases demonstrate their unique progression patterns during this stage. AIH frequently manifests with nonspecific symptoms like fatigue in 85-95% of patients, often accompanied by symptoms like jaundice (67–85%) and abdominal pain (50–70%). Furthermore, 25–40% of patients display extrahepatic manifestations such as arthralgia and skin rashes, and up to 50% may suffer from concurrent autoimmune disorders such as thyroiditis.^133^ PBC exhibits persistent pruritus and fatigue in 65–85% of patients, with 50–60% initially asymptomatic, typically identified during routine liver function tests.^134^ It is often associated with other autoimmune conditions such as Sjögren’s syndrome (25%) and thyroid disorders (20%).^135^ PSC often exhibits pruritus and jaundice; typical symptoms include fatigue (75–80%) and right upper quadrant pain (20–40%), strongly linked to IBD, especially ulcerative colitis in 60–80% of cases, and an elevated lifetime risk of cholangiocarcinoma (10–15%) and colorectal cancer.^136–138^ In MASLD and ALD, signs of liver function abnormalities and mild coagulation disorders emerge, indicating progressive liver impairment.^139,140^

In the severe stages of liver diseases, patients often develop more serious and complex complications. Individuals with advanced chronic liver disease and compensated cirrhosis typically exhibit signs of portal hypertension, such as ascites, splenomegaly, and palmar erythema. These signs reflect significant changes in liver structure and function and alterations in portal systemic hemodynamics. Patients with WD often begin to exhibit significant neuropsychiatric symptoms, including movement disorders and cognitive decline at this stage, likely due to disturbances in copper metabolism affecting the central nervous system.^141^ Patients with AATD might concurrently suffer from respiratory symptoms such as difficulty breathing.^142^ Additionally, patients with late-stage liver disease commonly develop coagulopathies and thrombocytopenia, increasing the risk of bleeding. Early hepatic encephalopathy typically presents as mild cognitive impairment and disturbances in the sleep-wake cycle, early indicators of severe liver function compromise affecting the neurological system.^143^

The end stages of liver disease, including decompensated cirrhosis and hepatobiliary malignancies, are characterized by life-threatening complications. Patients often experience esophageal and gastric variceal bleeding, refractory ascites, and severe hepatic encephalopathy. These manifestations represent the terminal expressions of hepatic synthetic failure, altered hemodynamics, and cerebral dysfunction resulting from liver failure. Patients with HCC typically exhibit weight loss, abdominal masses, and cachexia; meanwhile, CCA patients might present as painless progressive jaundice and abdominal pain. Additionally, patients with end-stage liver disease often develop multisystem complications, including systemic coagulopathy, hepatorenal syndrome, and hepatopulmonary syndrome.^144,145^

In summary, the progression of liver disease constitutes a complex, multistage process that involves multiple systems and organs. Ranging from mild early symptoms to life-threatening complications in the end stages, the clinical manifestations at each stage illustrate the extent of liver damage and its systemic impact. Significantly, various types of liver diseases can progress at differing speeds and exhibit distinct clinical manifestations. This understanding is crucial for early detection of liver diseases, the assessment of disease severity, and accurate prognostication.

### Pathological features

Liver histology, crucial for diagnosing various diseases, plays a pivotal role in understanding and recognizing hepatic conditions (Fig. 2B).

In the initial stages of liver disease, pathological alterations are generally mild. Acute viral hepatitis typically features mononuclear cell and lymphocyte infiltration in hepatic lobules and portal areas.^146^ DILI may be indicated by slight hepatocellular swelling and minimal inflammatory cell infiltration.^147^ MASLD and ALD initially present with steatosis without significant inflammation. These changes are typically reversible, with timely intervention preventing disease progression.^19^

As the disease progresses to moderate stages, pathological changes become increasingly pronounced. Chronic viral hepatitis is characterized by persistent inflammation, interface hepatitis, and varying degrees of fibrosis. In advanced MASLD and ALD, worsening steatosis is accompanied by significant inflammatory cell infiltration and hepatocyte ballooning.^148^ PBC is distinguished by chronic non-suppurative inflammation and interlobular bile duct destruction, while PSC presents with concentric periductal fibrosis, the classic “onion-skin” appearance.^149,150^ AIH is characterized by interface hepatitis with prominent plasma cell infiltration. These pathological changes reflect disease progression and potentially indicate more severe liver dysfunction.

In the severe stage of liver disease, pathological alterations become more pronounced. Advanced chronic liver disease presents with significant bridging fibrosis and early nodule formation. WD is characterized by hepatocellular copper accumulation and the presence of Mallory bodies.^151^ AATD manifests as PAS-positive, diastase-resistant globules in periportal hepatocytes. Early cirrhosis, characterized by fibrous septa formation and lobular architecture distortion, begins to manifest. These changes reflect severe liver structural and functional impairment, often indicating irreversible liver damage.^152,153^

End-stage liver disease presents the most dramatic pathological features. Liver cirrhosis is characterized by the replacement of normal liver architecture with regenerative nodules surrounded by fibrous septa, leading to severe disruption of liver structure and function. HCC shows tumor cells with varying degrees of differentiation, often with pseudoglandular structures and vascular invasion. CCA presents as adenocarcinoma with varying differentiation and prominent desmoplastic stromal reaction. Moreover, extensive hepatocellular necrosis, bile duct proliferation, and cellular atypia are observed. These end-stage changes typically signify severe liver failure, reflecting the terminal stage of the disease.^150,154^

While liver biopsy remains the gold standard for assessing liver pathology, its invasiveness and potential complications necessitate the development of non-invasive diagnostic methods. Advances in serological markers and imaging technologies strive to maintain diagnostic precision while reducing patient discomfort and risk, providing efficient alternatives to traditional histological examination.^155^

## Etiology

Various factors can lead to liver injury, including viral and parasitic infections, metabolic disorders, toxic exposures (such as liver-damaging drugs), and genetic predispositions. In this section, we provide a comprehensive summary of the common causes of liver diseases.

### Infection

Acute and chronic liver diseases can be caused by various viruses. HBV, an enveloped DNA virus, infects hepatocytes through bodily fluids, leading to potential CHB, cirrhosis, and HCC due to the persistence of covalently closed circular DNA (cccDNA) in hepatocytes.^156^ HCV can present as acute hepatitis and lead to chronic hepatitis and cirrhosis through interactions with hepatocytes via the envelope glycoprotein E2 and immune evasion mechanisms involving the core protein.^23^ HDV often co-infects with HBV, resulting in more severe liver diseases and increased mortality.^157^ HAV and HEV, transmitted feco-orally, cause self-limiting diseases, with specific mechanism interactions like gangliosides for HAV and immune response dysregulation for HEV impacting infection outcomes.^35,158^ Overall, viral infection serves as a common cause of various liver diseases.

Parasitic infections such as *Schistosomiasis* and *Echinococcosis* primarily occur in rural areas, leading to severe liver complications like fibrosis and portal hypertension via mechanisms like persistent immune response to parasite eggs or direct tissue infiltration by the parasite.^159–161^ Amebic liver abscesses are another consequence, originating from amoebas breaching intestinal barriers and leading to hepatic necrosis.^162^

### Metabolic stress

MASLD represents the hepatic component of a multisystem disorder and is closely linked to the global rise in obesity, T2DM, and metabolic dysfunction.^163,164^ The increasing prevalence of obesity worldwide has significantly heightened the risk of MASLD development. Studies indicate that individuals with a higher body mass index are more prone to MASLD.^165,166^ A recent meta-analysis reported that the prevalence of T2DM among radiologically and histologically defined metabolic dysfunction-associated steatohepatitis (MASH) patients was 22.51% and 43.63%, respectively, underlining the strong association between T2DM and MASLD.^75^ Moreover, various manifestations of metabolic dysfunction, including insulin resistance, bile acid metabolism disorders, gut microbiota imbalances, and hyperuricemia, contribute to the pathogenesis of MASLD.^167–169^ Patients with early MASLD exhibit hepatic steatosis and steatohepatitis, with some progressing to cirrhosis and HCC.^170^ MASLD has emerged as a predominant chronic liver disease with the escalating prevalence of metabolic dysfunction.^171^

### Toxic exposure

In Europe and the U.S., nonsteroidal anti-inflammatory drugs (NSAIDs), anti-infective drugs (e.g., amoxicillin-clavulanate potassium), and herbal/dietary supplements are the most common causes of DILI.^43,172^ However, liver injuries induced by traditional Chinese medicine, anti-tuberculosis drugs, and other anti-infective medications are more prevalent in Asia.^43^ The utilization of anti-cancer drugs and immunomodulators has also been linked to instances of drug-induced liver damage.^173^ Drug risk factors such as dose and metabolism increase the risk of liver injury with certain medications.^174^ Moreover, patient genetic predisposition may be another important determinant.^175^ Notably, DILI is becoming the main cause of ALF worldwide with increasing proportion.^175^ Chronic alcohol consumption leads to ALD, progressing through stages from fatty liver to cirrhosis and HCC, influenced by dosage and individual factors like gender and concurrent conditions like obesity or viral infection.^19,176–180^ Certain chemical substances in industrial production are hepatotropic poisons, which cause a susceptible period in the population. For example, exposure to chloroform and phosphorus has been associated with hepatic histological changes leading to toxic hepatitis.^181,182^ The use of the hepatotoxic substance carbon tetrachloride (CCl_4_) has become a standard method to induce murine liver fibrosis.^183^ Moreover, individuals occupationally exposed to chemicals like vinyl chloride and per- and polyfluoroalkyl substances are more sensitive to MASH.^184,185^ Of note, several fungi species could also produce mycotoxins with high hepatotoxicity, leading to necrosis of hepatocytes with various liver diseases.^186^

### Genetic factors

Hereditary metabolic liver diseases could cause metabolic abnormalities due to the interaction between host and environmental based on genetic defects, mainly including hereditary hemochromatosis, WD, and AATD.^187^ There are 4 types of hereditary hemochromatosis based on gene mutations. Type 1 is a classic hereditary hemochromatosis, known as HFE-associated hemochromatosis. More than 80% of patients have a C282Y mutation or a C282Y/H63D complex heterozygous mutation.^188,189^ These patients show hepcidin and transferrin deficiency, which impairs the transport of iron from intracellular storage sites to plasma, resulting in iron deposition in the liver.^190,191^ WD results from mutations in the *ATP7B* gene, causing dysfunctional copper transport and accumulation.^141^ Excessive copper accumulation triggers a reactive oxygen species (ROS) reaction followed by hepatic inflammation and cirrhosis.^192^ AATD is an autosomal codominant inheritance with the mutation of the *SERPINA1* gene, which encoded alpha-1 antitrypsin. Abnormal conformation of this protein is detained by the rough endoplasmic reticulum, causing cellular stress, and liver disease.^193,194^

## Multi-level regulatory mechanisms

Liver diseases encompass a spectrum ranging from acute and chronic liver injuries to end-stage liver diseases, each characterized with distinct pathogenesis. This section outlines the multifaceted mechanisms underlying the initiation and progression of liver diseases, spanning from molecular and cellular levels to organ interactions.

### Molecular mechanisms-RIG-1/MAVS signaling

**Fig. 3:**
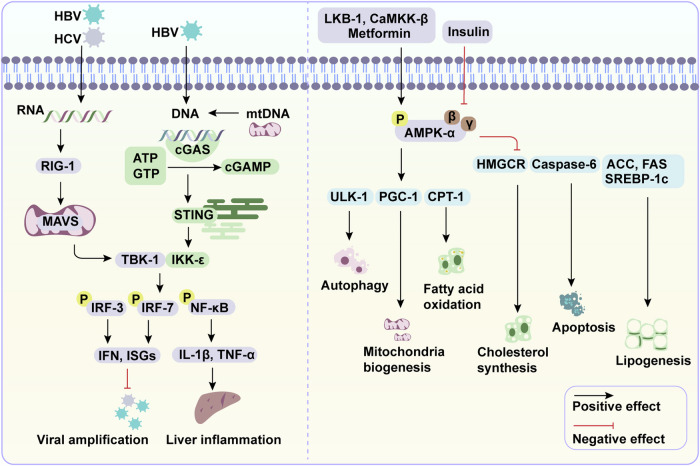
RIG-1/MAVS, cGAS/STING, and AMPK signaling in liver diseases. When hepatotropic viruses such as HCV and HBV infect the liver, RIG-1/MAVS and cGAS/STING signaling pathways are activated. Both pathways promote the expression of IFN and other inflammatory cytokines by the phosphorylation of IRF-3/7 and NF-κB, respectively. These cytokines perform essential roles in antiviral defense and liver inflammation. AMPK, serving as an energy sensor, regulates various cellular physiological processes. Upon exposure to excessive energy or ethanol, decreased AMPK activity and fat accumulation are observed in hepatocytes. However, activation of AMPK in the liver decreases lipogenesis and cholesterol synthesis, and cell apoptosis, while promoting fatty acid oxidation, autophagy flux, and mitochondria biogenesis. These effects help attenuate the development of MASLD and ALD. This figure was generated with Adobe Illustrator

Retinoic acid-inducible gene I (RIG-1) serves as a critical RNA sensor that activates the type I interferon (IFN) response crucial for antiviral defense. RIG-1 is expressed in most cell types and is primarily localized in the cytoplasm.^195^ Upon RNA recognition, RIG-1 undergoes conformational changes and translocates to mitochondria, where it interacts with its adaptor protein, mitochondrial antiviral-signaling protein (MAVS). MAVS then transmits signals from RIG-1 and activates downstream components such as TANK-binding kinase 1 (TBK-1) and IκB kinase-ε (IKK-ε). These kinases prompt the phosphorylation and nuclear translocation of interferon regulatory factor 3 (IRF-3) and IRF-7 as well as nuclear factor-κB (NF-κB), key transcription factors for the production of IFN and other cytokines.^196^ Secreted IFN activates JAK/STAT signaling within host cells to engage IFN-stimulated genes (ISGs) expression, which perform essential functions in antiviral defense and the immune response (Fig. 3).^197^

HCV is a hepatotropic virus associated with liver inflammation, fibrosis, and HCC. The RIG-1 signaling has been demonstrated to play a vital role in HCV sensing and elimination.^198^ Specifically, the HCV RNA genome binds to host RIG-1 to induce IFN production in hepatocytes via RIG-1/MAVS signaling.^199^ This course leads to the production of ISGs like ISG15, protein kinase R (PKR), myxovirus resistance A (MxA), and 2′-5′-oligoadenylate synthetase (OAS), which are known for hindering cell cycle progression and the replication of HCV by impeding viral transcription and translation, initiating viral RNA cleavage, and modifying viral protein functions.^200–203^ However, Li et al. demonstrated that HCV nonstructural protein 3-4A (NS3-4A) protease cleaves MAVS protein and diminishes MAVS/IRF-3-dependent IFN and ISGs production,^204^ whereas NS3/4A inhibitors restore MAVS proteolysis and the IFN-dependent antiviral response.^205^ These data may in part explain the scarcity of endogenous IFN in some HCV-infected individuals and the persistent HCV infection, as well as the importance of RIG-1/MAVS signaling in HCV elimination.

Besides HCV, the HBV DNA is involved in RIG-1/MAVS signaling activation. Pregenomic RNA of HBV is revealed to bind with RIG-1 protein, which induces IFN and ISGs production to prevent HBV infection.^206^ Moreover, RIG-1 counteracts the interaction of HBV polymerase, thus suppressing viral replication and production.^207^ HBV covalently closed circular DNA (cccDNA), the transcriptional template for viral RNA, is indispensable for HBV persistence and chronic hepatitis. Intriguingly, Lee et al. demonstrated that RIG-1/IRF-3 signaling blocks cccDNA formation and amplification in hepatocytes.^208^ These findings underscore the multifunctional roles of RIG-1/MAVS signaling in anti-HBV immune response. However, HBV X protein (HBx) is shown to deactivate IFN production by deubiquitinating RIG-1 and its downstream effectors IKK-ε and IRF-3.^209^ Evidence also suggests that the activation of RIG-1/MAS signaling by other viruses such as HAV, HDV and HEV combats viral replication.^210–212^ In summary, RIG-1/MAVS signaling exerts pivotal roles in limiting virus replication and enhancing innate immune responses, thereby attenuating hepatic viral diseases.

### Molecular mechanisms-cGAS/STING signaling

The cGAS/STING signaling pathway plays a crucial role in immune defense by detecting cytoplasmic DNA. The enzyme cyclic GMP-AMP (cGAMP) synthase (cGAS) is activated upon exposure to cytoplasmic DNA from pathogens or damaged host cells. cGAS synthesizes the second messenger cGAMP, which then binds to and activates the stimulator of interferon genes (STING). STING, localized in the ER, initiates downstream signaling to produce IFNs and other cytokines, thereby promoting a potent innate immune response.^213,214^

In the context of liver disease, cGAS/STING signaling has been implicated in the progression of conditions associated with viral infection and sterile inflammation (Fig. 3). Research indicates that HBV can evade immune surveillance by suppressing DNA sensor pathways, including the cGAS pathway, resulting in reduced expression of cGAS and its effectors during chronic HBV infection. Despite this viral evasion, activation of cGAS signaling can inhibit HBV replication. This is achieved by blocking the amplification of HBV cccDNA and reducing HBV RNA synthesis through the production of STING-mediated cytokines and ISGs, both in vivo and in vitro.^215–217^ Additionally, cGAS/STING signaling has been shown to restrict HCV replication in hepatocytes and is an important component in the immune response against this virus, as observed in studies involving *STING* knockdown models.^218^

The pathway is also involved in the progression of sterile inflammatory liver diseases. The release of endogenous DNA, such as mitochondrial DNA (mtDNA), during cellular damage triggers cGAS-STING signaling. This activation leads to the production of inflammatory cytokines. In cases of liver injury induced by substances such as acetaminophen and thioacetamide, hepatocyte-derived mtDNA activates cGAS-STING signaling in macrophages. This promotes an inflammatory phenotype switch in these cells, which exacerbates hepatocyte injury by promoting ferroptosis, a form of programmed cell death associated with iron.^219–221^ Additionally, mtDNA-induced cGAS-STING signaling has also been reported in mice with ALD^222^ and MASLD,^223^ whereas overexpression of RING finger protein 13 (RNF-13) in hepatocytes attenuates liver steatosis, inflammation and fibrosis by degrading the STING protein in a mouse MASLD model.^224^ Interestingly, the STING-NF-κB pathway in macrophages leads to metaflammation in lean MASLD mouse, which promotes lipolysis in the adipose tissue and subsequently contributes to liver lipid deposition and injury.^225^ The evidence shows the detrimental role of cGAS/STING signaling in the regulation of sterile liver inflammation.

### Molecular mechanisms-AMPK signaling

AMP-activated protein kinase (AMPK) is a highly conserved heterotrimeric protein consisting of a catalytic α subunit and regulatory β and γ subunits. This central eukaryotic energy sensor facilitates the maintenance of physiological cellular processes.^226^ Upon exposure to excess energy, liver kinase B1 (LKB-1) and Ca^2+^/CaM-dependent protein kinase kinase β (CAMKK-β), phosphorylate the Thr172 residue on the AMPK α subunit.^227^ Then AMPK phosphorylates and stimulates multiple downstream substrates to regulate lipid and glucose metabolism, as well as mitochondrial function.^228^ Herein, we propose the primary mechanisms by which AMPK affects liver injury, especially in MASLD and ALD (Fig. 3).

AMPK inhibits lipid synthesis by deactivating acetyl-CoA carboxylases (ACC-1 and ACC-2) and HMG-CoA reductase (HMGCR), the rate-limiting enzymes in fatty acid and cholesterol synthesis, respectively.^229^ There is a negative correlation between AMPK and the development of MASLD and ALD, as shown by the reduced AMPK levels in these fatty liver samples.^230,231^ Notably, activation of AMPK by its upstream kinase LKB-1, restores hepatic lipid accumulation by downregulating lipogenesis-mediated genes, such as *Srebp1c*, *Acc*, *Fas*, *Scd1*, and *Hmgcr* in a high-fat diet-induced mouse model.^232^ SREBPs, key transcriptional factors for lipid and cholesterol synthesis, are verified to contribute to MASLD and MASH-associated HCC.^233,234^ Recently, studies showed that maturation and activity of SREBPs are controlled by adenosine A1 receptor (A1R) and A2R, which could be targeted to relieve MASLD.^234,235^ Specific agonist of A1R, 2-chloro-N6-cyclopentyladenosine (CCPA) or screened natural compound, timosaponin AIII showed promising activity in MASLD, especially MASH therapy by activating hepatic A1R.^234^ Therefore, hepatic A1R is a novel target for MASLD/MASH therapy with great potential through modulating SREBPs maturation and its controlled fatty acid de novo synthesis. Conversely, in vivo and in vitro studies found that hepatocytes exhibit more severe triglyceride accumulation when AMPK is depleted or blocked by its inhibitor compound C, showing the diminished protective role of AMPK against liver steatosis.^236^ These data indicate the pivotal role of AMPK signaling in blocking hepatic lipogenesis.

Furthermore, AMPK promotes the expression of genes related to fatty acid oxidation in an ACC-2-dependent manner. Specifically, AMPK-inhibited ACC-2 catalyzes the generation of malonyl-CoA, which inhibits the activity of carnitine palmitoyltransferase 1 (CPT-1), a rate-limiting enzyme of mitochondrial oxidation.^237^ Upon exposure to ethanol and lipids, decreased AMPK activity and fatty acid oxidation flux is observed in mouse hepatocytes.^238,239^ In contrast, metformin-driven AMPK activation rescues CPT-1 expression and diminishes lipid accumulation in rat livers impacted by chronic ethanol insult.^240^ Likewise, CAMKK-β-induced AMPK activation promotes fatty acid oxidation and mitochondrial biogenesis, thereby attenuating hepatic steatosis in MASLD mice.^241^

AMPK is essential to maintain mitochondrial homeostasis. Upon inflammatory stimuli, decreased AMPK activity together with mitochondrial dysfunction and ROS is observed in MASH models.^242^ Growing studies showed that ethanol-induced ROS could be repressed in an AMPK-dependent manner. Impaired mitochondrial structure and increased mtDNA were observed in ethanol-treated hepatocytes, and AMPK is verified to rescue mitochondrial biogenesis and function by increasing mitophagy and ROS removal in hepatocytes.^231,243^ High-fat diet-induced ROS and ER stress were inhibited by activating AMPK/NRF-2/HO-1 signaling, which attenuated hepatic lipid accumulation and inflammation.^244–246^ In addition, both in vivo and in vitro experiments demonstrated that AMPK-Caspase-6 axis relieves mitochondrial function and protect against hepatocellular apoptosis in MASH, as well as ferroptosis in ALD.^247,248^ Besides, AMPK plays important roles in linking metabolism to the development of liver cancer. Lower levels of AMPK are associated with poor prognosis in HCC, while activation of AMPK expression regulates metabolic reprogramming in the tumor microenvironment, improving the efficacy of tumor immunotherapy.^249,250^ Collectively, the above evidence elucidates the crucial roles of AMPK in liver metabolism and inflammation, which implicates AMPK might be a potential therapeutic target in liver diseases.

### Molecular mechanisms-MAPK signaling

**Fig. 4:**
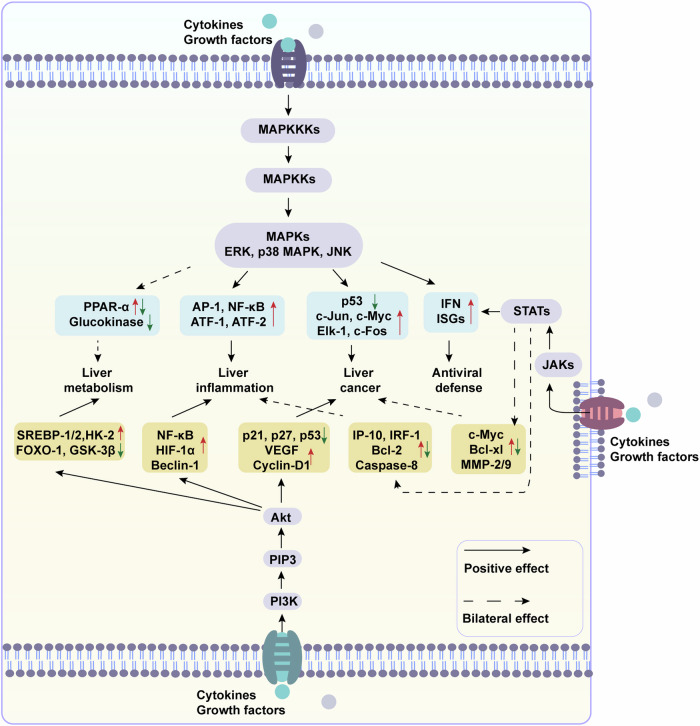
MAPK, PI3K/Akt, and JAK/STAT signaling in liver injury. When liver injury occurs, activated MAPK signaling promotes gluconeogenesis but has bilateral effects on liver lipid metabolism. It enhances liver inflammation by targeting key transcription factors, including activator protein 1 (AP-1) and NF-κB, and promotes HCC and ICC by encouraging tumor cell proliferation and migration. Additionally, MAPK plays a role in antiviral defense by activating the expression of IFN and ISGs. PI3K/Akt signaling promotes liver lipid and glucose metabolism by targeting key transcription factors and enzymes, and similarly promotes liver inflammation and cancer. JAK/STAT signaling is essential for the elimination of hepatotropic viruses through the induction of IFN and ISGs. STAT proteins have bidirectional roles in liver inflammation and cancer. Specifically, STAT1 promotes inflammation, whereas STAT3 exhibits both pro-inflammatory and anti-inflammatory signals. STAT1 prevents HCC development, whereas STAT3 contributes to the liver tumorigenesis. This figure was generated with Adobe Illustrator

The mitogen-activated protein kinase (MAPK) signal transduction pathway is a critical mediator that orchestrates cellular proliferation, differentiation, and death. The core module of MAPK signaling is composed by three-tiered kinase cascade proteins, namely MAPK kinase kinase (MAPKKK), MAPK kinase (MAPKK) and MAPK. MAPKs encompass extracellular signal regulated kinases (ERK-1/2), p38α/β/δ/γ MAPK, and c-Jun-N-terminal kinases (JNK-1/2/3).^251^ MAPK signaling is activated in response to extracellular stimuli, including hormones, cytokines, growth factors. Active MAPK phosphorylates and activates downstream effectors, including transcription regulators that translocate to the nucleus to manipulate target gene expression.^252^ In the liver, MAPK signaling plays an important role in mediating inflammation, metabolism, and cell proliferation (Fig. 4).

Multiple studies have revealed the activation of MAPK signaling in hepatocytes and macrophages during acute or chronic liver injury. Fatty acids, ethanol, and acetaminophen have been implicated in the activation of p38 MAPK, JNK and ERK signaling in hepatocytes.^253–255^ The signaling cascade triggers the activation of critical regulators of lipid synthesis, autophagy, and inflammation such as SREBP-1c, sequestosome-1 (SQSTM1/P62) and NF-κB; however, blocking JNK and p38 signaling by degradation of TAK-1, a member of the MAPKKK family, reverses fat accumulation, impaired autophagy flux, inflammation, and apoptosis in hepatocytes.^253^ Recently, lysosomal homeostasis and autophagic flux have been recognized as playing a beneficial role in MASLD.^256^ Lysosomal dysfunction can lead to impaired autophagic flux, inducing lipid droplet accumulation in hepatocytes and further activating HSCs in a hepatic steatosis model.^257,258^ In contrast, hepatic fat accumulation and liver fibrosis are alleviated when lysosomal dysfunction is restored.^259,260^ Additionally, both in vivo and in vitro studies have demonstrated that the overexpression of JNK signaling and subsequent AP-1 and NF-κB cascades in hepatocytes promotes cell proliferation and migration, thus contributing to MASLD-associated liver cancer.^261^ The above evidence suggests that MAPK signaling and its downstream effectors plays pivotal roles in hepatocyte survival and function.

Emerging studies suggest that MAPK signaling in hepatic macrophages acts as a key contributor to liver injury. p38 MAPK signaling in macrophages contributes to the development of nutritional steatohepatitis by promoting M1 macrophage polarization and the release of inflammatory cytokines. In contrast, macrophage p38 MAPK deficiency in mice is associated with a hepatic M2 phenotype characterized by decreased secretion of TNF-α, IL-6, and CXCL-10, which leads to reduced fat accumulation and hepatocyte apoptosis.^262^ Consistently, macrophage p38 MAPK-deficient mice are more resistant to drug-induced hepatotoxicity, as evidenced by decreased cytokine production and accelerated hepatocyte regeneration.^263^ Moreover, ERK signaling in macrophages is responsible for TGF-β production, thus triggering HSC activation in response to high-fat/high-cholesterol diet.^264^ Interestingly, HSC activation is directly promoted by ERK signaling. Specifically, the secretory protein ANGPTL8 from fatty hepatocytes interacts with the LILRB2 receptor on HSCs and activates ERK signaling-dependent autophagy.^265^ Increased autophagy flux facilitates the transdifferentiation of HSCs into a myofiblastic phenotype, ultimately contributing to liver fibrogenesis.^266^ These observations implicate the importance of MAPK signaling in the evolution of liver diseases.

### Molecular mechanisms-PI3K/Akt signaling

The phosphoinositide 3-kinase (PI3K)/protein kinase B (Akt) pathway represents an evolutionarily conserved signaling cascade pivotal in cellular processes such as metabolism, survival, proliferation, and cell death. The pathway consists of two core components: PI3Ks and Akts. Upon stimulation, PI3K catalyzes phosphatidylinositol 4,5-bisphosphate (PIP2) to generate phosphatidylinositol 3,4,5-trisphosphate (PIP3), which serves as a second messenger to recruit and activate Akt. Activated Akt then phosphorylates numerous downstream substrates to initiate multiple pathways. In this section, we focus on PI3K/Akt signaling during liver diseases (Fig. 4).^267^

PI3K/Akt signaling performs bidirectional roles in response to acute liver injury and liver fibrosis.^268,269^ PI3K/Akt signaling impedes liver regeneration by promoting macrophage migration and fostering an inflammatory environment after partial hepatectomy.^270^ On the other hand, PI3K/Akt signaling is responsible for the production of hepatocyte growth factor (HGF), epidermal growth factor (EGF), and TGF-β, which are essential for hepatocyte proliferation and survival.^271^ In the setting of liver fibrosis, PI3K/Akt signaling in macrophages contributes to profibrotic mediators secretion, thus triggering HSC activation and ECM production.^272^ However, another study revealed that PI3K/Akt signaling counteracts the TGF-β/SMAD signaling-an important player in HSC activation-to balance cell survival and proliferation under chronic stimuli.^273^ In addition, activated Akt induces the expression of matrix metalloproteinase (MMPs), which plays an importance role in ECM breakdown.^274^ All these data demonstrate the dual roles of PI3K/Akt signaling in liver diseases. Considering the different stages of liver diseases, as well as the diverse cellular sources of PI3K/Akt signaling, these controversial results should be interpreted cautiously.

PI3K/Akt signaling is associated with the development of HCC through its regulation of tumor cell glycolysis, growth, and apoptosis. HCC cells exhibiting activated PI3K/Akt signaling show increased glucose uptake and lactate production, a phenomenon known as aerobic glycolysis or the Warburg effect, facilitating long-term cancer cell survival. In contrast, suppression of PI3K/Akt signaling transitions aerobic glycolysis to oxidative phosphorylation, accompanied by restored mitochondrial function, which indicates the involvement of PI3K/Akt signaling in metabolic reprogramming during HCC progression.^275^ In addition, inhibition of PI3K/Akt signaling elicits increased expression of caspase-3 and caspase-9, apoptotic markers, within HCC cells.^276^ Overall, these findings suggest the excitatory role of PI3K/Akt signaling in the evolution of HCC.

### Molecular mechanisms-JAK/STAT signaling

The Janus kinase /signal transducer and activator of transcription (STAT) signaling pathway is a highly conserved pathway that performs crucial roles in cell differentiation, metabolism, growth, and immune response.^277^ Once extracellular signals such as cytokines, interferons, and growth factors, bind to their respective receptors, JAK proteins and downstream STAT proteins undergo phosphorylation. Activated STAT proteins translocate to the nucleus, where they bind to DNA sequences to regulate target gene expression.^278,279^ In this context, we discuss the dysregulation of the JAK/STAT signaling in liver diseases, particularly in autoimmune and viral hepatitis (Fig. 4).

Upregulated JAK/STAT signaling has been observed in patients with PBC, a chronic autoimmune liver disease.^280^ Genome-wide meta-analysis suggested a correlation between JAK/STAT signaling and PBC.^281^ Importantly, a JAK-1/2 inhibitor, baricitinib, has shown promising results in reducing alkaline phosphatase (ALP) levels and liver inflammation in PBC patients based on a phase II trial.^282^ Mechanistically, IFN-induced JAK/STAT1 signaling triggers the amplification of hepatic CD4^+^ T cells and CD8^+^ T cells, the polarization of M1 macrophages and the release of cytokines in experimental autoimmune cholangitis models, eliciting a liver immune response and inflammation.^283^ This finding underscores the pivotal role of JAK/STAT signaling in modulating liver autoimmunity.

In addition, JAK/STAT signaling is implicated in IFN-induced viral hepatitis. As mentioned above, secreted IFN binds to its receptor and activates ISG expression in a JAK/STAT-dependent manner.^284^ Essential ISGs such as PKR and OAS, which are crucial for restricting HBV replication, are induced by JAK/STAT signaling, whereas the inhibition of this pathway leads to diminished PKR and OAS expression during HBV infection.^285^ Similarly, in vivo and in vitro studies revealed that the JAK/STAT-dependent induction of ISG-12a plays a vital role in inhibiting HCV replication.^286^

In addition to ISGs, numerous mediators involved in liver inflammation and fibrosis are also the target of JAK/STAT signaling.^287^ The activation of JAK/STAT signaling in hepatocytes mediates the production of IL-6, CXCL-10, and iNOS, which promotes hepatocyte apoptosis, inflammatory cell infiltration and fibrogenesis in a mouse model of MASLD.^288,289^ In summary, JAK/STAT signaling has dual roles in liver diseases: stimulating innate and adaptive immunity while governing virus elimination within distinct disease contexts.

### Molecular mechanisms-Wnt/β-catenin signaling

**Fig. 5:**
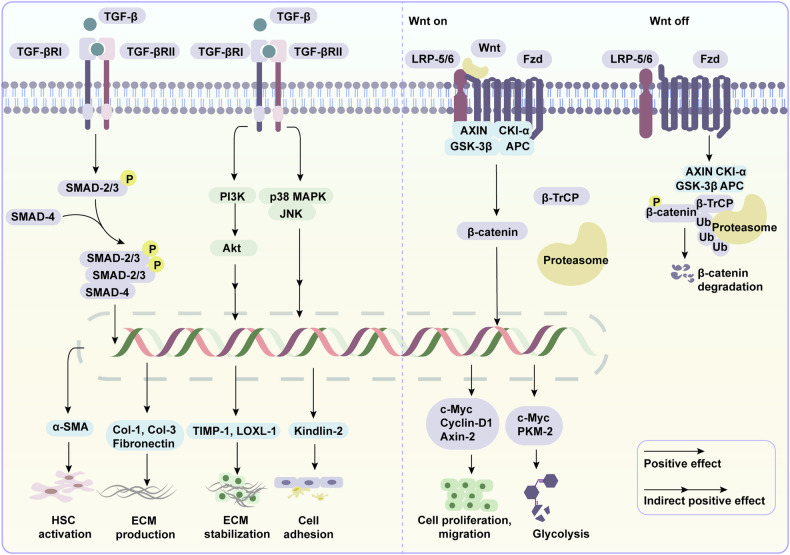
TGF-β and Wnt/β-catenin canonical signaling in liver diseases. Upon liver injury, TGF-β secreted from other liver cell types binds to its receptor TGF-βRII, which subsequently recruits TGF-βRI to synergistically mediate downstream pathways: canonical SMAD-dependent pathway and non-SMAD pathways. In canonical pathway, the SMAD oligomers translocate into the nucleus, where they function as transcription factors, mediating the transcriptional activation. In non-SMAD pathway, PI3K/Akt and MAPK pathways are activated by TGF-βRs. These pathways induce expression of genes, such as α-SMA, Collagens, fibronectin, TIMP-1, LOXL-1, and Kindlin-2, leading to HSC activation, ECM production and stabilization, and cell adhesion. These processes collectively promote liver fibrosis. In a healthy liver, Wnt signaling is typically inactive due to the absence of Wnt-Wnt receptor interactions and the degradation of β-catenin by a protein complex, which includes axis inhibition protein (AXIN), adenomatous polyposis coli (APC), and E3 ubiquitin ligase. During liver oncogenic injury, Wnt proteins bind to Fzd receptor and LRP-5/6 co-receptors, activating the canonical pathway. This activation causes degradation complex to translocate to the cell membrane, preventing the degradation of β-catenin. The β-catenin then enters the nucleus, where it binds with TCF/LEF transcription factors to regulate target gene expression, such as c-Myc, cyclin-D1 and pyruvate kinase M2 (PKM-2). These genes are involved in promoting tumor cell metabolism, proliferation, migration, and metastasis in HCC and ICC. This figure was generated with Adobe Illustrator

The Wnt/β-catenin signaling performs vital functions in embryonic process and organ development. Upon Wnt proteins bind to frizzled receptors (Fzd) and low-density lipoprotein receptor-related protein 5/6 (LRP-5/6) co-receptors, signals are transduced to β-catenin to trigger downstream events. Under physical conditions, the signaling is tightly regulated through degradation complex in the cytoplasm, where a multiprotein complex involving enzymes such as E3 ubiquitin ligases leads to the degradation of signaling proteins, maintaining an inactive state.^290^ Dysregulation of Wnt/β-catenin signaling is currently considered as a crucial factor in oncogenesis. The accumulation and nuclear shuttling of β-catenin result in its interaction with T-cell factor/lymphoid enhancer factor (TCF/LEF) transcription factors, activating proto-oncogenes such as myelocytomatosis oncogene (c-Myc) and cyclin-D1 (CCND-1) and thus promoting cell proliferation and migration.^291^ Here, we summarize the crucial roles of Wnt signaling in liver cancer (Fig. 5).

Genetic mutations involving Wnt signaling have been reported in human HCC. Approximately 8-30% of HCC patients exhibit mutations in the β-catenin gene (*CTNNB-1*), which prevents β-catenin degradation and facilitates its nuclear translocation.^292,293^ Integrated multi-omics analyses have revealed pathologically elevated Wnt signaling in human HCC tissues.^294^ Notably, the interaction between Wnt-3a and Fzd-7 in human HCC cells drives tumor proliferation and migration by activating β-catenin-dependent signaling.^295^ Intriguingly, hepatocyte-specific overexpression and activation of β-catenin protein alone are insufficient to induce HCC.^296^ However, the stimulation of β-catenin in conjunction with pathological stimuli could initiate and accelerate HCC progression in mice.^297^ In contrast, the prevalence of tumor in the liver with *Ctnnb-1* conditional knockout is 7-fold higher than that in wild type liver, indicating that the absence of β-catenin stimulates carcinogen-induced hepatocarcinogenesis.^298^ Mutation of hepatic *Ctnnb-1* drives hepatocarcinogenesis by upregulation of pro-tumorigenic cytokines.^299^ It seems contradictory that the presence of the mutated β-catenin and the absence of normal β-catenin, both contribute to the development of HCC. More in-depth studies are needed to clarify the precise mechanism.

In addition to its role in HCC, Wnt/β-catenin signaling also participates in the initiation and progression of intrahepatic cholangiocarcinoma (ICC). Notably, upregulated Wnt-7b levels are observed in human ICC tumors and mouse ICC models, with evidence suggesting that macrophages are the cellular source of Wnt-7b production in vivo and in vitro.^300^ Pharmacological or genetic inhibition of Wnt-7b-Fzd7-β-catenin signaling has shown promise in mitigating tumor growth and metastasis.^301^ In summary, Wnt/β-catenin signaling contributes to the oncogenic process of liver carcinoma.

### Molecular mechanisms-TGF-β signaling

Transforming growth factor-β (TGF-β) is a cytokine with three isoforms (TGF‑β1, TGF‑β2, and TGF‑β3), sharing around 80% homology in their amino acid sequences. Upon TGF-β binding, TGF-β receptor 2 (TGF-βR2) recruits and activates TGF-βR1 to synergistically mediate downstream signaling. TGF-β/TGF-βR transmits extracellular stimuli and exhibit cellular transcriptional events by two ways: canonical SMAD-dependent pathway and non-SMAD pathways.^302^ TGF-β signaling is well-recognized for inducing fibrosis in multiple organs, including the liver (Fig. 5).^303,304^

Excessive TGF-β expression is documented in both acute and chronic liver diseases across various cell types.^305^ In patients with diseases such as AIH and chronic hepatitis C, increased serum and hepatic levels of TGF-β are observed, correlating with disease progression.^306,307^ Transcriptome analysis from MASLD model reveal macrophages, LSECs, activated HSCs, and hepatocytes as sources of TGF-β production.^308^ Notably, macrophages are identified as the predominant cellular origin of TGF-β in the injured liver.^309^

HSC activation serves as a hallmark event in the initiation of liver fibrosis, with ECM deposition characterizing fibrotic progression. TGF-β, which is induced by liver injury, triggers TGFR activation in HSCs, leading to phosphorylation of downstream effectors, such as small mothers of decapentaplegic (SMAD) proteins. Activated SMAD proteins translocate into the nucleus, where they facilitate transcription of target genes by interaction with DNA-binding transcription cofactors.^310^ Literature supports that TGFR/SMAD in HSCs promotes expression of α smooth muscle actin (α-SMA), collagen type I and III, which are involved in HSC activation and extracellular matrix (ECM) composition, respectively.^311,312^ In addition, SMAD also triggers the expression of lysyl oxidase-like (LOXL) and tissue inhibitor of metalloproteinases (TIMP) proteins, both of which perform essential functions in ECM deposition and stabilization.^313,314^ In contrast, mice with HSC-specific inactivation of SMAD-2 have increased susceptibility to CCl_4_- and DDC-induced liver fibrosis.^315^

In addition to the canonical pathway, TGF-β also contributes to liver fibrosis through the non-SMAD pathway by interplaying with MAPK signaling and PI3K signaling. Recent work has elucidated interactions between TGF-β and p38 MAPK signaling in HSCs, driving kindlin-2 expression and subsequent immune cell adhesion, which in turn promotes HSC activation.^316^ In addition, TGF-β induces ADAM12 expression via the PI3K/Akt pathway in cultured human HSCs, contributing to cell adhesion and migration.^317^ Taken together, these data indicate the critical roles played by both SMAD and non-SMAD pathways in TGF-β-induced HSC activation and liver fibrosis.

### Inter-cellular mechanisms

Cellular crosstalk is a crucial event that maintains liver homeostasis. When liver injury occurs, hepatocytes and non-parenchymal cells engage in pathological paracrine interactions. In this section, we describe the cellular crosstalk during liver diseases.

### Hepatocytes

Hepatocytes, major parenchymal cells in the liver, perform diverse roles in lipid and glucose metabolism, detoxification, and protein synthesis. During disease states, hepatocytes face direct assaults from viruses or metabolites but also respond to signals from neighboring cells. The injury imposed on hepatocytes may exacerbate cellular dysfunction and subsequent death.^318^ Liver macrophages serve as primary reservoirs for inflammatory cytokines such as IL-1β and TNF-α, which contribute to hepatocyte death.^319^ It has been well-established that IL-1 drives hepatocyte inflammation and apoptosis via interacting with its receptor and downstream effectors. In contrast, hepatocyte-specific depletion of IL-1 receptor rescues hepatocyte apoptosis by blocking JNK/NF-κB signaling during acute liver injury.^320^ Inflammasomes released from macrophages also contribute to hepatocyte pyroptosis and liver inflammation in mouse model of MASLD.^320^ In addition, Wnt2 protein derived from LSECs was found to regulate cholesterol and bile acid homeostasis in hepatocytes.^321^ However, silencing the expression of LSEC-specific Wnt2 disturbs hepatocyte metabolic profiles in both acute and chronic liver injury, indicating the essential role of LSECs in hepatocyte function.^322^

### Cholangiocytes

Cholangiocytes are highly specialized epithelial cells forming the bile ducts and are essential for bile acid homeostasis. In chronic liver injury, such as cholangiopathies, a pathological feature known as a ductular reaction occurs, characterized by the proliferation of reactive ductular cells.^323^ Ablation of β1-integrin in hepatocytes stimulates the ductular reaction, leading to cholangiocyte-derived hepatocyte regeneration during chronic liver diseases. These data indicate the potential network between hepatocytes and cholangiocytes.^324^ Inflammatory and fibrotic secretions from immune cells are involved in cholangiocyte activation and biliary repair, which in turn, leads to increased inflammatory cell infiltration and persistent liver impairment.^325,326^

### LSECs

LSECs are highly differentiated endothelial cells lining the liver sinusoids. LSECs possess a unique phenotype with fenestrae, enabling substantial exchanges and cellular communications.^327^ Although LSECs detect extrahepatic signals and help maintain liver homeostasis through angiocrine mechanisms, their function and architecture are regulated by other cell types. Inflammatory cell populations, such as CCR2^+^ macrophages and CXCR1^+^ neutrophils, which are recruited by injured LSECs in the early phase of liver damage, could in turn compromise LSEC endocytosis capacity and cause its fenestrae impairments.^328,329^ Moreover, neutrophil adhesion attracts platelet recruitment, leading to the generation of sinusoidal microthrombi, which in turn induces sinusoidal dysfunction and vasoconstriction.^330^ This pathological cascade elevates sinusoidal pressure, exacerbating liver fibrosis and portal hypertension. HSCs are crucial players to maintain LSEC phenotype. Bone morphogenetic protein 9 (BMP-9) has been identified to control vessel homeostasis.^331^ Intriguingly, HSCs are the hepatic cell source of BMP-9, which emphasize the impact of HSC-derived BMP-9 on LSEC phenotype and function via targeting its receptor activin receptor-like kinase 1 (ALK-1).^332^ Aberrant expression of BMP-9 or ALK-1 depletion in LSECs during diseased states results in impaired angiocrine function and LSEC architecture, underlining the effect of HSCs on LSEC physiological process.^333^

### HSCs

Quiescent hepatic stellate cells (qHSCs) represent about 5% of liver resident cells and reside in the space of Disse. Following liver damage, qHSCs convert to an activated myofibroblast phenotype characterized by proliferation, contractility, and chemotaxis.^334^ Fate tracing analysis has implicated that activated HSCs are the predominant source of ECM in liver diseases induced by toxic, fatty, and cholestatic insults.^335,336^ These activated HSCs migrate to the injured sites, where they proliferate and contribute to ECM production, thus participating in liver repair. Multiple mediators from other liver cell types contribute to HSCs activation. LSECs are responsible for the production of TGF-β and activation of HSC via the angiocrine pathway during the early stage of liver injury.^337,338^ Besides canonical TGF-β signaling, platelet-derived growth factor (PDGF) and IL-6 produced by capillarized LSECs also contribute to HSC activation by the JAK/STAT pathway.^339^ Notably, macrophages are the major producers of TGF-β, leading to subsequent HSC activation during liver injury. Loss of TGF-β results in a significant decrease in ECM deposition.^340^ NLRP3 inflammasome, induced by pyroptotic hepatocytes, also activates HSCs by releasing multiple cytokines in CCl_4_-induced liver injury.^341^ In summary, various liver cell types are responsible for HSC activation to induce liver fibrosis.

### Macrophages

As the predominant immune cell type in the liver, macrophages play crucial roles in maintaining liver homeostasis and responding to diseases. Hepatic macrophages are composed by liver-resident Kupffer cells (KCs) and monocyte-derived macrophages (MoMFs), two heterogeneous subpopulations with distinct functions.^342^ KCs primarily serve as the main source of hepatic macrophages in a healthy liver, responsible for sensing injury signals and clearing cellular debris.^343^ Under inflammatory conditions, MoMFs infiltrate and become the major component of hepatic macrophages with the loss of KCs.^344^ Macrophages play essential roles in liver inflammation and fibrosis via the production of inflammatory cytokines and chemokines and the activation of inflammasomes.^345^ LSECs recruit MoMFs in a CCL2/CCR2-dependent manner during chronic liver injury. Specifically, by deleting LSEC-specific CCL2, infiltrating macrophage recruitment, liver inflammation, and fibrosis were reduced in CCl_4_-induced mice.^328^ In addition, transcriptome analysis and animal experiments reveal that hepatocytes are involved in the recruitment of MoMFs via the CCL2-CCR2 interaction in acute liver injury to facilitate necrotic lesion resolution.^346,347^ Besides, mediators secreted by hepatocytes, such as IL-17, IL-1β, and extracellular vesicles (EVs), mediate inflammatory macrophage infiltration and the development of ALD and MASLD.^348,349^ However, certain inflammatory macrophages switch to an anti-inflammatory LY6C^low^ phenotype during the progression of liver diseases, which plays an important role in ECM degradation via secretion of MMPs.^350^ Moreover, targeting myeloid-derived RNF-41 has been shown to promote this phenotypic switch and subsequent ECM degradation, thus leading to fibrosis resolution.^351^ While the data indicate the dual roles of macrophages, further investigations are needed to elucidate the precise mechanisms of macrophage phenotype switch during liver inflammation and fibrosis.

### Other immune cells

The T cell-mediated adaptive immune response plays central roles in antigen-driven liver diseases such as autoimmune hepatitis and chronic viral hepatitis.^352^ Autoantigens in hepatocytes, such as formiminotransferase cyclodeaminase, and cytochrome P450 2D6, are presented in the naive CD4^+^ T-helper lymphocytes. These Th0 cells subsequently differentiate into Th1 and Th2 cells, which are responsible for macrophage and B cell infiltration, respectively. This immune response cascade attacks hepatocytes, contributing to liver injury.^353,354^ Similarly, the T cell response is also pivotal in the clearance of HBV and HCV. Effector T cells recognize HBV/HCV-infected hepatocytes and eliminate the virus through a combination of cytotoxic and non-cytotoxic pathways.^355,356^

In a healthy liver, neutrophils are typically absent, but their infiltration within liver sinusoids is noted during acute and chronic liver diseases.^357^ Research has highlighted that LSEC-dependent neutrophils infiltration occurs through the secretion of CXCL chemokines.^358^ Growing evidence has emphasized the significance of neutrophil in liver diseases, as they are thought to promote liver inflammation by releasing cytokines and chemokines, along with recruiting various other immune cells and potentially contributing to the formation of microthrombi.^359,360^ Collectively, these findings indicate that immune cells play multifaceted roles in the progression of liver diseases, impacting both the immune response and inflammatory processes within the liver.

### Liver-organ communication

Emerging evidence has shown that pathological changes at the molecular and cellular levels may not fully account for the pathogenesis of liver diseases, implying a potential role for inter-organ communications in their development. Recently, the gut-liver-brain and adipose-liver homeostasis has gained much attention.^361^ In this section, we summarize the recent research on the importance of liver-organ interactions concerning metabolism, immune system, and nervous system.

### Gut-liver-brain axis

Numerous preclinical and clinical studies have highlighted the role of abnormal gut microbiota and their metabolites in impairing intestinal barrier function, further contributing to liver diseases. Increased permeability is reported in mice fed a high-fat diet or with excessive ethanol intake.^362,363^ Gut leakage leads to the delivery of pathogens and their metabolites to the liver via portal vein. In patients with MASH, gut microbiomes predominantly exhibit Gram-positive *Firmicutes*. However, there is a decrease in *Firmicutes* and an increase in Gram-negative *Proteobacteria* abundance as liver fibrosis develops.^364^ Animal studies demonstrate that germ-free mice fed a high-fat diet show reduced liver steatosis and insulin resistance compared to wild-type mice on the same diet.^365^ This beneficial effect disappears following fecal microbiota transplantation (FMT) from MASLD mice to germ-free mice, resulting in increased liver triglyceride content and inflammation.^366^ These data indicate that dysregulated gut microbiota is essential for the progression of MASLD. Besides pathogenic bacteria, their metabolites also mediate liver diseases. For instance, short chain fatty acids (SCFAs) produced by gut bacteria have been linked to promoting hepatic de novo lipogenesis and glucose production, exacerbating the development of MASLD and ALD in mouse models.^367,368^ In contrast, acetate, another type of SCFA, could block the IL-6/JAK1/STAT3 signaling pathway via binding to hepatocyte-derived GPR43, and reverse the development of MASLD-associated HCC.^369^ This suggests the complex functions of SCFA components on liver injury. In addition, gut bacteria-derived lipopolysaccharides (LPS) influxes to the liver and activates KCs or macrophages expressing toll-like receptors (TLRs). These immune cells produce inflammatory cytokines and chemokines, thus augmenting innate immune responses and liver injuries.^370,371^ On the other hand, the normal enterohepatic circulation of bile acids (BAs) is important for liver and intestinal homeostasis. BA is secreted by hepatocytes and modified in the intestine for lipid digestion and absorption. Upon liver injury, changes in BA composition and level, as well as decreased BA receptor (farnesoid X receptor; FXR) was reported.^372^ Studies show that a decrease in intestinal FXR levels dampens the tight junctions of intestinal epithelial cells and promotes intestinal lipid absorption.^373,374^ Additionally, knockdown of hepatocyte-derived FXR increases hepatic triglyceride content. In contrast, activation or overexpression of FXR protects against liver injury by decreasing hepatic lipogenic gene expression, reducing intestinal lipid absorption, and promoting intestinal barrier integrity.^374,375^

The liver-brain axis is essential in the context of liver diseases. Upon acute or chronic injury, liver produces inflammatory cytokines and is unable to process ammonia from the intestine.^376,377^ These cytokines, such as TNF-α and IL-1β, impair blood-brain barrier (BBB) function and structure, and lead to neuroinflammation.^378^ In addition, lipid components such as ceramide and palmitate, along with peripheral insulin resistance in MASLD preclinical models, contribute to neuroinflammation and neurodegeneration.^379^ Moreover, ammonia crosses the damaged BBB and is absorbed by astrocytes, leading to the conversion of ammonia to glutamine and subsequently causing cerebral edema and neuronal cell death.^380^ Reciprocally, the central nervous system (CNS) exerts an influence on liver and intestine function. Changes in liver microenvironment are detected and transduced through hepatic vagal sensory afferent nerves to CNS, which feeds back the signal to liver vagal parasympathetic nerves.^381^ For instance, CNS leptin signaling has been shown to promote hepatic triglyceride export and inhibit lipogenesis via the brain-vagus-liver axis, thereby attenuating the development of MASLD in animal models, as well as in a randomized, placebo-controlled crossover trial.^382,383^ Chronic systemic inflammation involving liver-organ interactions, as well as bacteria translocation due to impaired intestinal barrier can further lead to acute-on-chronic liver injury and multiorgan failure.^384^ In summary, dysregulation of the gut-liver-brain axis partially influence the progression of liver diseases, suggesting potential therapeutic strategies targeting this axis for liver disease management.

### Adipose-liver axis

Emerging studies have demonstrated the pathological crosstalk between the liver and adipose tissue during liver diseases, especially MASLD and ALD. Fat overload leads to adipocyte hypertrophy, hyperplasia, and abnormal adipokine production.^385^ Chronic ethanol exposure also disrupts the endocrine function of adipose tissue.^386^ For example, adiponectin, which promotes liver glucose use and fatty acid oxidation, is found to be decreased in MASLD and ALD; however, exogenous supplementation of adiponectin alleviates high-fat diet- and ethanol-induced liver steatosis and insulin resistance.^387^ Leptin, an adipocyte-derived hormone that inhibits appetite and increases fatty acid oxidation, becomes resistant in MASLD. In obese individuals, higher serum leptin levels correlate with greater severity of liver inflammation.^388^ In contrast, inflammatory cytokines such as IL-1β and TNF-α are produced by these diseased adipocytes. The inflammatory microenvironment recruits immune cell infiltration and further promotes adipocyte lipolysis. Systemic release of fatty acids and cytokines flows into the liver and aggravates liver steatosis and inflammation.^389^

Conversely, the liver also interacts with adipose tissue. Fibroblast growth factor-21 (FGF-21), an endocrine hormone mainly produced by hepatocytes, contributes to glucose uptake and adiponectin production in adipocytes.^390^ In obesity, the expression of FGF-21 increases with the progression of MASH, whereas its effect on adipose tissue becomes resistant, as evidenced by decreased levels of adiponectin.^391^ As mentioned above, a decrease in adiponectin exacerbates the development of MASLD and ALD by promoting liver steatosis and insulin resistance. In summary, pathological crosstalk between the liver and adipose tissue contributes to liver diseases.

## Diagnosis, staging, prevention and therapeutic strategies

The diagnosis of liver disease usually includes the following steps: history collection, physical examination, laboratory tests, imaging examination, and histopathological examination. For several kinds of liver diseases, a liver biopsy may be required to confirm the diagnosis by pathological examination under a microscope. In the process of diagnosis, it is necessary to select targeted examination items according to the specific conditions of the patient to clarify or exclude the disease (Table 2). The accurate diagnosis of etiology and clinical staging is crucial for guiding treatment strategies and improving patient prognosis. Therefore, we have summarized the current diagnostic principles and staging plans for various liver diseases, while also providing an introduction to the corresponding treatment strategies as outlined in the guidelines.

**Table 2:** Summary of updated diagnosis for liver diseases

Disease category	Medical history/physical examination	Diagnostic criteria	Clinical classification
Viral hepatitis	1. Acute: presenting with abdominal pain, nausea, vomiting, fever, and other atypical symptoms. 2. Chronic: manifesting symptoms associated with cirrhosis. 3. The source or route of infection can be identified.^392^	1. Abnormal levels of biochemical indicators related to liver function, such as ALT, AST, and ALP. 2. Testing for viral antibodies or viral load is conducted to determine the presence of HAV, HDV, and HEV IgM or IgG; HBsAg, HBeAg, HCV-Ab; Viral RNA of HBV, HCV, and HGV.^393^	The classification of hepatitis is based on etiological factors, disease duration, disease severity, and other relevant aspects.^392^
ALI	Following direct or indirect exposure to various risk factors for liver injury, there was a rapid deterioration in liver function within a span of two weeks, accompanied by associated clinical symptoms such as weakness, decreased appetite, nausea, vomiting, epigastric pain, jaundice, and others.^403^	1. Serum ALT, ALP, GGT, and TBil alterations serve as the primary laboratory indicators for diagnosing ALI. 2. Liver failure is diagnosed when the following criteria are met: 1) Presence of severe gastrointestinal symptoms; 2) Progressive deepening of jaundice (serum TBil ≥ 171 μmol/L or daily increase ≥ 17.1 μmol/L); 3) Manifestation of bleeding tendency with PTA ≤ 40% or INR ≥ 1.5; and 4) Development of hepatic encephalopathy (degree II or higher), along with other complications.^405^	The definition of mild ALI is typically characterized by 2 ULN ≤ ALT < 5 ULN, while moderate ALI is usually defined as 5 ULN ≤ ALT < 15 ULN. Severe ALI, on the other hand, is indicated by an INR ≥ 2.0, ALT ≥ 10 ULN, and TBiL ≥ 3.0 mg/dL.^405,407^
MASLD	Excessive alcohol consumption should be ruled out, along with other causes of fatty liver disease, in patients presenting at least one component of Metabolic Syndrome (BMI ≥ 24 kg/m,^2^ blood pressure ≥ 130/85 mmHg, diabetes mellitus). Additionally, serum TG levels should be checked for values ≥ 1.70 mmol/L and high-density lipoprotein levels for values ≤ 1 mmol/L.^421^	1. Biochemical analysis index: FIB-4 > 1.3, ALT > 40 U/L. 2. Ultrasonic examination, such as LSM ≥ 8 kPa, CAP > 248 dB/m. 3. Liver biopsy histological examination.^421,422^	The liver biopsy histology of patients with MASLD revealed the presence of hepatic steatosis ≥ 5%, accompanied by concurrent lobular inflammation and balloon-like degeneration. Based on the extent of fibrosis, it can be categorized into early MASH (F0-1), fibrotic MASH (F2-3), and MASH cirrhosis (F4).^422,423^
ALD	1. Prolonged or excessive alcohol consumption history. 2. Manifestations can range from being asymptomatic to presenting with symptoms such as right upper quadrant abdominal pain, anorexia, asthenia, unintended weight loss and jaundice. With disease progression, signs indicative of cirrhosis become evident. 3. Exclude alternative etiologies for hepatic injury.^434^	1. ALT, AST, GGT, and MCV levels exhibit elevation, while the ratio of AST/ALT > 1.5. 2. Liver ultrasonography, computed tomography (CT), magnetic resonance imaging (MRI), or transient elastography reveal characteristic manifestations indicative of fatty liver disease or liver fibrosis.^434,436^	Maddrey discriminant function, model of end-stage liver disease (MELD) score, Glasgow alcoholic hepatitis score (GAHS).^434^
Autoimmune liver disease	The symptoms are typically mild in nature. Common manifestations include fatigue, abdominal discomfort, pruritus, icterus, hepatomegaly, nausea, anorexia, and acholic stools. More severe complications may encompass decompensated cirrhosis symptoms such as ascites and hepatic encephalopathy.	1. Serological testing: PBC (AMA, AMA-M2, anti-GP210 Ab, anti-SP100 Ab), AIH (ANA, ASMA, anti-SLA/LP, anti-LKM-1 and anti-LC-1 Abs, IgG and/or gamma-globulin), PSC (AMA and IgG4 levels).^134,444^ 2. Elevated levels of serum aminotransferases, ALP and GGT were observed. 3. Histopathological examination of liver biopsy.^447^ 4. Imaging studies including CT, MRI, MRCP.^445^	1. Paris diagnostic criteria. 2. IAIHG simplifies the scoring system.
Genetic and rare liver diseases	1. General symptom: unexplained aberrant hepatic function, hepatomegaly, cholestasis, neurological manifestations or additional systemic symptoms, hepatic cirrhosis, liver failure. 2. Unique clinical manifestations, such as Kayser-Fleischer ring, emphysema and other signs.^193^	1. Laboratory tests (e.g. liver function test, serum ceruloplasmin, serum α1-antitrypsin). 2. Imaging examination, ultrasound (e.g. CT and MRI) 3. Liver biopsy histological examination. 4. Genetic analysis.^104,141,460^	The grading of patients with advanced cirrhosis or liver failure should be conducted in accordance with their condition.^100^
Cirrhosis	The progression from an initially asymptomatic or mildly symptomatic compensatory phase of cirrhosis to a decompensated phase with portal hypertension and impaired liver function is frequently accompanied by complications, such as esophageal varices, gastrointestinal bleeding, ascites, hepatic encephalopathy, and jaundice.^462^	1. Histological examination of liver biopsy. 2. Indirect markers of fibrosis: FIB-4 > 2.67; BARD > 3, APRI > 1.5, Forns > 6.9, NAS > 0.676. 3. LSM ≥ 8 kPa.^155^	The Child-Pugh classification was employed to assess the severity of liver cirrhosis in patients based on parameters including prothrombin time, ascites, serum bilirubin levels, serum albumin concentration, and hepatic encephalopathy.^462^
HCC	1. Risk factors such as viral hepatitis, fatty liver, alcoholic hepatitis, or aflatoxin exposure may contribute to the development of the condition. 2. In the early stage, there are typically no specific symptoms present. However, as the disease progresses, individuals may experience liver pain followed by an upper abdominal mass, weakness, wasting syndrome, abdominal distension, fever, bleeding tendency, lower limb edema and potential bone metastasis which could include tenderness.	1. Histological examination of liver biopsy. 2. Imaging techniques including ultrasound combined with dynamic enhanced CT and MRI scanning, as well as digital subtraction angiography. 3. Nuclear medicine imaging examinations such as PET/CT and SPECT/CT.^477,478^ 4. Elevated levels of AFP (>20 ng/mL), DCP (>40 mAU/mL) and AFP-L3 (>10%).^479^ 5. Diagnosis using circulating microRNA, circulating tumor cell, cfDNA, circulating tumor DNA, and free DNA methylation biomarker panels either individually or in combination.	1. Classification of Liver Cancer (BCLC) at Barcelona Clinic, 2. Hong Kong Liver Cancer Stage (HKLC) stage, 3. BALAD staging.^489^

*AFP* alpha-fetoprotein, *AIH* autoimmune hepatitis. *AMA* anti-mitochondrial antibodies, *ANA* antinuclear antibodies, *ALD* alcohol-associated liver disease, *ALP* alkaline phosphatase, *ALT* alanine transaminase, *APRI* AST to platelet ratio index, *ASMA* anti-smooth muscle antibody, *AST* aspartate aminotransferase, *BMI* body mass index, *CAP* controlled attenuation parameter, *cfDNA* cell free DNA, *DCP* des-gamma-carboxy prothrombin, *ALI* acute liver injury, *FIB-4* fibrosis-4 index, *GGT* gamma-glutamyl transferase, *HAV* hepatitis A, *HBeAg* hepatitis B e antigen, *HBsAg* hepatitis B surface antigen, *HBV* hepatitis B, *HCC* hepatocellular carcinoma, *HCV* hepatitis C, *HCV-Ab* antibody of hepatitis C, *HDV* hepatitis D, *HEV* hepatitis E, *HGV* hepatitis G, *IAIHG* International Autoimmune Hepatitis Group, *INR* international normalized ratio, *LSM* liver stiffness measurement, *MASH* metabolic dysfunction associated steatohepatitis, *MASLD* metabolic dysfunction associated steatotic liver disease, *MCV* mean corpuscular volume, *MRCP* magnetic resonance cholangiopancreatography, *NAS* NAFLD activity score, *PBC* primary biliary cholangitis, *PSC* primary sclerosing cholangitis, *PTA* prothrombin time activity, *TBil* total bilirubin, *TG* triglyceride, *ULN* upper limit of normal

The treatment approach for liver disease is comprehensive, encompassing etiological management, lifestyle modifications, pharmacotherapy, nutritional support, prevention and management of complications, regular monitoring, and health education. Irrespective of the underlying cause, liver transplantation may represent the sole efficacious intervention for advanced liver disease following cirrhosis or hepatic failure.

### Viral hepatitis

Due to the presence of multiple types of hepatitis viruses and the possibility of acute or chronic viral infections in patients, serological testing is necessary following a thorough history collection and physical examination.^392^ Hepatitis virus antigen and corresponding antibody tests, along with etiological tests such as viral RNA load assessments, serve as crucial diagnostic indicators for identifying viral hepatitis in individuals presenting related symptoms.^393,394^ The diagnosis of viral hepatitis can be established by considering the patient’s clinical manifestations, evidence of liver function impairment in laboratory tests, and results from auxiliary imaging examinations while excluding other potential diseases that may present similar symptoms.

Vaccination is the most effective means of preventing infection with hepatitis A, B, and D viruses.^395^ The recombinant HBV vaccine is both safe and highly efficacious, capable of being administered as a standalone immunization or in conjunction with other antigens utilized in infant immunization programs or alongside the hepatitis A virus vaccine. The treatment approach for viral hepatitis should be tailored to each individual patient’s condition, including factors such as virus type, liver function status, presence of complications, and other relevant considerations. For patients afflicted by chronic or severe forms of hepatitis, antiviral therapy may be considered to impede progression towards cirrhosis, liver failure, and hepatocellular carcinoma. Antiviral agents like lamivudine, entecavir, and tenofovir can be employed for treating CHB while DAAs such as sofosbuvir and harvoni can be used against hepatitis C infections.^396,397^ Currently, nucleos(t)ide analogues have demonstrated safety along with efficacy in inhibiting HBV replication; however, they rarely achieve clearance of HBsAg necessitating long-term administration to prevent recurrence. Therefore, various classes of DAAs and immunomodulatory therapies are currently under development aiming at achieving functional cure defined as persistent undetectable HBsAg levels along with absence of detectable HBV DNA after completion of limited duration treatment.^398,399^ It might eventually require combination therapy involving multiple drug classes to attain this objective. Detailed information of viral hepatitis managements can be found in updated AASLD and EASL clinical practice guidelines.^393,394,396,400–402^

### Acute liver injury

Ancillary examinations revealed deranged liver function tests further supporting the initial diagnosis of acute liver injury (ALI).^403^ Thorough medical history collection can aid in identifying potential risk factors for ALI, such as recent initiation of medications or herbal/nutritional supplements intake, exposure to possible pathogens, travel history, and vaccination status.^404^ The definition of mild acute liver injury typically includes an ALT level between 2 and 5 times the upper limit of normal (ULN). Moderate ALI is usually defined as an ALT level between 5 times and 15 times the ULN. Severe ALI requires meeting specific criteria, including an international normalized ratio (INR) of ≥2.0, ALT levels of ≥10 ULN, and total bilirubin (TBiL) levels of ≥3.0 mg/dL without hepatic encephalopathy.^405^ An increase in the INR indicates a poor prognosis for patients with severe ALI.^406^ Studies have demonstrated that apart from etiology, bilirubin levels, INR values, and duration of jaundice are effective predictors for poor prognosis in ALI patients with specific thresholds such as duration of jaundice >3 days, TBil>51 μmol/L, and INR > 1.7.^407^

**Fig. 6:**
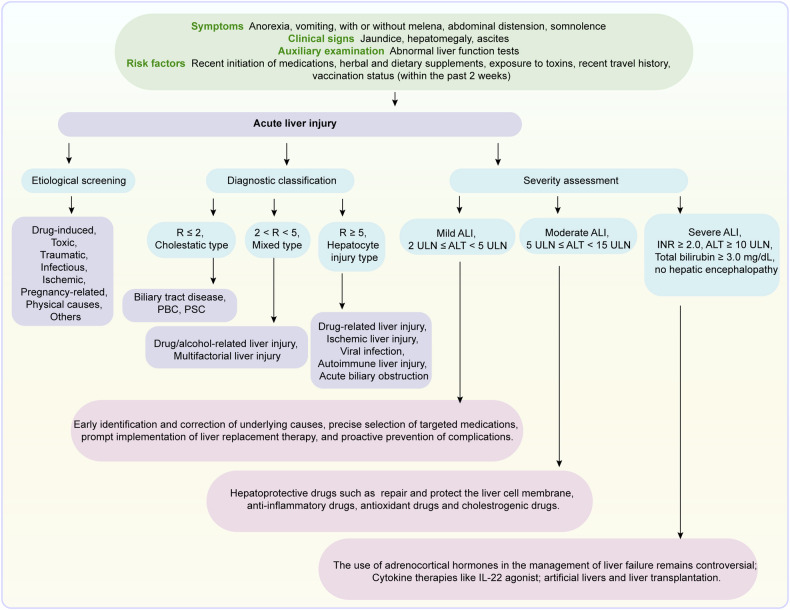
Comprehensive evaluation and therapeutic protocols for acute liver injury. The general diagnostic procedures for acute liver injury encompass etiological screening, identification of liver injury patterns, and assessment of severity. Based on the extent of hepatic damage, appropriate treatment modalities are employed, including causative factor elimination, supportive care administration, utilization of hepatoprotective agents, artificial liver support and liver transplantation. R = (ALT/ULN)/(ALP/ULN). This figure was generated with Adobe Illustrator

The fundamental principles of ALI treatment encompass early identification and correction of reversible causes, judicious selection of medications, timely implementation of liver replacement therapy, and proactive prevention and management of complications.^408^ Patients with abnormal liver function who do not yet meet the criteria for ALI should be closely monitored to promptly remove pathogenic factors in order to prevent liver damage or failure.^409^ For patients with rapid disease progression or existing liver damage, drug therapy should be considered based on active monitoring and etiological treatment. Currently available hepatoprotective drugs can generally be categorized into agents that repair and protect the liver cell membrane, anti-inflammatory drugs, antioxidant drugs (e.g. glutathione), and cholestrogenic drugs (e.g. ursodeoxycholic acid, UDCA).^410,411^ Presently, there is a lack of specific medications and approaches for treating advanced ALF. Thus, emphasis should be placed on symptomatic treatment while actively preventing complications. The use of adrenocortical hormones in the management of liver failure remains controversial; comprehensive consideration must be given to etiology and patient monitoring indices before making a decision.^412,413^ Cytokine therapies are under investigation. For example, An open-label, dose-escalation study utilizing IL-22 agonist F-652 to treat sAH has yielded promising results as anticipated, thereby offering a potential effective treatment strategy for further reducing the case fatality rate.^414,415^ Guidelines related to artificial livers and liver transplantation can serve as references for their respective treatments.^416,417^ Detailed information of acute liver failure and acute-on-chronic liver failure managements can be found in updated AASLD and EASL clinical practice guidelines (Fig. 6).^418–420^

### MASLD

The diagnosis of MASLD is based on three criteria: (1) imaging diagnosis of hepatic steatosis and/or liver biopsy findings of ≥ 5% hepatocyte ballooning degeneration; (2) presence of one or more metabolic syndrome score components; (3) exclusion of other causes that may contribute to hepatic steatosis.^421^ Ultrasound imaging is the preferred modality for diagnosing hepatic steatosis and monitoring hepatocellular carcinoma.^422^ Liver stiffness measurement obtained through shear wave elastography can be utilized for non-invasive assessment of hepatic steatosis and fibrosis in patients with chronic liver disease. In addition to meeting the diagnostic criteria for MASLD, the presence of ≥ 5% hepatocyte ballooning degeneration combined with lobular inflammation and/or portal inflammation can lead to a diagnosis of MASH. Given the associated risks, a liver biopsy is typically reserved for cases where MASH is suspected but cannot be confirmed by other means. Currently, clinicians make comprehensive judgments based on individual patient circumstances to select appropriate diagnostic methods.^423^

The management of MASLD necessitates a multidisciplinary approach, encompassing strategies such as weight and waist circumference reduction, enhancement of insulin sensitivity, prevention of metabolic syndrome and T2DM, mitigation of MASH, and reversal of fibrosis. Dietary modification and increased physical activity through health education serve as the fundamental pillars in the treatment regimen for MASLD. Greater weight loss in overweight/obese individuals confers additional benefits on metabolic cardiovascular health and liver function. A gradual weight reduction ranging from 3% to 5% within one year can reverse hepatic steatosis; a weight loss between 7% to 10% can alleviate MASH; more than 10% weight loss can lead to fibrosis regression; while a substantial decrease by 15% even improves T2DM symptoms.^424^ Unhealthy habits such as irregular eating patterns, soft drink consumption, smoking tobacco products, or alcohol use should be avoided alongside sedentary behavior and physical inactivity.^425^

**Fig. 7:**
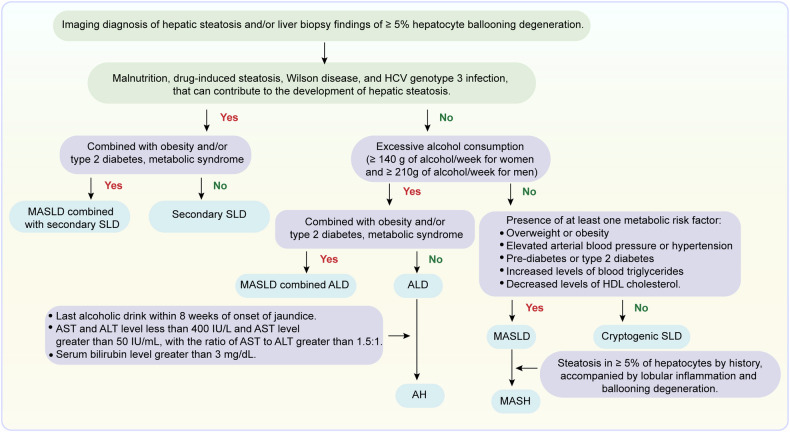
Etiological diagnostic approach for steatotic liver disease. The diagnosis of steatotic liver disease necessitates the integration of medical history (hypertension, T2DM, viral hepatitis, etc.), lifestyle styles (such as alcohol consumption), physical examination (body mass index, blood pressure, etc.), laboratory tests (triglycerides, glycated hemoglobin, ALT, AST, etc.), and pathological examination. This approach is based on expert opinion of the authors and evidence from published data. This figure was generated with Adobe Illustrator

Combined presence of metabolic cardiovascular risk factors and liver injury necessitates appropriate pharmacological intervention. Patients with MASLD and a BMI ≥ 28 kg/m^2^ may benefit from weight loss medications, while hypoglycemic drugs for weight reduction should be prioritized in the treatment of type 2 diabetes.^421,426^ In managing diabetic patients with MASLD, preference should be given to drugs such as metformin, pioglitazone, SGLT-2 inhibitors, GLP-1 receptor agonists, and other agents that have potential hepatoprotective effects.^427^ Statins are the primary choice for pharmacotherapy of arteriosclerotic lipid disorders in MASLD patients; however, caution or discontinuation is advised when using statins in individuals with severe liver diseases like decompensated cirrhosis.^428^ ACE inhibitors or ARBs are recommended as first-line therapy for hypertension in MASLD patients, whereas non-selective β-blockers can be used concomitantly if clinically significant portal hypertension is present.^429^ As an agonist of the thyroid hormone receptor-β (THR-β), resmetirom has recently gained FDA approval for the treatment of adult patients with MASH and liver fibrosis.^430^ For non-cirrhotic MASLD patients who meet the criteria for metabolic surgery aimed at weight loss, options such as gastric bypass surgery, sleeve gastrectomy, duodenal transposition, or adjustable gastric banding may be considered to address MASH and fibrosis.^431^ Liver transplantation could be an option for individuals with decompensated cirrhosis resulting from MASH complications or acute-on-chronic liver failure (ACLF) as well as those diagnosed with HCC.^432^ Detailed information of MASLD managements can be found in updated AASLD and EASL clinical practice guidelines (Fig. 7).^421,433^

### ALD

ALD is diagnosed in patients who typically have a prolonged history of alcohol consumption (more than 12 months, >2 drinks in women and >3 drinks in men per day) or recent heavy alcohol use within the past two weeks. The patient’s clinical symptoms were nonspecific and accompanied by abnormal serum liver function tests. Notably, an elevated AST/ALT ratio (>1.5), GGT levels, and mean corpuscular volume (MCV) values are characteristic of ALD.^434^ These indicators can significantly decrease after cessation of alcohol intake and usually return to normal within four weeks (although GGT may take longer to normalize), which aids in diagnosis.^435^ Imaging studies are used for diagnosis while excluding hepatitis viruses, medications, toxic liver injury, AIH, and other conditions.^436^ Symptomatic alcoholic hepatitis is diagnosed in the presence of jaundice and the criteria proposed by the National Institute on Alcohol Abuse and Alcoholism: last alcoholic drink within 8 weeks of onset of jaundice; serum bilirubin level greater than 3 mg/dL; AST and ALT levels less than 400 IU/L, with the AST level being greater than 50 IU/mL and an AST to ALT ratio greater than 1.5:1; and the exclusion of other potential causes of liver disease, biliary obstruction, and HCC.^437^

The treatment principles of ALD include abstinence and nutritional support, reducing the severity of the disease, and providing symptomatic treatment for alcoholic cirrhosis and its complications.^438^ Complete abstinence from alcohol enhances prognosis, mitigates liver histological injury, decreases portal vein pressure, delays fibrosis progression, and improves survival at all stages of the disease. Baclofen may be administered orally to individuals facing challenges with active abstinence.^434^ Moreover, patients with ALD require adequate nutritional support, including a high-protein, low-fat diet based on alcohol abstinence. Additionally, attention should be given to vitamin supplementation.^439^ Due to the limited expression of IL-22 receptor on epithelial cells, such as hepatocytes, IL-22 could serve as a specific target for preventing hepatocyte death and promoting hepatocyte proliferation without affecting immune cells. A multicenter trial is currently underway to investigate the use of IL-22Fc in treating ACLF, including severe alcoholic hepatitis (sAH) (CTR20212657).^415^ Inflammation has been extensively studied as a therapeutic target for sAH treatment due to its significant role in the pathogenesis of alcoholic liver disease. Steroid therapy has been utilized since the 1970s and emerging data suggest that it improves short-term survival in some sAH patients without impacting long-term survival.^435^ Treatment for anti-hepatic fibrosis should be prioritized along with actively addressing complications related to alcoholic cirrhosis such as esophageal and gastric varices rupture bleeding, spontaneous bacterial peritonitis, hepatic encephalopathy, and HCC.^440^ Liver transplantation may be considered for patients with severe alcoholic cirrhosis; however, many transplant centers require patients to abstain from alcohol for six months before undergoing surgery.^441^ Detailed information of ALD managements can be found in updated AASLD and EASL clinical practice guidelines (Fig. 7).^439,442^

### AIH

Patients with AIH often exhibit mild to moderate elevation of ALT and AST. On the other hand, patients with PBC and PSC frequently present elevated serum ALP and GGT levels, along with increased bilirubin levels in advanced stages.^134^ Serum autoantibodies such as anti-nuclear antibody (ANA) and anti-smooth muscle antibody (ASMA) can be detected in AIH patients, accompanied by elevated IgG levels.^443^ In 90-95% of PBC patients, serum antibodies against mitochondria (AMA) and elevated IgM levels can be found.^444^ Ultrasound, CT scan, MRI, endoscopic retrograde cholangiopancreatography (ERCP), and magnetic resonance cholangiopancreatography (MRCP) can be utilized to exclude biliary diseases like tumors or stones affecting the hepatobiliary system.^136^ ERCP is considered the “gold standard” for diagnosing PSC; however, MRCP is preferred due to its non-invasive nature when diagnosing this condition initially.^445,446^ Liver histological biopsy serves as a means to differentiate between causes of liver injury and evaluate tissue damage.^447^ Liver histological biopsy serves as a means to differentiate between causes of liver injury and evaluate tissue damage.^447^ The Paris diagnostic criteria and the AIH simplified diagnostic system are widely utilized for diagnosing AIH and its overlap with PBC. Among these, the Paris criteria stand out as the most common and effective tool for diagnosing AIH-PBC overlap syndrome.^448^ In 2008, the International Autoimmune Hepatitis Group introduced a simplified diagnostic scoring system for AIH, which proves valuable in identifying patients with AIH-PBC requiring corticosteroid treatment.^449^

The treatment and prognosis of different autoimmune liver diseases vary significantly. Immunosuppressive therapy is the primary approach for managing AIH, with a combination of prednisolone and azathioprine being the preferred treatment for AIH patients.^450^ In cases of poor response, alternative immunosuppressive agents can be considered. UDCA is the first-line option for PBC treatment, while additional medications such as bate drugs, budesonide, and obeticholic acid may be added if necessary.^451^ Currently, there are no drugs available to alleviate liver damage caused by PSC. Therefore, the focus lies in controlling complications and monitoring liver damage. UDCA can improve liver biochemical markers, reduce liver fibrosis severity, and enhance imaging findings related to biliary tract involvement.^452^ Glucocorticoids are favored for inducing remission in IgG4-associated hepatobiliary diseases.^453^ Patients who seek early treatment for AIH generally exhibit better treatment responses and prognoses comparable to those of healthy individuals in the long term. Conversely, patients who delay seeking treatment or do not respond well to therapy have an increased risk of developing cirrhosis and liver failure. Liver transplantation remains the sole effective intervention for end-stage liver disease.^454^ Detailed information of AIH managements can be found in updated AASLD and EASL clinical practice guidelines.^454,455^

### Genetic and rare liver diseases

Inherited liver diseases exhibit overlapping clinical manifestations, often requiring multiple clinical or pathological features for diagnosis.^100^ Genetic testing is the most crucial tool in diagnosing hereditary liver diseases. However, due to the complexity and diversity of genetic mutations in genetic liver disease, gene analysis and diagnosis remain challenging due to factors such as high cost, poor detection sensitivity, and the close relationship between heredity and acquired environment.^456^ Some rare or inherited liver diseases have unique clinical manifestations that aid in their diagnosis; for example, the combination of Kayser-Fleischer rings and a low serum ceruloplasmin (<0.1 g/L) level are prominent features of WD while decreased serum α1-antitrypsin levels with pulmonary damage such as emphysema suggest a1 antitrypsin deficiency.^193^ Notably, these features are not always reliable, and additional tests, such as high-quality imaging tests (e.g., MRI) or liver tissue biopsies, can also assist in making a definitive diagnosis.^457^

Several treatment options are available to alleviate symptoms and maintain optimal liver function for inherited liver disease. Dietary modifications may be necessary, such as adhering to a low-copper diet in cases of WD, and avoiding foods rich in copper like animal organs, dried fruits, and mushrooms.^458^ Symptomatic treatment often involves the use of medications that facilitate copper excretion or inhibit its absorption.^459^ Additionally, patients with liver damage can benefit from appropriate hepatoprotective therapy. For those experiencing neuropsychiatric symptoms, consultation with a neurologist is recommended for tailored management strategies. Itch relief can also be achieved through pharmacological interventions. In rare instances where hereditary liver diseases lead to ALF or decompensated cirrhosis unresponsive to conventional treatments or intolerant reactions occur, consideration should be given to liver transplantation.^460^ Detailed information of WD managements can be found in updated AASLD and EASL clinical practice guidelines.^100,461^

### Liver cirrhosis

The diagnosis of liver cirrhosis should be comprehensive, taking into account clinical manifestations of hepatic hypofunction and portal hypertension, as well as imaging and endoscopy findings, and laboratory results. Liver biopsy is recommended for patients with diagnostic difficulties, while etiological screening should be conducted whenever possible.^462^ Typical features observed in abdominal ultrasound, CT scans, and MRI images of cirrhosis include changes in liver volume (early enlargement followed by late contraction), abnormal ratio between the left and right lobes (shrinkage of the right lobe with enlargement of the left lobe and caudate lobe), irregular or jagged liver contour, widening of liver clefts, uneven liver echo or density signal distribution, dilation of the portal vein, and collateral circulation expansion.^155^ Transient elastography-derived liver stiffness measurement (LSM) demonstrates a strong ability to evaluate significant liver fibrosis and cirrhosis but exhibits poor accuracy in assessing mild stages of fibrosis. Magnetic resonance elastography (MRE) offers high diagnostic accuracy along with good stability and efficiency for staging liver fibrosis because it is less influenced by factors such as obesity or ascites; however, it requires relatively more time for examination and is expensive.^463^ Serological indicators such as aspartate aminotransferase-platelet ratio index (APRI) and fibrosis-4 index (FIB-4) exhibit low sensitivity and specificity in diagnosing cirrhosis. Moreover, the critical value used to determine liver fibrosis/cirrhosis can also be affected by etiology among other factors.^464^ According to the presence of esophageal and gastric varices, hemorrhage, ascites, hepatic encephalopathy, and jaundice, cirrhosis is classified into six stages. Stage 1 does not exhibit varicose veins or any other complications; it is further divided into stages 1a and 1b based on whether the hepatic venous pressure gradient (HVPG) is ≥10 mmHg. Varicose veins appear in stage 2 but without EGVB (esophagogastric variceal bleeding) or ascites. EGVB occurs in stage 3 but without decompensation such as ascites or hepatic encephalopathy. Stage 4 includes various forms of decompensation except for EGVB, including ascites, overt hepatic encephalopathy, overt bacterial infection, and non-obstructive jaundice. Stage 5 presents two types of decompensations while stage 6 is characterized by recurrent infection, dysfunction of extrahepatic organs, ACLF, refractory ascites, persistent hepatic encephalopathy or jaundice.^462,465^

The most crucial treatment for cirrhosis is the removal of its underlying cause. Etiological control, particularly antiviral therapy in patients with hepatitis B/C, as well as abstinence in those with alcoholic cirrhosis, can potentially reverse liver fibrosis and cirrhosis or restore compensatory stage in decompensated cirrhosis patients.^466^ In cases where malnutrition complicates cirrhosis, it is recommended to consume 25–35 kcal/kg/d energy intake, 1.0–1.5 g/kg/d protein intake, increase meal frequency, add extra meals at night, and adequately supplement dietary fiber, vitamins, and trace elements. Patients with ascitic cirrhosis should moderately restrict sodium intake (85–120 mmol/d or equivalent to 5.0–6.9 g/d salt) while avoiding extreme sodium restriction (<40 mmol/d). Unless moderate to severe dilutive hyponatremia (blood sodium <125 mmol/L) is present, water intake does not generally need to be restricted in patients with ascites due to cirrhosis.^467,468^ Diuretics are considered the first-line treatment for ascites in cirrhotic patients; spironolactone alone or combined with furosemide or torasemide can be used. Grade 2 or 3 ascites that are unresponsive to conventional diuretics may be managed with the administration of tolvaptan.^469^ Massive paracentesis is a commonly employed intervention for refractory ascites, and albumin infusion should be utilized to optimize intravascular volume expansion. Teripressin represents an efficacious pharmacological option for the treatment of refractory ascites.^470^ Transjugular intrahepatic portosystemic shunt (TIPS) should be considered in cases where therapy for massive ascites proves ineffective.^471^ Liver transplantation serves as the definitive therapeutic approach for decompensated cirrhosis and warrants evaluation when patients develop esophageal variceal bleeding, refractory ascites, hepatorenal syndrome, hepato-pulmonary syndrome, recurrent hepatic encephalopathy, ACLF, or HCC.^472^ Detailed information of cirrhosis managements can be found in updated AASLD and EASL clinical practice guidelines.^462,473–476^

### HCC

Traditionally, the diagnosis of HCC has been primarily based on cytology or histology. However, with advancements in staged perfusion angiography during CT and MRI cross-sectional imaging, HCC can now be reliably diagnosed radiologically in cirrhotic patients under surveillance without the need for biopsy.^477^ Abdominal ultrasound is currently considered the most recommended method for monitoring HCC.^477,478^ Although serum alpha-fetoprotein (AFP) alone lacks sensitivity and specificity to serve as an independent monitoring test, its combination with ultrasound significantly improves early detection sensitivity for HCC.^479^ Various integrated imaging and blood-based strategies have been proposed to enhance early detection of HCC; nevertheless, most of these approaches have only been evaluated through case-control studies and require prospective validation.^480^

**Fig. 8:**
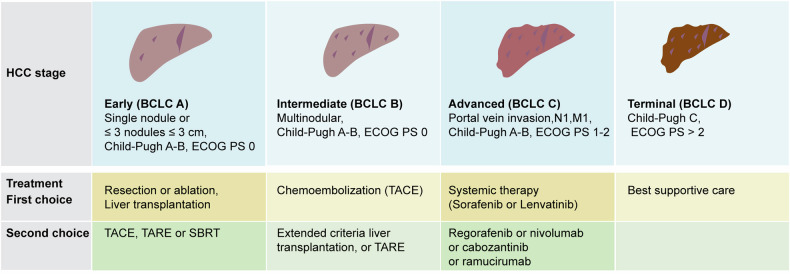
Strategy for HCC treatment with BCLC staging system. The Barcelona Clinic Liver Cancer (BCLC) staging system categorizes hepatocellular carcinoma into five stages (0/A to D) with varying prognostic significance. The authors have provided a concise summary of the recommended first and second treatment options based on the BCLC stage. ECOG PS, Eastern Cooperative Oncology Group performance status; SBRT, stereotactic body radiation therapy. This figure was generated with Adobe Illustrator

Over the past two decades, the Barcelona Clinic Liver Cancer (BCLC) staging system has gained recognition from the majority of professional societies.^481^ However, managing HCC involves a complex decision-making process, and the availability of treatment options varies significantly among medical centers across different countries. Consequently, effective HCC management necessitates multidisciplinary collaboration to devise tailored strategies that cater to each patient’s unique circumstances in order to achieve optimal outcomes.^482^ Surgical treatment options for HCC include surgical resection and liver transplantation, both considered potentially curative treatments. However, it is important to note that nearly 70% of patients experience recurrent HCC after resection.^483^ Liver transplantation stands out as the most definitive treatment option for early-stage HCC since it allows removal not only of the tumor but also an unhealthy liver with limited functional capacity. A retrospective multicenter study involving 187 HCC patients revealed that 58% underwent successful downstaging followed by liver transplantation with a 5-year survival rate reaching 80%.^484^ Percutaneous local ablation is a potentially curative treatment modality that can be employed in patients with early HCC. The two most commonly utilized techniques are radiofrequency ablation (RFA) and microwave ablation (MWA).^485^ MWA demonstrates enhanced efficacy for larger tumors measuring 3-4 cm, and requires less procedural time compared to RFA.^486^ Transarterial chemoembolization (TACE) is a highly effective treatment modality for patients with intermediate-stage HCC. Transarterial radiation embolization (TARE) represents an alternative local regional therapy approach, which can be employed as the primary therapeutic intervention for unresectable HCC cases.^487^ Unlike TACE, TARE involves intratumoral brachytherapy techniques and exerts minimal embolic effects on hepatic artery distribution, making it suitable even for patients presenting portal vein thrombosis or tumor invasion (Fig. 8).^488^

The treatment strategy for HCC has been significantly revolutionized by the introduction of systemic pharmacological therapy worldwide.^489^ Sorafenib, lenvatinib, cabotinib, ramociumab, and other drugs have successively gained approval for HCC treatment.^490–492^ However, due to the heterogeneity and complexity of HCC pathogenesis, precise treatment for this disease is still under investigation. Concurrently, immune checkpoint inhibitors have emerged as a promising therapeutic option for advanced HCC.^493^ Various immunotherapies including checkpoint inhibitor combined targeted therapy, checkpoint inhibitor combination therapy, and non-checkpoint inhibitor immunotherapy (such as immune cell adoptive therapy) have shown significant efficacy.^494,495^ In conclusion, active research is still required in this field and a combination of treatment modalities may enhance therapeutic options for patients. Detailed information of HCC managements can be found in updated AASLD and EASL clinical practice guidelines.^482,496^

## Clinical research progress

### Acute liver disease-viral hepatitis

Acute viral hepatitis, resulting from viruses such as HAV-HEV, primarily benefits from prevention via inactivated vaccines.^497^ Currently, there is no specific antiviral for HAV, but clinical studies suggest benefits from steroids and IFN-β in improving outcomes.^498^ For severe acute HBV, early administration of lamivudine and entecavir has been shown to improve patient conditions and reduce progression to chronic hepatitis.^499,500^ Therapeutic strategies such as ledipasvir/sofosbuvir have been effective in treating HIV and HBV co-infections by shortening treatment durations.^501^ Meanwhile, grazoprevir combined with elbasvir shows promise in acute HCV, particularly for genotypes 1 or 4.^502^ For HEV, ribavirin has been effective in reducing viral load (Table 3).^503^ Research on treatment for acute HDV is limited and warrants further exploration. Additional studies reveal that other virus like adenovirus, cytomegalovirus (CMV), Epstein-Barr virus (EBV), and TT virus can also cause acute viral hepatitis.^504,505^ Rare viral hepatitis are often overlooked due to their low incidence. These less common forms underline the need for heightened clinical awareness to prevent misdiagnosis and inappropriate treatment.

**Table 3:** Clinical studies on acute and chronic liver disease treatment

Disease category	Therapy	Clinical efficacy	Quality of evidence	Application stage	Ref.
*Acute liver disease*					
Acute HAV	Steroid	Enhance survive rate and recovery time	Cohort study	FDA approval	^498^
Acute HBV	Lamivudine Entecavir	Improve condition and prognosis Better than lamivudine	RCT Cohort study	FDA approval FDA approval	^499,500^
Acute HCV	Grazoprevir/elbasvir Ledipasvir/sofosbuvir	Improvement in acute HCV genotype 1 or 4 Improve clinical symptoms of HIV-HCV co-infection	Cohort study Cohort study	FDA approval FDA approval	^501,502^
Acute HEV	Ribavirin	Improvement in recovery time	Cohort study	FDA approval	^503^
DILI	NAC	Exhibit higher redox thiol response	Cohort study	FDA approval	^507^
ALD	Pentoxifylline Corticosteroids TNF-α inhibitors	Reduces short-term risk of death Reduces short-term risk of death Improves inflammation but increases the risk of infection	Meta-analysis Meta-analysis RCT	Phase 3 FDA approval FDA approval	^508–510^
*Chronic liver disease*					
Chronic HBV	Vebicorvir TAF PD-1 inhibitors ARC-520	Better viral suppression than NrtI Improved bone and renal safety without a loss of efficacy compared with TDF Restoration of HBsAg-specific B cells Decrease HBsAg and HBV DNA levels	RCT RCT Cohort study RCT	FDA approval FDA approval Phase 3 Phase 2	^512–515^
Chronic HCV	Glecaprevir/pibrentasvir	Highly efficacious and well tolerated in patients with HCV genotype 1	Cohort study	FDA approval	^517^
Chronic HDV	Bulevirtide/TAF Peginterferon alfa-2a/TDF	Reduces HDV RNA but requires long-term use Combination therapy does not enhance efficacy	RCT RCT	Phase 3 Phase 3	^518,519^
ALD	Corticosteroids/nutritional support	Hospitalized patients with hepatitis have a benefit in mortality with adequate oral intake	RCT	FDA approval	^523^
MASLD	Saroglitazar Resmetirom	Improve liver function and reduce dyslipidemia Reduce inflammation, improve liver lipid accumulation, and reduce liver fibrosis	RCT RCT	Phase 2 FDA approval	^430,527^
AIH	Budesonide	Less effective than prednisone, but with fewer side effects	Cohort study	FDA approval	^529^
PSC	Obeticholic acid	Improvement in liver damage observed during 2-year monitoring	RCT	FDA approval	^532^
*End-stage liver disease*					
Cirrhosis	Pegbelfermin Aldafermin MSC	Improve MASH-related fibrosis and compensated cirrhosis Improve liver fibrosis in compensated cirrhosis patients Enhances long-term survival and liver function in patients with HBV-related decompensated cirrhosis	RCT RCT RCT	Phase 3 Phase 2	^534–536^
Liver failure	HRX215	Promote liver regeneration and prevent liver failure Reduce the mortality rate in these patients	Cohort study	Phase 1	^538^
HCC	Camrelizumab/rivoceranib Sintilimab Lenvatinib/TACE Atezolizumab/bevacizumab	Better progression-free survival and overall survival for unresectable HCC Prolong recurrence-free survival than active surveillance Prolong overall survival for advanced HCC Prolong recurrence-free survival	RCT RCT RCT RCT	Phase 3 Phase 2 Phase 3 Phase 3	^541–543,545^

*AIH* autoimmune hepatitis, *ALD* alcohol-associated liver disease, *DILI* drug-induced liver injury, *HAV* hepatitis A virus infection, *HBV* hepatitis B virus infection, *HCV*, hepatitis C virus infection, *HCC* hepatocellular carcinoma, *HDV* hepatitis D virus infection, *HEV* hepatitis E virus infection, *MASLD* metabolic dysfunction-associated steatotic liver disease, *MSC* mesenchymal stem cell, *NAC* N-acetylcysteine, *NrtI* nucleotide reverse transcriptase inhibitor, *PD-1* programmed death-1, *RCT* randomized controlled trial, *TACE* transarterial chemoembolization, *TAF* tenofovir alafenamide fumarate, *TDF* tenofovir disoproxil fumarate, *TNF-α* tumor necrosis factor-alpha

### Acute liver disease-DILI

Acute DILI, often caused by substances such as acetaminophen, antibiotics, or anti-inflammatory drugs, usually resolves with decreasing liver enzyme levels within days to weeks, with fewer than 10% of cases progressing to chronic liver damage.^506^ Acetaminophen overdose is primarily treated with N-acetylcysteine (NAC).^507^ Investigating additional treatments that can protect liver function during both the early and late stages of DILI is critical for minimizing long-term damage.

### Acute liver disease-ALD

Acute ALD remains challenging, and therapies such as pentoxifylline and corticosteroids shown to decrease short-term, but not medium-term, mortality.^508^ Although corticosteroids may reduce short-term mortality, their long-term safety profile is concerning due to the risk of severe infections.^509^ TNF-α inhibitors, such as infliximab and etanercept, reduce inflammation but increase infection risks.^510^ There is emerging interest in the role of intestinal microbes in ALD, though clinical validations are still preliminary.^511^

### Chronic liver disease-viral hepatitis

Innovations in CHB treatments include vebicorvir, a core inhibitor that has shown superior efficacy compared to traditional nucleoside reverse transcriptase inhibitors.^512^ Tenofovir alafenamide is used for multidrug-resistant HBV strains, improving long-term outcomes.^513^ PD-1 inhibitors and RNA interference therapies like ARC-520 are under investigation for their potential to enhance immune responses and reduce viral load in HBV patients.^514,515^ Treatments for chronic HCV have evolved with the development of DAAs, reducing concerns about VZV reactivation.^516^ Glecaprevir and pibrentasvir have shown improved responses in HCV patients who failed prior DAA therapies.^517^ Bulevirtide combined with tenofovir disoproxil fumarate offers new hope for HDV patients, although more studies are needed to confirm these findings.^518,519^ Overall, these advancements represent significant progress in the treatment of liver diseases, but ongoing research is crucial to optimize safety and long-term efficacy of these therapies.

### Chronic liver disease-ALD

No FDA-approved medications currently exist specifically for ALD. Research indicates that alcohol consumption disrupts the intestinal microbiota, sometimes leading to an overgrowth of *Candida albicans*. This alteration suggests that probiotics could help mitigate ALD by modulating intestinal flora.^520,521^ Inflammation is a critical factor in ALD pathogenesis, hence steroids are used to manage symptoms and improve short-term survival rates, although their long-term efficacy is still not well-established.^522^ Corticosteroids treatments seem to be effective, which needs adequate nutritional intake throughout the treating duration.^523^ Efforts to target inflammation with cytokines such as IL-1 and TNF-α have been explored, but results, including attempts to combine IL-1 receptor antagonists with pentoxifylline and zinc, have not shown superior outcomes compared to corticosteroids alone.^524,525^ Future research is essential to develop more targeted therapies for ALD.

### Chronic liver disease-MASLD

With rising global obesity rates, MASLD prevalence is expected to increase.^526^ In a significant development, in 2020, saroglitazar was approved by India’s Drug Administration as the first medication specifically for MASLD, demonstrating effectiveness in reducing ALT level, liver fat content, insulin resistance, and atherogenic dyslipidemia.^527^ Furthermore, in March 2024, Resmetirom became the first FDA-approved drug for treating non-cirrhotic NASH with moderate to advanced liver fibrosis in adults, marking a major milestone in MASLD treatment.^528^ Despite these advancements, there remains a substantial need for more precise non-invasive diagnostic techniques and effective treatments.

### Chronic liver disease-autoimmune liver diseases

Recent advances in AIH research have improved our understanding of its causes, diagnosis, and treatment. Glucocorticoids and immunosuppressants remain treatment mainstays,^529^ with studies showing that combining mycophenolate mofetil (MMF) with prednisolone can lead to better outcomes and fewer side effects.^530^ Future directions include advancing personalized treatment strategies to enhance patient quality of life and prognosis. Cholestatic liver diseases such as PBC and PSC are primarily managed with UDCA.^531^ Emerging treatments, such as obeticholic acid, are undergoing evaluation for their efficacy in these diseases.^532^ For PSC, liver transplantation remains a definitive but severe option, underscoring the ongoing need for research into pharmacological interventions that could slow disease progression.

### End-stage liver disease-cirrhosis

As a critical aspect of end-stage liver disease, cirrhosis has undergone significant advancements in both diagnosis and management. The integration of non-invasive tests like transient elastography and serum biomarkers (e.g., FibroTest) into clinical practice has greatly improved the early detection of liver fibrosis and cirrhosis, reducing reliance on invasive biopsy procedures.^533^ Recent studies have highlighted the potential of pegbelfermin and aldafermin in ameliorating liver fibrosis associated with MASH and compensated cirrhosis.^534,535^ Additionally, anti-fibrotic medications and treatments aimed at enhancing liver microcirculation are showing promising results in clinical trials. Research into the therapeutic application of mesenchymal stem cells for decompensated cirrhosis has also yielded positive outcomes, although more extensive studies are required to confirm these findings.^536,537^ Liver transplantation continues to be the sole curative treatment for cirrhosis, underscoring the ongoing need for the development of more effective therapies.

### End-stage liver disease-liver failure

Liver failure represents the most severe manifestation of end-stage liver disease, where managing both acute and chronic forms remains a formidable challenge. Recent advancements include the development of the MKK4 inhibitor HRX215, which has been shown to promote liver regeneration and prevent liver failure.^538^ Extracorporeal liver support devices have also shown promise in improving patient outcomes in acute and chronic liver failure, potentially reducing mortality rates.^539^ Innovations in regenerative medicine, including stem cell therapies and liver bioengineering, are being explored as novel treatment avenues.^540^ Moreover, improvements in liver transplantation techniques and refinement of immunosuppressive treatments are crucial for enhancing patient survival and quality of life.

### End-stage liver disease-HCC

There has been notable progress in clinical research on HCC, yielding several pioneering treatments. Research comparing PD-1 antibody carrelizumab with VEGFR2-targeted TKI rivoceranib has indicated better progression-free and overall survival rates in patients with unresectable HCC compared to standard treatments like sorafenib.^541^ In high-risk post-resection HCC, the PD-1 inhibitor sintilimab shows potential in reducing tumor recurrence.^542^ The LAUNCH trial revealed that combining lenvatinib with TACE significantly enhances clinical outcomes in patients with advanced HCC.^543^ Furthermore, a phase 1/2 trial exploring a personalized neoantigen vaccine (PTCV) combined with pembrolizumab has demonstrated promising immune responses and preliminary efficacy in advanced HCC cases.^544^ For patients at high risk of HCC recurrence after postoperative ablation, atezolizumab combined with bevacizumab improves recurrence-free survival.^545^ These emerging therapies offer new hopes and strategies in the fight against HCC, potentially transforming the therapeutic landscape.

## Conclusions and perspectives

The etiology of liver diseases is continually evolving. With the global rise in obesity and T2DM, MASLD poses a growing health threat worldwide. The administration of vaccination and antiviral medications has significantly decreased the incidence of viral hepatitis in the Americas and Europe; however, the prevalence of MASLD, ALD, and DILI is rising. Despite advancements in vaccinations and antiviral medications that effectively prevent and combat viral infections, chronic hepatitis B and C remain prevalent, particularly in low-income countries lacking adequate medical resources. Additionally, the incidence of ALD is increasing, especially among younger populations. Despite heightened public health efforts, liver diseases significantly contribute to the global disease burden.^546^

The diagnosis of liver diseases primarily depends on liver biopsy, an invasive technique unsuitable for broad screening. The absence of reliable biomarkers for the precise diagnosis and staging of specific liver diseases poses a significant challenge.^547^ As such, developing novel non-invasive biomarkers and methods is crucial for the early detection of asymptomatic liver diseases. Such advancements could help identify high-risk individuals sooner, allowing for early interventions to halt disease progression.

Although there has been notable progress in understanding liver disease pathogenesis through advanced technologies, therapeutic options approved by the FDA remain limited, and existing medical interventions often provide minimal long-term survival benefits. The complexity of liver disease pathophysiology and the substantial heterogeneity in disease phenotypes mean that current mouse models do not adequately mimic the full spectrum of human liver diseases, including ALD and MASLD. Significant disparities exist between mouse models and human conditions in terms of pathophysiology and treatment outcomes, as numerous clinical trials have shown that drugs effective in mouse models fail to offer clinical benefits in humans.^548^ The properties of chemical absorption, distribution, metabolism, excretion, and toxicity differ between species, resulting in drug doses that are beneficial and non-toxic in mice showing insufficient efficacy or causing side effects in humans. For example, galectin-3, which was demonstrated to reduce liver inflammation and fibrosis in murine MASH models, did not translate effectively to human patients.^549^ Belapectin, a galectin-3 inhibitor, failed to show efficacy in MASH patients with cirrhosis and portal hypertension in a phase 2b randomized trial, possibly due to inadequate treatment duration and dosage. Pharmacokinetic analysis revealed differences in the metabolism of belapectin between mice and humans.^550^ This discrepancy underscores the need for the development of more standardized mammalian models, such as pigs and chimpanzees, which might bridge these gaps and improve translational success.

Moving forward, our current understanding of the pathogenesis has provided valuable insights and directed ongoing research efforts aimed at liver disease treatment. However, a comprehensive understanding of critical signaling pathways and their interactions during liver disease progression is essential to advance therapeutic strategies and improve patient outcomes.

## Supplementary information

Smilarity check-part 1

Smilarity check-part 2
