# Supplementary material for: Liver diseases: epidemiology, causes, trends and predictions
Source: Signal Transduct Target Ther. 2025 Feb 5;10:33. doi: 10.1038/s41392-024-02072-z (PMC11794951; doi:10.1038/s41392-024-02072-z)
Supplement: Supplementary file 1 — Smilarity check-part 1 [file 41392_2024_2072_MOESM1_ESM.pdf]

20240930140919103813257768300953

WORD COUNT 7520

TIME SUBMITTED 30-SEP-2024 02:09PM

PAPER ID 112029126

Inter-cellular mechanisms

Cellular crosstalk is a crucial event that maintains liver homeostasis. When liver injury occurs, hepatocytes and non-parenchymal cells engage in pathological paracrine interactions. In this section, we describe the cellular crosstalk during liver diseases.

Hepatocytes

Hepatocytes, major parenchymal cells in the liver, perform diverse roles in lipid and glucose metabolism, detoxification and protein synthesis. During disease states, hepatocytes face direct assaults from viruses or metabolites but also respond to signals from neighboring cells. The injury imposed on hepatocytes may exacerbate cellular dysfunction and subsequent death.[318] Liver macrophages serve as primary reservoirs for inflammatory cytokines such as IL-1β and TNF-α, which contribute to hepatocyte death.[319] It has been well-established that IL-1 drives hepatocyte inflammation and apoptosis via interacting with its receptor and downstream effectors. In contrast, hepatocyte-specific depletion of IL-1 receptor rescues hepatocyte apoptosis by blocking JNK/NF-κB signaling during acute liver injury.[320] Inflammasomes released from macrophages also contribute to hepatocyte pyroptosis and liver inflammation in mouse model of MASLD.[320] In addition, Wnt2 protein derived from LSECs was found to regulate cholesterol and bile acid homeostasis in hepatocytes.[321] However, silencing the expression of LSEC-specific Wnt2 disturbs hepatocyte metabolic profiles in both acute and chronic liver injury, indicating the essential role of LSECs in hepatocyte function.[322]

Cholangiocytes

Cholangiocytes are highly specialized epithelial cells forming the bile ducts and are essential for bile acid homeostasis. In chronic liver injury, such as cholangiopathies, a pathological feature known as a ductular reaction occurs, characterized by the proliferation of reactive ductular cells.[323] Ablation of β1-integrin in hepatocytes stimulates the ductular reaction, leading to cholangiocyte-derived hepatocyte regeneration during chronic liver diseases. These data indicate the potential network between hepatocytes and cholangiocytes.[324] Inflammatory and fibrotic secretions from immune cells are involved in cholangiocyte activation and biliary repair, which in turn, leads to increased inflammatory cell infiltration and persistent liver

impairment.[325,326]

LSECs

LSECs are highly differentiated endothelial cells lining the liver sinusoids. LSECs possess a unique phenotype with fenestrae, enabling substantial exchanges and cellular communications.[327] Although LSECs detect extrahepatic signals and help maintain liver homeostasis through angiocrine mechanisms, their function and architecture are regulated by other cell types. Inflammatory cell populations, such as $CCR2^+$ macrophages and $CXCR1^+$ neutrophils, which are recruited by injured LSECs in the early phase of liver damage, could in turn compromise LSEC endocytosis capacity and cause its fenestrae impairments.[328,329] Moreover, neutrophil adhesion attracts platelet recruitment, leading to the generation of sinusoidal microthrombi, which in turn induces sinusoidal dysfunction and vasoconstriction.[330] This pathological cascade elevates sinusoidal pressure, exacerbating liver fibrosis and portal hypertension. HSCs are crucial players to maintain LSEC phenotype. Bone morphogenetic protein 9 (BMP-9) has been identified to control vessel homeostasis.[331] Intriguingly, HSCs are the hepatic cell source of BMP-9, which emphasize the impact of HSC-derived BMP-9 on LSEC phenotype and function via targeting its receptor activin receptor-like kinase 1 (ALK-1).[332] Aberrant expression of BMP-9 or ALK-1 depletion in LSECs during diseased states results in impaired angiocrine function and LSEC architecture, underlining the effect of HSCs on LSEC physiological process.[333]

HSCs

Quiescent hepatic stellate cells (qHSCs) represent about 5% of liver resident cells and reside in the space of Disse. Following liver damage, qHSCs convert to an activated myofibroblast phenotype characterized by proliferation, contractility, and chemotaxis.[334] Fate tracing analysis has implicated that activated HSCs are the predominant source of ECM in liver diseases induced by toxic, fatty and cholestatic insults.[335,336] These activated HSCs migrate to the injured sites, where they proliferate and contribute to ECM production, thus participating in liver repair. Multiple mediators from other liver cell types contribute to HSCs activation. LSECs are responsible for the production of TGF-β and activation of HSC via the angiocrine pathway

during the early stage of liver injury.[337,338] Besides canonical TGF-β signaling, platelet-derived growth factor (PDGF) and IL-6 produced by capillarized LSECs also contribute to HSC activation by the JAK/STAT pathway.[339] Notably, macrophages are the major producers of TGF-β, leading to subsequent HSC activation during liver injury. Loss of TGF-β results in a significant decrease in ECM deposition.[340] NLRP3 inflammasome, induced by pyroptotic hepatocytes, also activates HSCs by releasing multiple cytokines in CCl_4-induced liver injury.[341] In summary, various liver cell types are responsible for HSC activation to induce liver fibrosis.

Macrophages

As the predominant immune cell type in the liver, macrophages play crucial roles in maintaining liver homeostasis and responding to diseases. Hepatic macrophages are composed by liver-resident Kupffer cells (KCs) and monocyte-derived macrophages (MoMFs), two heterogeneous subpopulations with distinct functions.[342] KCs primarily serve as the main source of hepatic macrophages in a healthy liver, responsible for sensing injury signals and clearing cellular debris.[343] Under inflammatory conditions, MoMFs infiltrate and become the major component of hepatic macrophages with the loss of KCs.[344] Macrophages play essential roles in liver inflammation and fibrosis via the production of inflammatory cytokines and chemokines and the activation of inflammasomes.[345] LSECs recruit MoMFs in a CCL2/CCR2-dependent manner during chronic liver injury. Specifically, by deleting LSEC-specific CCL2, infiltrating macrophage recruitment, liver inflammation, and fibrosis were reduced in CCl_4-induced mice.[328] In addition, transcriptome analysis and animal experiments reveal that hepatocytes are involved in the recruitment of MoMFs via the CCL2-CCR2 interaction in acute liver injury to facilitate necrotic lesion resolution.[346,347] Besides, mediators secreted by hepatocytes, such as IL-17, IL-1β and extracellular vesicles (EVs), mediate inflammatory macrophage infiltration and the development of ALD and MASLD.[348,349] However, certain inflammatory macrophages switch to an anti-inflammatory $LY6C^{low}$ phenotype during the progression of liver diseases, which plays an important role in ECM degradation via secretion of MMPs.[350] Moreover, targeting myeloid-derived RNF-41 has been shown to promote this phenotypic switch and subsequent ECM degradation, thus leading to fibrosis resolution.[351] While the data indicate the

dual roles of macrophages, further investigations are needed to elucidate the precise mechanisms of macrophage phenotype switch during liver inflammation and fibrosis.

Other immune cells

The T cell-mediated adaptive immune response plays central roles in antigen-driven liver diseases such as autoimmune hepatitis and chronic viral hepatitis.[352] Autoantigens in hepatocytes, such as formiminotransferase cyclodeaminase, and cytochrome P450 2D6, are presented in the naive $CD4^+$ T-helper lymphocytes. These Th0 cells subsequently differentiate into Th1 and Th2 cells, which are responsible for macrophage and B cell infiltration, respectively. This immune response cascade attacks hepatocytes, contributing to liver injury.[353,354] Similarly, the T cell response is also pivotal in the clearance of HBV and HCV. Effector T cells recognize HBV/HCV-infected hepatocytes and eliminate the virus through a combination of cytotoxic and non-cytotoxic pathways.[355,356]

In a healthy liver, neutrophils are typically absent, but their infiltration within liver sinusoids is noted during acute and chronic liver diseases.[357] Research has highlighted that LSEC-dependent neutrophils infiltration occurs through the secretion of CXCL chemokines.[358] Growing evidence has emphasized the significance of neutrophil in liver diseases, as they are thought to promote liver inflammation by releasing cytokines and chemokines, along with recruiting various other immune cells and potentially contributing to the formation of microthrombi.[359,360] Collectively, these findings indicate that immune cells play multifaceted roles in the progression of liver diseases, impacting both the immune response and inflammatory processes within the liver.

Liver-organ communication

Emerging evidence has shown that pathological changes at the molecular and cellular levels may not fully account for the pathogenesis of liver diseases, implying a potential role for inter-organ communications in their development. Recently, the gut-liver-brain and adipose-liver homeostasis has gained much attention.[361] In this section, we summarize the recent research on the importance of liver-organ interactions concerning metabolism, immune system, and nervous system.

Gut-liver-brain axis

Numerous preclinical and clinical studies have highlighted the role of abnormal gut microbiota and their metabolites in impairing intestinal barrier function, further contributing to liver diseases. Increased permeability is reported in mice fed a high-fat diet or with excessive ethanol intake.[362,363] Gut leakage leads to the delivery of pathogens and their metabolites to the liver via portal vein. In patients with MASH, gut microbiomes predominantly exhibit Gram-positive *Firmicutes*. However, there is a decrease in *Firmicutes* and an increase in Gram-negative *Proteobacteria* abundance as liver fibrosis develops.[364] Animal studies demonstrate that germ-free mice fed a high-fat diet show reduced liver steatosis and insulin resistance compared to wild-type mice on the same diet.[365] This beneficial effect disappears following fecal microbiota transplantation (FMT) from MASLD mice to germ-free mice, resulting in increased liver triglyceride content and inflammation.[366] These data indicate that dysregulated gut microbiota is essential for the progression of MASLD. Besides pathogenic bacteria, their metabolites also mediate liver diseases. For instance, short chain fatty acids (SCFAs) produced by gut bacteria have been linked to promoting hepatic *de novo* lipogenesis and glucose production, exacerbating the development of MASLD and ALD in mouse models.[367,368] In contrast, acetate, another type of SCFA, could block the IL-6/JAK1/STAT3 signaling pathway via binding to hepatocyte-derived GPR43, and reverse the development of MASLD-associated hepatocellular carcinoma.[369] This suggests the complex functions of SCFA components on liver injury. In addition, gut bacteria-derived lipopolysaccharides (LPS) influxes to the liver and activates Kupffer cells or macrophages expressing toll-like receptors (TLRs). These immune cells produce inflammatory cytokines and chemokines, thus augmenting innate immune responses and liver injuries.[370,371] On the other hand, the normal enterohepatic circulation of bile acids (BAs) is important for liver and intestinal homeostasis. BA is secreted by hepatocytes and modified in the intestine for lipid digestion and absorption. Upon liver injury, changes in BA composition and level, as well as decreased BA receptor (farnesoid X receptor; FXR) was reported.[372] Studies show that a decrease in intestinal FXR levels dampens the tight junctions of intestinal epithelial cells and promotes intestinal lipid absorption.[373,374] Additionally, knockdown of hepatocyte-derived FXR increases hepatic triglyceride content. In contrast,

activation or overexpression of FXR protects against liver injury by decreasing hepatic lipogenic gene expression, reducing intestinal lipid absorption, and promoting intestinal barrier integrity.[374,375]

The liver-brain axis is essential in the context of liver diseases. Upon acute or chronic injury, liver produces inflammatory cytokines and is unable to process ammonia from the intestine.[376,377] These cytokines, such as TNF-α and IL-1β, impair blood-brain barrier (BBB) function and structure, and lead to neuroinflammation.[378] In addition, lipid components such as ceramide and palmitate, along with peripheral insulin resistance in MASLD preclinical models, contribute to neuroinflammation and neurodegeneration.[379] Moreover, ammonia crosses the damaged BBB and is absorbed by astrocytes, leading to the conversion of ammonia to glutamine and subsequently causing cerebral edema and neuronal cell death.[380] Reciprocally, the central nervous system (CNS) exerts an influence on liver and intestine function. Changes in liver microenvironment are detected and transduced through hepatic vagal sensory afferent nerves to CNS, which feeds back the signal to liver vagal parasympathetic nerves.[381] For instance, CNS leptin signaling has been shown to promote hepatic triglyceride export and inhibit lipogenesis via the brain-vagus-liver axis, thereby attenuating the development of MASLD in animal models, as well as in a randomized, placebo-controlled crossover trial.[382,383] Chronic systemic inflammation involving liver-organ interactions, as well as bacteria translocation due to impaired intestinal barrier can further lead to acute-on-chronic liver injury and multiorgan failure.[384] In summary, dysregulation of the gut-liver-brain axis partially influence the progression of liver diseases, suggesting potential therapeutic strategies targeting this axis for liver disease management.

Adipose-liver axis

Emerging studies have demonstrated the pathological crosstalk between the liver and adipose tissue during liver diseases, especially MASLD and ALD. Fat overload leads to adipocyte hypertrophy, hyperplasia, and abnormal adipokine production.[385] Chronic ethanol exposure also disrupts the endocrine function of adipose tissue.[386] For example, adiponectin, which promotes liver glucose use and fatty acid oxidation, is found to be decreased in MASLD and ALD; however, exogenous supplementation of adiponectin alleviates high-fat diet- and ethanol-

induced liver steatosis and insulin resistance.[387] Leptin, an adipocyte-derived hormone that inhibits appetite and increases fatty acid oxidation, becomes resistant in MASLD. In obese individuals, higher serum leptin levels correlate with greater severity of liver inflammation.[388] In contrast, inflammatory cytokines such as IL-1β and TNF-α are produced by these diseased adipocytes. The inflammatory microenvironment recruits immune cell infiltration and further promotes adipocyte lipolysis. Systemic release of fatty acids and cytokines flows into the liver and aggravates liver steatosis and inflammation.[389]

Conversely, the liver also interacts with adipose tissue. Fibroblast growth factor-21 (FGF-21), an endocrine hormone mainly produced by hepatocytes, contributes to glucose uptake and adiponectin production in adipocytes.[390] In obesity, the expression of FGF-21 increases with the progression of MASH, whereas its effect on adipose tissue becomes resistant, as evidenced by decreased levels of adiponectin.[391] As mentioned above, a decrease in adiponectin exacerbates the development of MASLD and ALD by promoting liver steatosis and insulin resistance. In summary, pathological crosstalk between the liver and adipose tissue contributes to liver diseases.

Diagnosis, staging, prevention and therapeutic strategies

The diagnosis of liver disease usually includes the following steps: history collection, physical examination, laboratory tests, imaging examination, and histopathological examination. For several kinds of liver diseases, a liver biopsy may be required to confirm the diagnosis by pathological examination under a microscope. In the process of diagnosis, it is necessary to select targeted examination items according to the specific conditions of the patient to clarify or exclude the disease (Table 2). The accurate diagnosis of etiology and clinical staging is crucial for guiding treatment strategies and improving patient prognosis. Therefore, we have summarized the current diagnostic principles and staging plans for various liver diseases, while also providing an introduction to the corresponding treatment strategies as outlined in the guidelines.

The treatment approach for liver disease is comprehensive, encompassing etiological management, lifestyle modifications, pharmacotherapy, nutritional support, prevention and management of complications, regular monitoring, and health education. Irrespective of the

underlying cause, liver transplantation may represent the sole efficacious intervention for advanced liver disease following cirrhosis or hepatic failure.

Viral hepatitis

Due to the presence of multiple types of hepatitis viruses and the possibility of acute or chronic viral infections in patients, serological testing is necessary following a thorough history collection and physical examination.[392] Hepatitis virus antigen and corresponding antibody tests, along with etiological tests such as viral RNA load assessments, serve as crucial diagnostic indicators for identifying viral hepatitis in individuals presenting related symptoms.[393,394] The diagnosis of viral hepatitis can be established by considering the patient's clinical manifestations, evidence of liver function impairment in laboratory tests, and results from auxiliary imaging examinations while excluding other potential diseases that may present similar symptoms.

Vaccination is the most effective means of preventing infection with hepatitis A, B, and D viruses.[395] The recombinant HBV vaccine is both safe and highly efficacious, capable of being administered as a standalone immunization or in conjunction with other antigens utilized in infant immunization programs or alongside the hepatitis A virus vaccine. The treatment approach for viral hepatitis should be tailored to each individual patient's condition, including factors such as virus type, liver function status, presence of complications, and other relevant considerations. For patients afflicted by chronic or severe forms of hepatitis, antiviral therapy may be considered to impede progression towards cirrhosis, liver failure, and hepatocellular carcinoma. Antiviral agents like lamivudine, entecavir, and tenofovir can be employed for treating CHB while DAAs such as sofosbuvir and harvoni can be used against hepatitis C infections.[396,397] Currently, nucleos(t)ide analogues have demonstrated safety along with efficacy in inhibiting HBV replication; however, they rarely achieve clearance of HBsAg necessitating long-term administration to prevent recurrence. Therefore, various classes of DAAs and immunomodulatory therapies are currently under development aiming at achieving functional cure defined as persistent undetectable HBsAg levels along with absence of detectable HBV DNA after completion of limited duration treatment.[398,399] It might eventually require combination therapy involving multiple drug classes to attain this objective. Detailed

information of viral hepatitis managements can be found in updated AASLD and EASL clinical practice guidelines.[393,394,396,400-402]

Acute liver injury

Ancillary examinations revealed deranged liver function tests further supporting the initial diagnosis of acute liver injury (ALI).[403] Thorough medical history collection can aid in identifying potential risk factors for ALI, such as recent initiation of medications or herbal/nutritional supplements intake, exposure to possible pathogens, travel history, and vaccination status.[404] The definition of mild acute liver injury typically includes an ALT level between 2 and 5 times the upper limit of normal (ULN). Moderate ALI is usually defined as an ALT level between 5 times and 15 times the ULN. Severe ALI requires meeting specific criteria, including an international normalized ratio (INR) of ≥2.0, ALT levels of ≥10 ULN, and total bilirubin (TBiL) levels of ≥3.0 mg/dL without hepatic encephalopathy.[405] An increase in the INR indicates a poor prognosis for patients with severe ALI.[406] Studies have demonstrated that apart from etiology, bilirubin levels, INR values, and duration of jaundice are effective predictors for poor prognosis in ALI patients with specific thresholds such as duration of jaundice >3 days, TBil>51 μmol/L, and INR>1.7.[407]

The fundamental principles of ALI treatment encompass early identification and correction of reversible causes, judicious selection of medications, timely implementation of liver replacement therapy, and proactive prevention and management of complications.[408] Patients with abnormal liver function who do not yet meet the criteria for ALI should be closely monitored to promptly remove pathogenic factors in order to prevent liver damage or failure.[409] For patients with rapid disease progression or existing liver damage, drug therapy should be considered based on active monitoring and etiological treatment. Currently available hepatoprotective drugs can generally be categorized into agents that repair and protect the liver cell membrane, anti-inflammatory drugs, antioxidant drugs (e.g. NAC and glutathione), and cholestrogenic drugs (e.g. UDCA).[410,411] Presently, there is a lack of specific medications and approaches for treating advanced ALF. Thus, emphasis should be placed on symptomatic treatment while actively preventing complications. The use of adrenocortical hormones in the management of liver failure remains controversial; comprehensive consideration must be given

to etiology and patient monitoring indices before making a decision.[412,413] Cytokine therapies are under investigation. For example, An open-label, dose-escalation study utilizing IL-22 agonist F-652 to treat sAH has yielded promising results as anticipated, thereby offering a potential effective treatment strategy for further reducing the case fatality rate.[414,415] Guidelines related to artificial livers and liver transplantation can serve as references for their respective treatments.[416,417] Detailed information of acute liver failure and acute-on-chronic liver failure managements can be found in updated AASLD and EASL clinical practice guidelines (Fig. 6).[418-420]

MASLD

The diagnosis of MASLD is based on three criteria: (1) imaging diagnosis of hepatic steatosis and/or liver biopsy findings of ≥ 5% hepatocyte ballooning degeneration; (2) presence of one or more metabolic syndrome score components; (3) exclusion of other causes that may contribute to hepatic steatosis.[421] Ultrasound imaging is the preferred modality for diagnosing hepatic steatosis and monitoring hepatocellular carcinoma.[422] Liver stiffness measurement obtained through shear wave elastography can be utilized for non-invasive assessment of hepatic steatosis and fibrosis in patients with chronic liver disease. In addition to meeting the diagnostic criteria for MASLD, the presence of ≥ 5% hepatocyte ballooning degeneration combined with lobular inflammation and/or portal inflammation can lead to a diagnosis of MASH. Given the associated risks, a liver biopsy is typically reserved for cases where MASH is suspected but cannot be confirmed by other means. Currently, clinicians make comprehensive judgments based on individual patient circumstances to select appropriate diagnostic methods.[423]

The management of MASLD necessitates a multidisciplinary approach, encompassing strategies such as weight and waist circumference reduction, enhancement of insulin sensitivity, prevention of metabolic syndrome and T2DM, mitigation of MASH, and reversal of fibrosis. Dietary modification and increased physical activity through health education serve as the fundamental pillars in the treatment regimen for MASLD. Greater weight loss in overweight/obese individuals confers additional benefits on metabolic cardiovascular health and liver function. A gradual weight reduction ranging from 3% to 5% within one year can

reverse hepatic steatosis; a weight loss between 7% to 10% can alleviate MASH; more than 10% weight loss can lead to fibrosis regression; while a substantial decrease by 15% even improves T2DM symptoms.[424] Unhealthy habits such as irregular eating patterns, soft drink consumption, smoking tobacco products, or alcohol use should be avoided alongside sedentary behavior and physical inactivity.[425]

Combined presence of metabolic cardiovascular risk factors and liver injury necessitates appropriate pharmacological intervention. Patients with MASLD and a BMI ≥ 28 kg/m^2 may benefit from weight loss medications, while hypoglycemic drugs for weight reduction should be prioritized in the treatment of type 2 diabetes.[421,426] In managing diabetic patients with MASLD, preference should be given to drugs such as metformin, pioglitazone, SGLT-2 inhibitors, GLP-1 receptor agonists, and other agents that have potential hepatoprotective effects.[427] Statins are the primary choice for pharmacotherapy of arteriosclerotic lipid disorders in MASLD patients; however, caution or discontinuation is advised when using statins in individuals with severe liver diseases like decompensated cirrhosis.[428] ACE inhibitors or ARBs are recommended as first-line therapy for hypertension in MASLD patients, whereas non-selective β-blockers can be used concomitantly if clinically significant portal hypertension is present.[429] As an agonist of the thyroid hormone receptor-β (THR-β), resmetirom has recently gained FDA approval for the treatment of adult patients with MASH and liver fibrosis.[430] For non-cirrhotic MASLD patients who meet the criteria for metabolic surgery aimed at weight loss, options such as gastric bypass surgery, sleeve gastrectomy, duodenal transposition, or adjustable gastric banding may be considered to address MASH and fibrosis.[431] Liver transplantation could be an option for individuals with decompensated cirrhosis resulting from MASH complications or ACLF as well as those diagnosed with HCC.[432] Detailed information of MASLD managements can be found in updated AASLD and EASL clinical practice guidelines (Fig. 7).[421,433]

ALD

Alcohol-related liver disease is diagnosed in patients who typically have a prolonged history of alcohol consumption (more than 12 months, >2 drinks in women and >3 drinks in men per day) or recent heavy alcohol use within the past two weeks. The patient's clinical symptoms were

nonspecific and accompanied by abnormal serum liver function tests. Notably, an elevated AST/ALT ratio (>1.5), GGT levels, and mean corpuscular volume (MCV) values are characteristic of ALD.[434] These indicators can significantly decrease after cessation of alcohol intake and usually return to normal within four weeks (although GGT may take longer to normalize), which aids in diagnosis.[435] Imaging studies are used for diagnosis while excluding hepatitis viruses, medications, toxic liver injury, autoimmune liver disease, and other conditions.[436] Symptomatic alcoholic hepatitis is diagnosed in the presence of jaundice and the criteria proposed by the National Institute on Alcohol Abuse and Alcoholism: last alcoholic drink within 8 weeks of onset of jaundice; serum bilirubin level greater than 3 mg/dL; AST and alanine aminotransferase level less than 400 IU/L and AST level greater than 50 IU/mL, with the ratio of AST to alanine aminotransferase greater than 1.5:1; and other causes of liver disease, biliary obstruction, and hepatocellular carcinoma ruled out.[437]

The treatment principles of ALD include abstinence and nutritional support, reducing the severity of the disease, and providing symptomatic treatment for alcoholic cirrhosis and its complications.[438] Complete abstinence from alcohol enhances prognosis, mitigates liver histological injury, decreases portal vein pressure, delays fibrosis progression, and improves survival at all stages of the disease. Baclofen may be administered orally to individuals facing challenges with active abstinence.[434] Moreover, patients with ALD require adequate nutritional support, including a high-protein, low-fat diet based on alcohol abstinence. Additionally, attention should be given to vitamin supplementation.[439] Due to the limited expression of IL-22 receptor on epithelial cells, such as hepatocytes, IL-22 could serve as a specific target for preventing hepatocyte death and promoting hepatocyte proliferation without affecting immune cells. A multicenter trial is currently underway to investigate the use of IL-22Fc in treating ACLF, including severe alcoholic hepatitis (sAH) (CTR20212657).[415] Inflammation has been extensively studied as a therapeutic target for sAH treatment due to its significant role in the pathogenesis of alcoholic liver disease. Steroid therapy has been utilized since the 1970s and emerging data suggest that it improves short-term survival in some sAH patients without impacting long-term survival.[435] Treatment for anti-hepatic fibrosis should be prioritized along with actively addressing complications related to alcoholic cirrhosis such as esophageal and gastric varices rupture bleeding, spontaneous bacterial peritonitis, hepatic encephalopathy, and

hepatocellular carcinoma.[440] Liver transplantation may be considered for patients with severe alcoholic cirrhosis; however, many transplant centers require patients to abstain from alcohol for six months before undergoing surgery.[441] Detailed information of ALD managements can be found in updated AASLD and EASL clinical practice guidelines (Fig. 7).[439,442]

AIH

Patients with AIH often exhibit mild to moderate elevation of ALT and AST. On the other hand, patients with PBC and PSC frequently present elevated serum ALP and GGT levels, along with increased bilirubin levels in advanced stages.[134] Serum autoantibodies such as anti-nuclear antibody (ANA) and anti-smooth muscle antibody (ASMA) can be detected in AIH patients, accompanied by elevated IgG levels.[443] In 90-95% of PBC patients, serum antibodies against mitochondria (AMA) and elevated IgM levels can be found.[444] Ultrasound, CT scan, MRI, endoscopic retrograde cholangiopancreatography (ERCP), and magnetic resonance cholangiopancreatography (MRCP) can be utilized to exclude biliary diseases like tumors or stones affecting the hepatobiliary system.[136] ERCP is considered the "gold standard" for diagnosing PSC; however, MRCP is preferred due to its non-invasive nature when diagnosing this condition initially.[445,446] Liver histological biopsy serves as a means to differentiate between causes of liver injury and evaluate tissue damage.[447] Liver histological biopsy serves as a means to differentiate between causes of liver injury and evaluate tissue damage.[447] The Paris diagnostic criteria and the AIH simplified diagnostic system are widely utilized for diagnosing AIH and its overlap with PBC. Among these, the Paris criteria stand out as the most common and effective tool for diagnosing AIH-PBC overlap syndrome.[448] In 2008, the International Autoimmune Hepatitis Group introduced a simplified diagnostic scoring system for AIH, which proves valuable in identifying patients with AIH-PBC requiring corticosteroid treatment.[449]

The treatment and prognosis of different autoimmune liver diseases vary significantly. Immunosuppressive therapy is the primary approach for managing AIH, with a combination of prednisolone and azathioprine being the preferred treatment for AIH patients.[450] In cases of poor response, alternative immunosuppressive agents can be considered. UDCA is the first-line option for PBC treatment, while additional medications such as bate drugs, budesonide, and obeticholic acid may be added if necessary.[451] Currently, there are no drugs available to alleviate

liver damage caused by PSC. Therefore, the focus lies in controlling complications and monitoring liver damage. UDCA can improve liver biochemical markers, reduce liver fibrosis severity, and enhance imaging findings related to biliary tract involvement.[452] Glucocorticoids are favored for inducing remission in IGG4-associated hepatobiliary diseases.[453] Patients who seek early treatment for AIH generally exhibit better treatment responses and prognoses comparable to those of healthy individuals in the long term. Conversely, patients who delay seeking treatment or do not respond well to therapy have an increased risk of developing cirrhosis and liver failure. Liver transplantation remains the sole effective intervention for end-stage liver disease.[454] Detailed information of AIH managements can be found in updated AASLD and EASL clinical practice guidelines.[454,455]

Genetic and rare liver diseases

Inherited liver diseases exhibit overlapping clinical manifestations, often requiring multiple clinical or pathological features for diagnosis.[100] Genetic testing is the most crucial tool in diagnosing hereditary liver diseases. However, due to the complexity and diversity of genetic mutations in genetic liver disease, gene analysis and diagnosis remain challenging due to factors such as high cost, poor detection sensitivity, and the close relationship between heredity and acquired environment.[456] Some rare or inherited liver diseases have unique clinical manifestations that aid in their diagnosis; for example, the combination of Kayser-Fleischer rings and a low serum ceruloplasmin (<0.1 g/L) level are prominent features of Wilson's disease while decreased serum α1-antitrypsin levels with pulmonary damage such as emphysema suggest a1 antitrypsin deficiency.[193] Notably, these features are not always reliable, and additional tests, such as high-quality imaging tests (e.g., MRI) or liver tissue biopsies, can also assist in making a definitive diagnosis.[457]

Several treatment options are available to alleviate symptoms and maintain optimal liver function for inherited liver disease. Dietary modifications may be necessary, such as adhering to a low-copper diet in cases of Wilson's disease, and avoiding foods rich in copper like animal organs, dried fruits, and mushrooms.[458] Symptomatic treatment often involves the use of medications that facilitate copper excretion or inhibit its absorption.[459] Additionally, patients with liver damage can benefit from appropriate hepatoprotective therapy. For those

experiencing neuropsychiatric symptoms, consultation with a neurologist is recommended for tailored management strategies. Itch relief can also be achieved through pharmacological interventions. In rare instances where hereditary liver diseases lead to ALF or decompensated cirrhosis unresponsive to conventional treatments or intolerant reactions occur, consideration should be given to liver transplantation.[460] Detailed information of Wilson disease managements can be found in updated AASLD and EASL clinical practice guidelines.[100,461]

Liver cirrhosis

The diagnosis of liver cirrhosis should be comprehensive, taking into account clinical manifestations of hepatic hypofunction and portal hypertension, as well as imaging and endoscopy findings, and laboratory results. Liver biopsy is recommended for patients with diagnostic difficulties, while etiological screening should be conducted whenever possible.[462] Typical features observed in abdominal ultrasound, CT scans, and MRI images of cirrhosis include changes in liver volume (early enlargement followed by late contraction), abnormal ratio between the left and right lobes (shrinkage of the right lobe with enlargement of the left lobe and caudate lobe), irregular or jagged liver contour, widening of liver clefts, uneven liver echo or density signal distribution, dilation of the portal vein, and collateral circulation expansion.[155] Transient elastography-derived liver stiffness measurement (LSM) demonstrates a strong ability to evaluate significant liver fibrosis and cirrhosis but exhibits poor accuracy in assessing mild stages of fibrosis. Magnetic resonance elastography (MRE) offers high diagnostic accuracy along with good stability and efficiency for staging liver fibrosis because it is less influenced by factors such as obesity or ascites; however, it requires relatively more time for examination and is expensive.[463] Serological indicators such as aspartate aminotransferase-platelet ratio index (APRI) and fibrosis-4 index (FIB-4) exhibit low sensitivity and specificity in diagnosing cirrhosis. Moreover, the critical value used to determine liver fibrosis/cirrhosis can also be affected by etiology among other factors.[464] According to the presence of esophageal and gastric varices, hemorrhage, ascites, hepatic encephalopathy, and jaundice, cirrhosis is classified into six stages. Stage 1 does not exhibit varicose veins or any other complications; it is further divided into stages 1a and 1b based on whether the hepatic venous pressure gradient (HVPG) is ≥10 mmHg. Varicose veins appear in

stage 2 but without EGVB (esophagogastric variceal bleeding) or ascites. EGVB occurs in stage 3 but without decompensation such as ascites or hepatic encephalopathy. Stage 4 includes various forms of decompensation except for EGVB, including ascites, overt hepatic encephalopathy, overt bacterial infection, and non-obstructive jaundice. Stage 5 presents two types of decompensations while stage 6 is characterized by recurrent infection, dysfunction of extrahepatic organs, ACLF, refractory ascites, persistent hepatic encephalopathy or jaundice.[462,465]

The most crucial treatment for cirrhosis is the removal of its underlying cause. Etiological control, particularly antiviral therapy in patients with hepatitis B/C, as well as abstinence in those with alcoholic cirrhosis, can potentially reverse liver fibrosis and cirrhosis or restore compensatory stage in decompensated cirrhosis patients.[466] In cases where malnutrition complicates cirrhosis, it is recommended to consume 25-35 kcal/kg/d energy intake, 1.0-1.5 g/kg/d protein intake, increase meal frequency, add extra meals at night, and adequately supplement dietary fiber, vitamins, and trace elements. Patients with ascitic cirrhosis should moderately restrict sodium intake (85-120 mmol/d or equivalent to 5.0-6.9 g/d salt) while avoiding extreme sodium restriction (<40 mmol/d). Unless moderate to severe dilutive hyponatremia (blood sodium <125 mmol/L) is present, water intake does not generally need to be restricted in patients with ascites due to cirrhosis.[467,468] Diuretics are considered the first-line treatment for ascites in cirrhotic patients; spironolactone alone or combined with furosemide or torasemide can be used. Grade 2 or 3 ascites that are unresponsive to conventional diuretics may be managed with the administration of tolvaptan.[469] Massive paracentesis is a commonly employed intervention for refractory ascites, and albumin infusion should be utilized to optimize intravascular volume expansion. Teripressin represents an efficacious pharmacological option for the treatment of refractory ascites.[470] Transjugular intrahepatic portosystemic shunt (TIPS) should be considered in cases where therapy for massive ascites proves ineffective.[471] Liver transplantation serves as the definitive therapeutic approach for decompensated cirrhosis and warrants evaluation when patients develop esophageal variceal bleeding, refractory ascites, hepatorenal syndrome, hepato-pulmonary syndrome, recurrent hepatic encephalopathy, ACLF, or HCC.[472] Detailed information of cirrhosis managements can be found in updated AASLD and EASL clinical practice guidelines.[462,473-476]

HCC

Traditionally, the diagnosis of HCC has been primarily based on cytology or histology. However, with advancements in staged perfusion angiography during CT and MRI cross-sectional imaging, HCC can now be reliably diagnosed radiologically in cirrhotic patients under surveillance without the need for biopsy.[477] Abdominal ultrasound is currently considered the most recommended method for monitoring HCC.[477,478] Although serum alpha-fetoprotein (AFP) alone lacks sensitivity and specificity to serve as an independent monitoring test, its combination with ultrasound significantly improves early detection sensitivity for HCC.[479] Various integrated imaging and blood-based strategies have been proposed to enhance early detection of HCC; nevertheless, most of these approaches have only been evaluated through case-control studies and require prospective validation.[480]

Over the past two decades, the Barcelona Clinic Liver Cancer (BCLC) staging system has gained recognition from the majority of professional societies.[481] However, managing HCC involves a complex decision-making process, and the availability of treatment options varies significantly among medical centers across different countries. Consequently, effective HCC management necessitates multidisciplinary collaboration to devise tailored strategies that cater to each patient's unique circumstances in order to achieve optimal outcomes.[482] Surgical treatment options for hepatocellular carcinoma include surgical resection and liver transplantation, both considered potentially curative treatments. However, it is important to note that nearly 70% of patients experience recurrent HCC after resection.[483] Liver transplantation stands out as the most definitive treatment option for early-stage hepatocellular carcinoma since it allows removal not only of the tumor but also an unhealthy liver with limited functional capacity. A retrospective multicenter study involving 187 HCC patients revealed that 58% underwent successful downstaging followed by liver transplantation with a 5-year survival rate reaching 80%.[484] Percutaneous local ablation is a potentially curative treatment modality that can be employed in patients with early HCC. The two most commonly utilized techniques are radiofrequency ablation (RFA) and microwave ablation (MWA).[485] MWA demonstrates enhanced efficacy for larger tumors measuring 3-4 cm, and requires less procedural time compared to RFA.[486] Transarterial chemoembolization (TACE) is a highly effective treatment

modality for patients with intermediate-stage HCC. Transarterial radiation embolization (TARE) represents an alternative local regional therapy approach, which can be employed as the primary therapeutic intervention for unresectable HCC cases.[487] Unlike TACE, TARE involves intratumoral brachytherapy techniques and exerts minimal embolic effects on hepatic artery distribution, making it suitable even for patients presenting portal vein thrombosis or tumor invasion (Fig. 8).[488]

The treatment strategy for HCC has been significantly revolutionized by the introduction of systemic pharmacological therapy worldwide.[489] Sorafenib, lenvatinib, cabotinib, ramociumab, and other drugs have successively gained approval for HCC treatment.[490-492] However, due to the heterogeneity and complexity of HCC pathogenesis, precise treatment for this disease is still under investigation. Concurrently, immune checkpoint inhibitors have emerged as a promising therapeutic option for advanced hepatocellular carcinoma.[493] Various immunotherapies including checkpoint inhibitor combined targeted therapy, checkpoint inhibitor combination therapy, and non-checkpoint inhibitor immunotherapy (such as immune cell adoptive therapy) have shown significant efficacy.[494,495] In conclusion, active research is still required in this field and a combination of treatment modalities may enhance therapeutic options for patients. Detailed information of HCC managements can be found in updated AASLD and EASL clinical practice guidelines.[482,496]

Clinical research progress

Acute liver disease-viral hepatitis

Acute viral hepatitis, resulting from viruses such as HAV-HEV, primarily benefits from prevention via inactivated vaccines.[497] Currently, there is no specific antiviral for HAV, but clinical studies suggest benefits from steroids and interferon-β in improving outcomes.[498] For severe acute HBV, early administration of lamivudine and entecavir has been shown to improve patient conditions and reduce progression to chronic hepatitis.[499,500] Therapeutic strategies such as ledipasvir/sofosbuvir have been effective in treating HIV and HBV co-infections by shortening treatment durations.[501] Meanwhile, grazoprevir combined with elbasvir shows promise in acute HCV, particularly for genotypes 1 or 4.[502] For HEV, ribavirin has been effective in reducing viral load (Table 3).[503] Research on treatment for acute HDV is limited and warrants

further exploration. Additional studies reveal that other virus like adenovirus, cytomegalovirus (CMV), Epstein-Barr virus (EBV), and TT virus can also cause acute viral hepatitis.[504,505] Rare viral hepatitis are often overlooked due to their low incidence. These less common forms underline the need for heightened clinical awareness to prevent misdiagnosis and inappropriate treatment.

Acute liver disease-DILI

Acute DILI, often caused by substances such as acetaminophen, antibiotics, or anti-inflammatory drugs, usually resolves with decreasing liver enzyme levels within days to weeks, with fewer than 10% of cases progressing to chronic liver damage.[506] Acetaminophen overdose is primarily treated with N-acetylcysteine (NAC).[507] Investigating additional treatments that can protect liver function during both the early and late stages of DILI is critical for minimizing long-term damage.

Acute liver disease-ALD

Acute ALD remains challenging, and therapies such as pentoxifylline and corticosteroids shown to decrease short-term, but not medium-term, mortality.[508] Although corticosteroids may reduce short-term mortality, their long-term safety profile is concerning due to the risk of severe infections.[509] TNF-α inhibitors, such as infliximab and etanercept, reduce inflammation but increase infection risks.[510] There is emerging interest in the role of intestinal microbes in ALD, though clinical validations are still preliminary.[511]

Chronic liver disease-viral hepatitis

Innovations in CHB treatments include Vebicorvir (VBR), a core inhibitor that has shown superior efficacy compared to traditional nucleoside reverse transcriptase inhibitors.[512] Tenofovir alafenamide is used for multidrug-resistant HBV strains, improving long-term outcomes.[513] PD-1 inhibitors and RNA interference therapies like ARC-520 are under investigation for their potential to enhance immune responses and reduce viral load in HBV patients.[514,515] Treatments for chronic HCV have evolved with the development of DAAs, reducing concerns about VZV reactivation.[516] Glecaprevir and pibrentasvir have shown

improved responses in HCV patients who failed prior DAA therapies.[517] Bulevirtide combined with tenofovir disoproxil fumarate offers new hope for HDV patients, although more studies are needed to confirm these findings.[518,519] Overall, these advancements represent significant progress in the treatment of liver diseases, but ongoing research is crucial to optimize safety and long-term efficacy of these therapies.

Chronic liver disease-ALD

No FDA-approved medications currently exist specifically for ALD. Research indicates that alcohol consumption disrupts the intestinal microbiota, sometimes leading to an overgrowth of *Candida albicans*. This alteration suggests that probiotics could help mitigate ALD by modulating intestinal flora.[520,521] Inflammation is a critical factor in ALD pathogenesis, hence steroids are used to manage symptoms and improve short-term survival rates, although their long-term efficacy is still not well-established.[522] Corticosteroids treatments seem to be effective, which needs adequate nutritional intake throughout the treating duration.[523] Efforts to target inflammation with cytokines such as IL-1 and TNF-α have been explored, but results, including attempts to combine IL-1 receptor antagonists with pentoxifylline and zinc, have not shown superior outcomes compared to corticosteroids alone.[524,525] Future research is essential to develop more targeted therapies for ALD.

Chronic liver disease-MASLD

With rising global obesity rates, MASLD prevalence is expected to increase.[526] In a significant development, in 2020, saroglitazar was approved by India's Drug Administration as the first medication specifically for MASLD, demonstrating effectiveness in reducing ALT levels, liver fat content, insulin resistance, and atherogenic dyslipidemia.[527] Furthermore, in March 2024, Resmetirom became the first FDA-approved drug for treating non-cirrhotic NASH with moderate to advanced liver fibrosis in adults, marking a major milestone in MASLD treatment.[528] Despite these advancements, there remains a substantial need for more precise non-invasive diagnostic techniques and effective treatments.

Chronic liver disease-autoimmune liver diseases

Recent advances in AIH research have improved our understanding of its causes, diagnosis, and treatment. Glucocorticoids and immunosuppressants remain treatment mainstays,[529] with studies showing that combining mycophenolate mofetil (MMF) with prednisolone can lead to better outcomes and fewer side effects.[530] Future directions include advancing personalized treatment strategies to enhance patient quality of life and prognosis. Cholestatic liver diseases such as PBC and PSC are primarily managed with ursodeoxycholic acid (UDCA).[531] Emerging treatments, such as obeticholic acid, are undergoing evaluation for their efficacy in these diseases.[532] For PSC, liver transplantation remains a definitive but severe option, underscoring the ongoing need for research into pharmacological interventions that could slow disease progression.

End-stage liver disease-cirrhosis

As a critical aspect of end-stage liver disease, cirrhosis has undergone significant advancements in both diagnosis and management. The integration of non-invasive tests like transient elastography and serum biomarkers (e.g., FibroTest) into clinical practice has greatly improved the early detection of liver fibrosis and cirrhosis, reducing reliance on invasive biopsy procedures.[533] Recent studies have highlighted the potential of pegbelfermin and aldafermin in ameliorating liver fibrosis associated with MASH and compensated cirrhosis.[534,535] Additionally, anti-fibrotic medications and treatments aimed at enhancing liver microcirculation are showing promising results in clinical trials. Research into the therapeutic application of mesenchymal stem cells for decompensated cirrhosis has also yielded positive outcomes, although more extensive studies are required to confirm these findings.[536,537] Liver transplantation continues to be the sole curative treatment for cirrhosis, underscoring the ongoing need for the development of more effective therapies.

End-stage liver disease-liver failure

Liver failure represents the most severe manifestation of end-stage liver disease, where managing both acute and chronic forms remains a formidable challenge. Recent advancements include the development of the MKK4 inhibitor HRX215, which has been shown to promote liver regeneration and prevent liver failure.[538] Extracorporeal liver support devices have also

shown promise in improving patient outcomes in acute and chronic liver failure, potentially reducing mortality rates.[539] Innovations in regenerative medicine, including stem cell therapies and liver bioengineering, are being explored as novel treatment avenues.[540] Moreover, improvements in liver transplantation techniques and refinement of immunosuppressive treatments are crucial for enhancing patient survival and quality of life.

End-stage liver disease-HCC

There has been notable progress in clinical research on HCC, yielding several pioneering treatments. Research comparing PD-1 antibody carrelizumab with VEGFR2-targeted TKI rivoceranib has indicated better progression-free and overall survival rates in patients with unresectable HCC compared to standard treatments like sorafenib.[541] In high-risk post-resection HCC, the PD-1 inhibitor sintilimab shows potential in reducing tumor recurrence.[542] The LAUNCH trial revealed that combining lenvatinib with transarterial chemoembolization (TACE) significantly enhances clinical outcomes in patients with advanced HCC.[543] Furthermore, a phase 1/2 trial exploring a personalized neoantigen vaccine (PTCV) combined with pembrolizumab has demonstrated promising immune responses and preliminary efficacy in advanced HCC cases.[544] For patients at high risk of HCC recurrence after postoperative ablation, atezolizumab combined with bevacizumab improves recurrence-free survival.[545] These emerging therapies offer new hopes and strategies in the fight against HCC, potentially transforming the therapeutic landscape.

Conclusions and perspectives

The etiology of liver diseases is continually evolving. With the global rise in obesity and T2DM, MASLD poses a growing health threat worldwide. The administration of vaccination and antiviral medications has significantly decreased the incidence of viral hepatitis in the Americas and Europe; however, the prevalence of MASLD, ALD, and DILI is rising. Despite advancements in vaccinations and antiviral medications that effectively prevent and combat viral infections, chronic hepatitis B and C remain prevalent, particularly in low-income countries lacking adequate medical resources. Additionally, the incidence of ALD is increasing, especially among younger populations. Despite heightened public health efforts, liver diseases

significantly contribute to the global disease burden.[546]

The diagnosis of liver diseases primarily depends on liver biopsy, an invasive technique unsuitable for broad screening. The absence of reliable biomarkers for the precise diagnosis and staging of specific liver diseases poses a significant challenge.[547] As such, developing novel non-invasive biomarkers and methods is crucial for the early detection of asymptomatic liver diseases. Such advancements could help identify high-risk individuals sooner, allowing for early interventions to halt disease progression.

Although there has been notable progress in understanding liver disease pathogenesis through advanced technologies, therapeutic options approved by the FDA remain limited, and existing medical interventions often provide minimal long-term survival benefits. The complexity of liver disease pathophysiology and the substantial heterogeneity in disease phenotypes mean that current mouse models do not adequately mimic the full spectrum of human liver diseases, including ALD and MASLD. Significant disparities exist between mouse models and human conditions in terms of pathophysiology and treatment outcomes, as numerous clinical trials have shown that drugs effective in mouse models fail to offer clinical benefits in humans.[548] The properties of chemical absorption, distribution, metabolism, excretion, and toxicity differ between species, resulting in drug doses that are beneficial and non-toxic in mice showing insufficient efficacy or causing side effects in humans. For example, galectin-3, which was demonstrated to reduce liver inflammation and fibrosis in murine MASH models, did not translate effectively to human patients.[549] Belapectin, a galectin-3 inhibitor, failed to show efficacy in MASH patients with cirrhosis and portal hypertension in a phase 2b randomized trial, possibly due to inadequate treatment duration and dosage. Pharmacokinetic analysis revealed differences in the metabolism of Belapectin between mice and humans.[550] This discrepancy underscores the need for the development of more standardized mammalian models, such as pigs and chimpanzees, which might bridge these gaps and improve translational success.

Moving forward, our current understanding of the pathogenesis has provided valuable insights and directed ongoing research efforts aimed at liver disease treatment. However, a comprehensive understanding of critical signaling pathways and their interactions during liver disease progression is essential to advance therapeutic strategies and improve patient outcomes.

20240930140919103813257768300953

ORIGINALITY REPORT

7%
SIMILARITY INDEX

PRIMARY SOURCES

1	jamanetwork.com<br>Internet	94 words — 1%
2	www.science.gov<br>Internet	49 words — 1%
3	Carlos López López, Mariona Calvo, Juan Carlos Cámara, Beatriz García-Paredes et al. "SEOM-GEMCAD-TTD clinical guidelines for the management of hepatocarcinoma patients (2023)", Clinical and Translational Oncology, 2024<br>Crossref	32 words — < 1%
4	www.mdpi.com<br>Internet	30 words — < 1%
5	Naim Alkhouri, Stephen A. Harrison. "Need-to-Know NAFLD - The Complete Guide to Nonalcoholic Fatty Liver Disease", CRC Press, 2023<br>Publications	29 words — < 1%
6	saudijgastro.com<br>Internet	24 words — < 1%
7	www.easl.eu<br>Internet	24 words — < 1%

8 Mengyao Yan, Shuli Man, Benyue Sun, Long Ma, Lanping Guo, Luqi Huang, Wenyuan Gao. "Gut liver brain axis in diseases: the implications for therapeutic interventions", Signal Transduction and Targeted Therapy, 2023 — 23 words — < 1%
Crossref

9 Amani Elshaer, David M. H. Chascsa, Blanca C. Lizaola-Mayo. "Exploring Varied Treatment Strategies for Metabolic Dysfunction-Associated Steatotic Liver Disease (MASLD)", Life, 2024 — 22 words — < 1%
Crossref

10 George A. Bray. "Handbook of Obesity -- Volume 1 - Epidemiology, Etiology, and Physiopathology, Third Edition", CRC Press, 2019 — 21 words — < 1%
Publications

11 worldwidescience.org — 18 words — < 1%
Internet

12 www.f6publishing.com — 16 words — < 1%
Internet

13 Lu Wang, Xiaoqing Zeng, Zida Wang, Ling Fang, Jia Liu. "Recent advances in understanding T cell activation and exhaustion during HBV infection", Virologica Sinica, 2023 — 14 words — < 1%
Crossref

14 Zhou, Zhou, Ming-Jiang Xu, and Bin Gao. "Hepatocytes: a key cell type for innate immunity", Cellular and Molecular Immunology, 2015. — 14 words — < 1%
Crossref

15 "Clinical Dilemmas in Viral Liver Disease", Wiley, 2010 — 13 words — < 1%
Crossref

16 www.jctres.com
Internet
13 words — < 1%

17 www.dvm360.com
Internet
12 words — < 1%

18 www.fastercapital.com
Internet
12 words — < 1%

19 www.stabroeknews.com
Internet
12 words — < 1%

20 Mi Na Kim, Ji Won Han, Jihyun An, Beom Kyung Kim et al. "KASL Clinical Practice Guidelines for Noninvasive Tests to Assess Liver Fibrosis in Chronic Liver Disease", Clinical and Molecular Hepatology, 2024
Crossref
11 words — < 1%

21 insights.omnia-health.com
Internet
11 words — < 1%

22 Ann Richmond, Raymond Kaempfer. "Cytokines revisited at Hilton Head", Cytokine & Growth Factor Reviews, 2000
Crossref
10 words — < 1%

23 Carolyn D. Berdanier. "Handbook of Nutrition and Food", CRC Press, 2019
Publications
10 words — < 1%

24 Roberto Candia, Blanca Norero, Carlos Agüero, Luis Díaz et al. "Validation of the Simplified Criteria for the Diagnosis of Autoimmune Hepatitis in Chilean-Hispanic Patients", Annals of Hepatology, 2017
Crossref
10 words — < 1%

25 xiahepublishing.com
Internet

10 words — < 1%

26	AB Pant. "Chapter 98 Allergic Rhinitis", Springer Science and Business Media LLC, 2024<br>Crossref	9 words — < 1%
27	F Durand. "Wilson's disease with severe hepatic insufficiency: beneficial effects of early administration of D-penicillamine", Gut, 2001<br>Crossref	9 words — < 1%
28	cris.maastrichtuniversity.nl<br>Internet	9 words — < 1%
29	sciendo.com<br>Internet	9 words — < 1%
30	www.frontiersin.org<br>Internet	9 words — < 1%
31	Mary T. Keogan, Eleanor M. Wallace, Paula O’Leary. "Concise Clinical Immunology for Healthcare Professionals", Routledge, 2006<br>Publications	8 words — < 1%
32	Min De ZENG. "Guidelines for the diagnosis and treatment of alcoholic liver disease", Journal of Digestive Diseases, 5/2008<br>Crossref	8 words — < 1%
33	Philip, George. "Liver Transplant Wait-List Trends in Young American Adults", Queen's University (Canada), 2021<br>ProQuest	8 words — < 1%
34	bmcpediatr.biomedcentral.com<br>Internet	8 words — < 1%

35	Ashwani K. Singal, Philippe Mathurin. "Diagnosis and Treatment of Alcohol-Associated Liver Disease", JAMA, 2021<br>Crossref	7 words — < 1%
36	Niederau, Claus. "Chronic hepatitis B in 2014: Great therapeutic progress, large diagnostic deficit", World Journal of Gastroenterology, 2014.<br>Crossref	7 words — < 1%

EXCLUDE QUOTES	OFF	EXCLUDE SOURCES	OFF
EXCLUDE BIBLIOGRAPHY	ON	EXCLUDE MATCHES	OFF
