# Supplementary material for: Liver diseases: epidemiology, causes, trends and predictions
Source: Signal Transduct Target Ther. 2025 Feb 5;10:33. doi: 10.1038/s41392-024-02072-z (PMC11794951; doi:10.1038/s41392-024-02072-z)
Supplement: Supplementary file 2 — Smilarity check-part 2 [file 41392_2024_2072_MOESM2_ESM.pdf]

20240930140611103813178797525811

WORD COUNT 9866

TIME SUBMITTED 30-SEP-2024 02:06PM

PAPER ID 112029075

Liver diseases: epidemiology, causes, trends and predictions

ABSTRACT

As a highly complex organ with digestive, endocrine, and immune-regulatory functions, the liver is pivotal in maintaining physiological homeostasis through its roles in metabolism, detoxification, and immune response. Various factors including viruses, alcohol, metabolites, toxins, and other pathogenic agents can compromise liver function, leading to acute or chronic injury that may progress to end-stage liver diseases. While sharing common features, liver diseases exhibit distinct pathophysiological, clinical, and therapeutic profiles. Currently, liver diseases contribute to approximately 2 million deaths globally each year, imposing significant economic and social burdens worldwide. However, there is no cure for many kinds of liver diseases, partly due to a lack of thorough understanding of the development of these liver diseases. Therefore, this review provides a comprehensive examination of the epidemiology and characteristics of liver diseases, covering a spectrum from acute and chronic conditions to end-stage manifestations. We also highlight the multifaceted mechanisms underlying the initiation and progression of liver diseases, spanning molecular and cellular levels to organ networks. Additionally, this review offers updates on innovative diagnostic techniques, current treatments, and potential therapeutic targets presently under clinical evaluation. Recent advances in understanding the pathogenesis of liver diseases hold critical implications and translational value for the development of novel therapeutic strategies.

Introduction

The liver, a multifaceted organ, is central to regulating physiological processes including metabolism, detoxification, protein synthesis, and immune response.[1] These functions are primarily mediated by hepatocytes, the major parenchymal cells within the liver. Supporting these are liver non-parenchymal cells (NPCs) — liver sinusoidal endothelial cells (LSECs), hepatic stellate cells (HSCs), cholangiocytes, Kupffer cells (KCs), and other immune cell types that maintain liver homeostasis.[2] Liver sinusoids are lined by LSECs with characteristic fenestrations, which facilitate substantial exchanges between sinusoids and hepatocytes. HSCs

reside in the space of Disse, secreting cytokines and growth factors that nurture neighboring cells. Cholangiocytes line the intra- and extrahepatic ducts of the biliary tree and contribute to the modification of hepatocyte-derived bile. KCs, along with other immune cells, play a pivotal role in defending against pathogens from the portal circulation.[2]

Liver diseases represent a wide array of disorders characterized by hepatocyte injury, inflammatory cell infiltration, and HSC activation, which cumulatively impair liver function and disrupt its architecture.[3] Annually, liver diseases are linked to approximately 2 million deaths and account for 4% of global mortality.[4] Acute liver diseases often result from hepatotropic virus infections, though drug-induced liver injury (DILI) is also becoming increasingly prevalent worldwide. Chronic liver conditions, on the other hand, typically arise from factors like alcohol consumption, hepatitis B virus (HBV), and hepatitis C virus (HCV) infections, along with a rising incidence of metabolic dysfunction-associated steatotic liver disease (MASLD) globally.[5] Progression from such chronic conditions to end-stage liver diseases, including cirrhosis and liver cancer, contributes significantly to morbidity and mortality.[4]

Despite often presenting similar clinicopathological features—ranging from asymptomatic stages to nonspecific digestive symptoms—these liver diseases share biochemical and histological profiles that complicate their differentiation based on a single diagnostic parameter.[6] Accurate diagnosis typically requires a combination of clinical presentation, specific biomarkers, and liver biopsy. Currently, clinical management of liver diseases largely focuses on hepatocyte protection, cause elimination, and symptom alleviation.[7] The removal of causative agents such as ethanol and viruses does not always prevent progression to cirrhosis, suggesting that the underlying mechanisms driving disease onset and progression are incompletely understood.[8] Thus, this review aims to provide an updated, comprehensive overview of the epidemiology and characteristics of liver diseases, highlight the complex pathogenetic mechanisms involved, and summarize the current clinical treatments and investigational drugs in clinical trials that hold potential for future therapeutic management.

Epidemiology of liver diseases

Global mortality

Liver disease stands as a leading cause of global mortality. The Global Burden of Disease 2019 study reported that 1.26 million individuals succumbed to cirrhosis and other chronic liver diseases in 2019, marking a 13% increase since 1990 (Fig. 1 and Table 1).[9] Liver cancer, a terminal outcome of liver disease, accounted for approximately 830,000 deaths in 2020, representing 8.3% of global cancer-related deaths.[10] Viral hepatitis, especially HBV and HCV, annually leads to around 1.3 million deaths.[11] Moreover, approximately 3.3 million people are diagnosed with alcohol-associated liver disease (ALD) annually, accounting for 5.9% of global deaths.[12] The rising fatalities from MASLD are also noteworthy, with an estimated 280,000 deaths in 2019.[13] Notably, liver disease mortality rates show significant regional disparities; for example, Mongolia reports the highest liver cancer mortality rate at 71.0 per 100,000 individuals, compared to 6.6 in the United States (U.S.).[10] This stark contrast arises primarily from the higher prevalence of HBV and HCV, limited healthcare resources, and elevated levels of alcohol consumption in Mongolia. Conversely, the U.S. benefits from effective hepatitis vaccination programs, comprehensive screening, and advanced treatment options, resulting in significantly lower mortality rates. While global trends indicate an increase in liver disease mortality, some high-income countries such as the U.S. have observed a decline since peaking in 2013, with an annual decrease of 3.2%.[14] These variances underscore differences in disease burden, healthcare access, and public health strategies across different regions and countries.

Global morbidity

Liver disease incidence is on the rise worldwide, posing an increasing risk of morbidity. Despite intensified global public health interventions, liver diseases continue to represent a significant portion of the global disease burden, underscoring the complexity and multidimensionality of liver disease epidemiology. The shift towards lifestyle-associated liver diseases, such as MASLD and ALD, is particularly alarming. This trend is closely linked to changes in global dietary habits, sedentary behavior, and rising obesity rates.[15,16] Recent meta-analyses have identified MASLD as the most common chronic liver disease, affecting 38.0% of the global adult population between 2016-2019.[17] Additionally, the incidence of ALD is climbing, paralleling increases in global alcohol consumption.[18] This trend is evident in regional data that show significant correlations between increasing alcohol consumption and ALD rates. In the

U.S., the age-adjusted death rate from ALD increased by 34.4% from 2009 to 2016, rising from 9.6 to 12.9 per 100,000 population, corresponding with a 7% increase in per capita alcohol consumption.[19] Similarly, the United Kingdom (U.K.) experienced a 43% increase in hospital admissions for ALD between 2002 and 2019, accompanied by a 10% increase in alcohol sales.[20] In South Korea, the age-standardized prevalence of ALD nearly doubled from 3.8% in 1998 to 7.4% in 2016, corresponding with a 43% increase in per capita alcohol consumption.[21] Moreover, China has also seen a significant surge, the prevalence of ALD among patients with chronic liver diseases increased from 4.3% in 2000 to 8.7% in 2015, concurrent with a 70% increase in recorded alcohol consumption between 2005 and 2016.[22] While new infections of HBV and HCV are declining in many regions due to effective public health interventions, chronic infections continue to pose a global challenge. The reduction in new infections can be attributed primarily to several key health measures beyond vaccination. Enhanced screening protocols for blood and organ donors have significantly reduced the risk of transfusion-associated hepatitis.[23] Furthermore, harm reduction programs targeting high-risk populations, such as needle and syringe exchange programs, have been instrumental in preventing transmission among intravenous drug users.[24] Moreover, the introduction of direct-acting antivirals (DAAs) for HCV has revolutionized treatment, achieving cure rates over 95% across all HCV genotypes.[25] Improved infection control practices in healthcare settings have also minimized iatrogenic transmissions, underscoring the effectiveness of a comprehensive approach to combating these viral infections. The World Health Organization (WHO) estimates that there are 1.5 million new HBV and HCV infections annually.[26] Acute hepatitis forms, such as hepatitis A (HAV) and hepatitis E (HEV), remain prevalent in developing countries, with approximately 10 million and 20 million new infections each year, respectively.[27,28] Socioeconomic factors exacerbate disease progression, as evidenced by the rising global incidence of cirrhosis from 20.7 to 23.4 per 100,000 people between 2000 and 2015.[9] The global incidence of cirrhosis, the end-stage of various chronic liver conditions, increased from 20.7 per 100,000 people in 2000 to 23.4 per 100,000 in 2015.[9] Moreover, the incidence of liver cancer continues to escalate, with around 20 million new cases reported globally in 2022.[29] Although improved diagnostics have enhanced early detection, they may also contribute to apparent increases in incidence. Emerging risk factors, such as environmental pollution and

hepatotoxic drug use, further complicate efforts to reduce the global burden of liver diseases.[30,31]

Acute liver disease

Acute viral hepatitis

HAV remains a significant global health concern despite strong vaccination recommendations, with an estimated 10 million new infections annually.[27] In 2019, around 159 million acute HAV infections were reported worldwide.[32] HAV prevalence varies considerably across different regions, with developing countries and low-income areas experiencing higher seroprevalence rates, particularly in sub-Saharan Africa and South Asia. In these regions, almost all children encounter HAV early in life, reducing susceptibility in adulthood.[33] Conversely, in middle-income areas such as Latin America, the Middle East, North Africa, Eastern Europe, and parts of Asia, the prevalence exhibits transitional characteristics, posing a risk to unexposed adolescents and adults.[34] High-income nations such as Western Europe, Australia, New Zealand, Canada, the U.S., Japan, and Singapore have very low HAV seroprevalence, with infections primarily confined to specific high-risk groups including travelers, men who have sex with men, drug users, the homeless, and the incarcerated.[35,36]

HEV leads to approximately 3.3 million symptomatic cases of acute hepatitis globally. The distribution of HEVs by genotype and geographic location is uneven and particularly prevalent in developing regions like South Asia, Africa, rural China, and Latin America. Here, genotypes 1 and 2 of HEV, which infect only humans and are transmitted through contaminated water sources, precipitate major outbreaks in resource-limited settings.[37,38] In recent decades, there has been a significant increase in HEV cases in Europe, with seropositivity rates ranging from 20% to 30% in countries including France, Germany, and the Netherlands.[39-41] While the overall mortality from HEV is low, specific high-risk groups, such as immunocompromised individuals, face greater health threats and higher case fatality rates.[42] Targeted monitoring and protective measures for these vulnerable groups remain crucial.

Drug-induced liver injury

Determining the precise incidence of DILI is challenging due to diagnostic complexities and

widespread underreporting. The estimates of DILI incidence fluctuate significantly, from 1 in 10,000 to 1 in 1,000,000 cases, influenced by variable factors such as diagnostic criteria, detection capabilities, population demographics, drug types, cultural factors, and reporting practices.[42,43]

In Europe, retrospective studies from the U.K. and Sweden suggest an annual incidence of DILI around 2.3-2.4 per 100,000 individuals.[44,45] France reported a higher figure from a prospective study, at 13.9 cases per 100,000, equating to over 8,000 cases annually.[46] In Iceland, prospective data revealed a rate of 19.1 cases per 100,000 people.[47] In the U.S., a Delaware-based study noted an annual incidence of 2.7 cases per 100,000 adults, with nearly 43% linked to herbal and dietary supplements.[48] A comparative analysis shows that DILI incidence is generally higher in Asian countries; a nationwide prospective study in South Korea documented an annual rate of 12 hospitalizations per 100,000 due to DILI,[49] while a retrospective study in China reported 23.8 cases per 100,000.[43] Notably, the proportion of DILI cases attributed to herbal and dietary supplements is on the rise globally, indicating an evolving trend in the epidemiology of DILI and pointing towards the need for enhanced awareness and regulation.

Chronic liver diseases

Chronic hepatitis B/D

Chronic hepatitis B (CHB) remains a significant public health challenge globally. As per the 2019 WHO data, the worldwide seroprevalence of hepatitis B surface antigen (HBsAg) is 3.8%, accounting for approximately 296 million people living with CHB.[50] The GBD study from the same year estimated CHB prevalence at around 4.1%, translating to approximately 316 million cases globally.[51] Over the three decades leading up to 2019, CHB prevalence saw a notable decrease of 31.3%.[21] This notable decline is primarily due to the global implementation of universal HBV vaccination programs, which have significantly reduced new infections among newborns and children, the most vulnerable to chronic infection.[52] Additionally, targeted public health initiatives, including enhanced maternal and perinatal healthcare services, have effectively prevented vertical transmission from mothers to infants. Increased public awareness through education campaigns, along with improved access to healthcare, has further contributed to the reduction in CHB prevalence.[53] The highest CHB prevalence rates occur in the Western

Pacific region (5.26%) and Africa (8.83%), with the disease burden in West and Central Africa accounting for 82.8% and 17.2%, respectively.[54,55] The countries most afflicted by CHB are China, India, and Nigeria, harboring 74 million, 17 million, and 15 million cases respectively.[56] Europe exhibits significant intra-regional variation in CHB prevalence, with less than 1% in Western and Northern Europe, contrasting sharply with 4-8% in Eastern Europe. Prevalence rates in the Americas are also diversified, with Mexico at 0.20% and Haiti at 13.55%.[57]

Among specific populations, approximately 2.7 million people living with human immunodeficiency virus (HIV) are co-infected with HBV, 71% of whom are in sub-Saharan Africa. Furthermore, around 0.5% (1.3 million) of injection drug users globally are HBV-positive.[58] Hepatitis delta (HDV) co-infection has been significantly impacted by vaccination efforts[59]; although an estimated 15-20 million people worldwide are infected with HDV, the exact prevalence rates are elusive due to a dearth of comprehensive studies. The most affected countries include Benin, Gabon, Mauritania, Nauru, and Mongolia, with the latter exhibiting the highest rate at 36.9% of HBsAg-positive individuals co-infected with HDV. Despite a global decline in HDV due to vaccination initiatives, persistent high prevalence in regions like Moldova and parts of Africa and Asia underscore an ongoing health burden.[60,61]

Chronic hepatitis C

In 2020, approximately 57 million people were estimated to be living with chronic HCV infection globally, exhibiting a viremia prevalence rate of 0.7%. Over 70% of these cases were concentrated in low- and middle-income countries. The highest burdens of HCV are seen in China, India, Pakistan, Russia, and the U.S., with 30 countries collectively accounting for 80% of the global HCV burden.[62]

High HCV prevalence regions include Central Asia, East Asia, and the North Africa-Middle East, each with over 3.5% of the population affected. Moderate prevalence rates (1.5-3.5%) are noted in Southeast Asia, the Andes, Central and South Latin America, Oceania, the Caribbean, Europe, and sub-Saharan Africa. Contrastingly, some Western European nations like the U.K., Denmark, France, Germany, Sweden, and Switzerland report HCV seroprevalence rates of less than 1%. The Asia-Pacific region, tropical Latin America, and North America each have relatively low prevalence rates, under 1.5%.[63] Despite a decreasing global trend in HCV

prevalence, there is a critical need for focused interventions and increased attention in high-burden countries and regions.

Chronic metabolic liver diseases-MASLD and ALD

MASLD has rapidly become the most prevalent chronic liver disease worldwide. Its global prevalence escalated from 25.3% between 1990-2006 to 38.0% between 2016-2019, heavily influenced by the rising rates of obesity and type 2 diabetes.[64] MASLD exhibits substantial regional variations; in the Americas, Latin America reports the highest prevalence at 44.4%. In the U.S., the prevalence surged from 19% in 1988-1994 to 54% in 2005-2016.[65,66] In Europe, there is variability in the prevalence of MASLD. A recent meta-analysis, which includes data updated until 2019, indicates an increase in prevalence to 30.9%.[13] Western European nations like Germany and the U.K. displaying higher rates (25%-30%), whereas Eastern countries such as Hungary and Romania have slightly lower prevalence (around 20%).[67-69] The trend is similarly upward in the Asia-Pacific region: China saw an increase from 25.4% in 2008-2010 to 32.3% in 2015-2018,[70,71] and the latest nationwide study with 5.7 million showed that the prevalence of steatosis reached 44.39% in 2022.[72] Japan experienced a rise from 20.69% in 1983 to 29.61% in 2011-2016;[73] and South Korea reports a prevalence of approximately 31.5%.[74] In Africa and the Middle East, data on MASLD incidence and prevalence in sub-Saharan Africa are largely missing, but this burden is expected to grow in the coming decades.[75] Although data are sparse for Africa and the Middle East, the prevalence in sub-Saharan Africa is anticipated to climb in the coming decades. In the Middle East and North Africa region, high obesity and diabetes rates have significantly driven up MASLD prevalence, currently estimated at 36.5%.[75,76]

Notably, lean MASLD, a subtype of MASLD characterized by a BMI < 25 kg/m², shows a significantly higher prevalence in China (approximately 20%) compared to 5-10% in Western nations.[77,78] This disparity is influenced by factors such as genetic variations (e.g., PNPLA3 gene variants), differences in body composition (higher percentage of body fat and visceral adipose tissue at lower BMI in Asians), dietary habits, environmental factors, and gut microbiome variations.[79] Despite normal BMI, lean MASLD patients in Asian populations face similar metabolic risks and liver disease progression as their obese counterparts. The high

prevalence of lean MASLD underscores the need for tailored diagnostic and screening approaches in Asian countries like China to prevent underdiagnosis, considering unique regional and ethnic characteristics.[79]

ALD remains a major global health concern. Roughly 2.4 billion people consume alcohol worldwide, contributing to about 2 million deaths from liver disease annually, half of which are related to cirrhosis from alcohol consumption. ALD ranks among the top 30 causes of death globally, with a death rate from alcohol-attributable cirrhosis of 7.2 per 100,000 people in 2010 (4.6 in females and 9.7 in males).[80] The relationship between liver-related death rates and alcohol consumption levels varies by country. European countries have historically been the largest per capita consumers of alcohol, though consumption decreased from 12.3 to 9.8 liters annually between 2005 and 2016. Conversely, alcohol consumption has been increasing in the Western Pacific, South-East Asia, and the Americas, with future growth projected until at least 2025.[81] In China, alcohol consumption has increased more rapidly over the past 30 years than in any other country.[82] Countries like the U.S. are also reporting rising rates of harmful drinking. In 2019, nations severely affected by ALD included Mongolia, Kazakhstan, El Salvador, Guatemala, Greenland, Kyrgyzstan, Poland, Rwanda, Ireland, and Brazil, all of which exhibit high alcohol consumption correlating with elevated ALD incidence and mortality rates.[21]

During the COVID-19 pandemic, social isolation and psychological stress significantly boosted alcohol consumption in certain populations in the U.S. and Europe, potentially exacerbating ALD prevalence.[83-85] Despite intensified public health efforts, MASLD and ALD continue to pose significant challenges due to their complex etiology involving genetic predisposition, environmental factors, and lifestyle choices such as diet and alcohol use.[16,86] These diseases often progress asymptomatically, complicating early detection and treatment. The COVID-19 pandemic has further exacerbated these challenges, increasing risk factors linked to lifestyle changes and stress.[87] Consequently, there is a pressing need for adaptive public health strategies that go beyond traditional interventions. This includes not only developing targeted screening and comprehensive lifestyle interventions but also exploring innovative therapeutic options and addressing social determinants of health to effectively reduce the global disease burden.

Autoimmune liver diseases

Autoimmune liver diseases, including primary biliary cholangitis (PBC), primary sclerosing cholangitis (PSC), and autoimmune hepatitis (AIH), form a significant part of the global spectrum of liver diseases, displaying notable regional and demographic variations in prevalence.

PBC predominantly affects middle-aged and older women, with a global prevalence around 14.6 per 100,000. The highest prevalence rates are seen in North America (approximately 21.8 per 100,000), Europe (14.6 per 100,000), and the Asia-Pacific region (9.8 per 100,000). Within continents, discrepancies exist; for instance, in Europe, the prevalence varies from 13.8 per 100,000 in Northern Europe to 10.3 per 100,000 in Western Europe, with scant data from Southern and Eastern Europe. In the Asia-Pacific region, Japan and South Korea report higher prevalence rates (10.4 and 8.5 per 100,000, respectively) compared to mainland China (5.8 per 100,000) and Taiwan district (3.7 per 100,000).[88]

PSC primarily affects young adult males (male-to-female ratio approximately 2:1) and shows significant regional prevalence distinctions. For instance, Northern European countries like Sweden report a high prevalence of 10.3 per 100,000, considerably greater than Southern nations like Spain (0.6 per 100,000).[89] The U.K. saw a rising prevalence from 3.2 per 100,000 in 1998 to 7.4 per 100,000 in 2014.[90] In North America, PSC prevalence (e.g., 13.6 per 100,000 in Olmsted County, Minnesota, U.S.) typically surpasses that in Europe. Conversely, Asian countries generally report lower rates, with Japan, South Korea, and Singapore exhibiting prevalence of 0.95, 0.45, and 0.15 per 100,000, respectively.[89] Notably, around 65% of PSC patients also have inflammatory bowel disease (IBD), particularly ulcerative colitis, underscoring a significant association between PSC and intestinal immune disorders.[91]

AIH has a global prevalence of 17.44 per 100,000, with regional prevalence rates at 12.99 in Asia, 19.44 in Europe, and 22.80 in the Americas per 100,000 respectively.[92] AIH incidence in Denmark rose from 1.37 per 100,000 in 1994 to 2.33 per 100,000 in 2014.[93] In the U.K., the incidence doubled from 1.27 in 1997 to 2.56 per 100,000 in 2015, with higher latitudes correlating with increased incidence.[94] Sweden saw an increase from 10.7 per 100,000 in 2003 to 17.3 per 100,000 in 2009.[95] Japan reported a substantial increase in AIH prevalence from 8.1 per 100,000 in 2004 to 23.9 per 100,000 in 2016.[96] While the incidence has remained relatively

stable in South Korea, there has been a gradual increase in prevalence from 2009 to 2013.[97] The precise etiology of AIH remains elusive, though there is speculation that environmental changes may act as triggers.[88] These diseases exemplify the complex interplay of genetic, environmental, and immunological factors that characterize autoimmune pathologies affecting the liver.

Genetic and rare liver diseases

Wilson disease (WD), an autosomal recessive disorder affecting copper metabolism, leads to significant liver and neurological damage. The estimated global prevalence of WD lies between 1:30,000 and 1:40,000 but shows notable ethnic and regional variability. In Europe, WD prevalence spans from approximately 1.2 to 2.0 per 100,000. It is comparatively higher in Asia, with China reporting a rate of 5.87 per 100,000, South Korea at 2.7 per 100,000, and Japan at 1.9 per 100,000.[98] A U.K. study highlighted a potentially higher-than-expected risk, suggesting a carrier rate for pathogenic mutations in WD at 1/7,026-indicative of an underestimation of WD prevalence within the general population.[99] The Middle East also exhibits significant prevalence, with Iran reporting 1/31,000 and Saudi Arabia at 1/15,000. In the U.S., the prevalence is around 1:30,000 to 1:40,000, but specific subpopulations like Armenians in New York City show a higher prevalence of 1/22,000. In Brazil, the prevalence aligns closely with the global average at about 1:36,000. It is notably higher in isolated regions with high rates of consanguineous marriages, such as the Canary Islands and Sardinia, with prevalence of 1/2,600 and 1/7,000, respectively.[99,100] The frequently underestimated prevalence of WD may be attributed to misdiagnosis, phenotypic diversity, inadequate sensitivity of copper metabolism tests, and low detection rates of ATP7B gene mutations.[101]

Alpha-1 antitrypsin deficiency (AATD) predominantly affects the lungs and liver and is another prevalent genetic condition with distinct genetic variations influencing its distribution. The most prevalent AATD genotype among people of European descent is Pi*ZZ, occurring at a rate of about 1/2,000 to 1/4,000.[102] North America reports a Pi*ZZ prevalence ranging from 1/3,000 to 1/5,000, with specific populations like Newfoundland experiencing higher rates, around 1/1,100.[103] Conversely, Pi*ZZ prevalence is markedly lower in Asia and Africa: Japan reports a prevalence of 1/300,000, South Korea at 1/280,000, and African Americans at 1/30,000. Notably, the prevalence of AATD in Caucasian populations is generally higher

compared to other genetic liver diseases, such as AIH, PSC, and WD.[104]

End-stage liver diseases

Cirrhosis

Cirrhosis, representing the end stage of diverse chronic liver disorders, has experienced a global surge in incidence, from 20.7 per 100,000 people in 2000 to 23.4 per 100,000 in 2015-a 13% increase.[9] The leading causes of cirrhosis include MASLD (60%), HBV (29%), HCV (9%), and ALD (2%).[105]

Regionally, HBV is most prevalent among cirrhosis patients in the Western Pacific (59%) and least prevalent in the Americas (5%). The highest proportion of cirrhosis due to HCV occurs in the Eastern Mediterranean (70%), while it is lowest in Africa and the Western Pacific (13% each). In terms of alcohol-related cirrhosis, Europe (16%-78%) and the Americas (17%-52%) report higher rates compared to Asia (0-41%). Data on MASLD as a cause of cirrhosis show that its prevalence varies, from 2% in South Korea and Brazil to 18% in Canada.[8]

In North America and Europe, MASLD is increasingly acknowledged as a primary cause of cirrhosis. For example, the prevalence of cirrhosis in the U.S. has increased between 1.5 to 2 fold over the past two decades, especially among younger populations due to prevalent obesity, diabetes, and metabolic syndrome.[103] In Germany, MASLD-related cirrhosis cases saw a fourfold increase from 2005 to 2018.[106] Similarly, in Japan, cirrhosis due to MASLD rose from 2% in 2007 to 9% in 2016[107]; South Korea also reports rising cirrhosis cases caused by MASLD, HCV, and alcohol.[108] Despite these trends, viral hepatitis remains the dominant cause of cirrhosis in the Middle East and Africa, particularly HCV.[9,109] Overall, NAFLD and ALD-related cirrhosis are becoming more common globally, although HBV and HCV infections remain the primary causes in many developing countries.

Hepatobiliary cancer

Hepatocellular carcinoma (HCC) is the most prevalent primary liver cancer globally and often develops within the context of chronic liver diseases such as cirrhosis. In 2020, the global prevalence of HCC ranged from approximately 15-30 per 100,000 with over 70% of cases in Asia.[110] Mongolia reports the highest prevalence (85.6 per 100,000) in East Asia, followed by

China (26.7 per 100,000), South Korea (21.8 per 100,000), and Japan (16.1 per 100,000).[10,111] Southeast Asia also experiences a high HCC prevalence, with numbers like Thailand (22.2 per 100,000), Vietnam (19.4 per 100,000), and Cambodia (18.3 per 100,000). In Africa, the HCC prevalence ranges from 10-20 per 100,000, with Egypt reporting the highest at 32.2 per 100,000.[112] Southern European nations like Italy (10.9 per 100,000) and Spain (8.6 per 100,000) report higher HCC rates compared to other European regions. In North America, HCC prevalence stands at about 6-8 per 100,000, while in Latin America, rates are slightly lower, ranging from 3-5 per 100,000, with Brazil and Mexico recording the highest numbers within this range. Oceania reports significantly lower HCC prevalence, as seen in Australia (3.2 per 100,000) and New Zealand (2.8 per 100,000).[113,114]

The global heterogeneity in HCC prevalence reflects the complex interplay of diverse etiological factors. In East and Southeast Asia, chronic HBV infection remains a major contributor, historically exacerbated by vaccination gaps and vertical transmission.[111,115] Conversely, Western countries typically exhibit lower HCC rates due to effective HBV vaccination programs and improved antiviral therapy access.[114,116] In Africa, particularly Egypt, high HCV prevalence is linked to elevated HCC rates, with limited healthcare access and socioeconomic challenges playing significant roles.[117] In Southern Europe, cultural practices such as traditional alcohol consumption patterns also contribute to increased HCC risk.[118] Moreover, the global rise in obesity and associated metabolic dysfunction represents an emerging HCC risk factor, particularly in Western regions.[119] Environmental factors, including aflatoxin exposure in specific African and Asian regions, further modulate HCC risk.[120] These multifaceted influences underscore the urgent need for region-specific prevention and screening strategies to effectively mitigate the global HCC burden.

Cholangiocarcinoma (CCA), though less common than HCC, has exhibited an increasing trend in the U.K. and the U.S., particularly with intrahepatic forms, while extrahepatic CCA has seen a decline.[121,122] The U.S. recorded an increase in intrahepatic CCA incidence from 0.44 per 100,000 in 1973 to 1.18 per 100,000 in 2012.[123] This rise is largely attributed to the increasing prevalence of obesity and metabolic syndrome in Western countries.[124] In contrast, CCA incidence in Southeast Asian countries like Thailand is much higher than HCC, reaching 14.6 per 100,000.[125] Factors such as liver fluke infections prevalent in Southeast Asia

significantly contribute to the high regional CCA incidence. Notably, while CCA primarily affects middle-aged and older men, the rising incidence among women and younger individuals calls for further research into its evolving epidemiology.[126]

Clinical and pathological features of liver diseases

Clinical features

Liver diseases manifest a broad spectrum of symptoms, ranging from early nonspecific signs to advanced multisystem complications (Fig. 2A). Typically, at their onset, conditions such as acute viral hepatitis, mild DILI, early-stage chronic viral hepatitis, MASLD, and initial-phase ALD manifest with mild, nonspecific symptoms.[127,128] Patients may experience minor fatigue, upper right abdominal discomfort, and a decreased appetite. Those with acute viral hepatitis might exhibit transient fever, nausea, and slight jaundice. MASLD patients often present with features of metabolic syndrome such as obesity, dyslipidemia, and hypertension.[129,130] Early ALD may manifest as indigestion and abdominal discomfort. Mild DILI might lead to slight elevations in transaminases without prominent symptoms.[131] Notably, many patients with early-stage liver disease are asymptomatic, being discovered incidentally during routine examinations or investigations for other reasons.

As liver diseases progress to the intermediate stage, symptoms previously mild or intermittent become more pronounced across various conditions. Patients with chronic viral hepatitis often experience ongoing fatigue, intermittent jaundice, and increasingly severe right upper quadrant pain, often accompanied by general malaise and mild hepatomegaly.[35] In cases of chronic HCV, approximately 70% of patients may develop systemic complications such as mixed cryoglobulinemia and cardiovascular issues, underscoring the extensive impact of the disease.[132] Concurrently, immune-mediated liver diseases demonstrate their unique progression patterns during this stage. AIH frequently manifests with nonspecific symptoms like fatigue in 85-95% of patients, often accompanied by symptoms like jaundice (67-85%) and abdominal pain (50-70%). Furthermore, 25-40% of patients display extrahepatic manifestations such as arthralgia and skin rashes, and up to 50% may suffer from concurrent autoimmune disorders such as thyroiditis.[133] PBC exhibits persistent pruritus and fatigue in 65-85% of patients, with 50-60% initially asymptomatic, typically identified during routine liver function tests.[134] It is

often associated with other autoimmune conditions such as Sjögren's syndrome (25%) and thyroid disorders (20%).[135] PSC often exhibits pruritus and jaundice; typical symptoms include fatigue (75-80%) and right upper quadrant pain (20-40%), strongly linked to inflammatory bowel disease, especially ulcerative colitis in 60-80% of cases, and an elevated lifetime risk of cholangiocarcinoma (10-15%) and colorectal cancer.[136-138] In MASLD and ALD, signs of liver function abnormalities and mild coagulation disorders emerge, indicating progressive liver impairment.[139,140]

In the severe stages of liver diseases, patients often develop more serious and complex complications. Individuals with advanced chronic liver disease and compensated cirrhosis typically exhibit signs of portal hypertension, such as ascites, splenomegaly, and palmar erythema. These signs reflect significant changes in liver structure and function and alterations in portal systemic hemodynamics. Patients with Wilson disease often begin to exhibit significant neuropsychiatric symptoms, including movement disorders and cognitive decline at this stage, likely due to disturbances in copper metabolism affecting the central nervous system.[141] Patients with AATD might concurrently suffer from respiratory symptoms such as difficulty breathing.[142] Additionally, patients with late-stage liver disease commonly develop coagulopathies and thrombocytopenia, increasing the risk of bleeding. Early hepatic encephalopathy typically presents as mild cognitive impairment and disturbances in the sleep-wake cycle, early indicators of severe liver function compromise affecting the neurological system.[143]

The end stages of liver disease, including decompensated cirrhosis and hepatobiliary malignancies, are characterized by life-threatening complications. Patients often experience esophageal and gastric variceal bleeding, refractory ascites, and severe hepatic encephalopathy. These manifestations represent the terminal expressions of hepatic synthetic failure, altered hemodynamics, and cerebral dysfunction resulting from liver failure. Patients with HCC typically exhibit weight loss, abdominal masses, and cachexia; meanwhile, CCA patients might present as painless progressive jaundice and abdominal pain. Additionally, patients with end-stage liver disease often develop multisystem complications, including systemic coagulopathy, hepatorenal syndrome, and hepatopulmonary syndrome.[144,145]

In summary, the progression of liver disease constitutes a complex, multistage process that

involves multiple systems and organs. Ranging from mild early symptoms to life-threatening complications in the end stages, the clinical manifestations at each stage illustrate the extent of liver damage and its systemic impact. Significantly, various types of liver diseases can progress at differing speeds and exhibit distinct clinical manifestations. This understanding is crucial for early detection of liver diseases, the assessment of disease severity, and accurate prognostication.

Pathological features

Liver histology, crucial for diagnosing various diseases, plays a pivotal role in understanding and recognizing hepatic conditions (Fig. 2B).

In the initial stages of liver disease, pathological alterations are generally mild. Acute viral hepatitis typically features mononuclear cell and lymphocyte infiltration in hepatic lobules and portal areas.[146] DILI may be indicated by slight hepatocellular swelling and minimal inflammatory cell infiltration.[147] MASLD and ALD initially present with steatosis without significant inflammation. These changes are typically reversible, with timely intervention preventing disease progression.[19]

As the disease progresses to moderate stages, pathological changes become increasingly pronounced. Chronic viral hepatitis is characterized by persistent inflammation, interface hepatitis, and varying degrees of fibrosis. In advanced MASLD and ALD, worsening steatosis is accompanied by significant inflammatory cell infiltration and hepatocyte ballooning.[148] PBC is distinguished by chronic non-suppurative inflammation and interlobular bile duct destruction, while PSC presents with concentric periductal fibrosis, the classic "onion-skin" appearance.[149,150] AIH is characterized by interface hepatitis with prominent plasma cell infiltration. These pathological changes reflect disease progression and potentially indicate more severe liver dysfunction.

In the severe stage of liver disease, pathological alterations become more pronounced. Advanced chronic liver disease presents with significant bridging fibrosis and early nodule formation. Wilson disease is characterized by hepatocellular copper accumulation and the presence of Mallory bodies.[151] AATD manifests as PAS-positive, diastase-resistant globules in periportal hepatocytes. Early cirrhosis, characterized by fibrous septa formation and lobular

architecture distortion, begins to manifest. These changes reflect severe liver structural and functional impairment, often indicating irreversible liver damage.[152,153]

End-stage liver disease presents the most dramatic pathological features. Liver cirrhosis is characterized by the replacement of normal liver architecture with regenerative nodules surrounded by fibrous septa, leading to severe disruption of liver structure and function. HCC shows tumor cells with varying degrees of differentiation, often with pseudoglandular structures and vascular invasion. CCA presents as adenocarcinoma with varying differentiation and prominent desmoplastic stromal reaction. Moreover, extensive hepatocellular necrosis, bile duct proliferation, and cellular atypia are observed. These end-stage changes typically signify severe liver failure, reflecting the terminal stage of the disease.[150,154]

While liver biopsy remains the gold standard for assessing liver pathology, its invasiveness and potential complications necessitate the development of non-invasive diagnostic methods. Advances in serological markers and imaging technologies strive to maintain diagnostic precision while reducing patient discomfort and risk, providing efficient alternatives to traditional histological examination.[155]

Etiology

Various factors can lead to liver injury, including viral and parasitic infections, metabolic disorders, toxic exposures (such as liver-damaging drugs), and genetic predispositions. In this section, we provide a comprehensive summary of the common causes of liver diseases.

Infection

Acute and chronic liver diseases can be caused by various viruses. HBV, an enveloped DNA virus, infects hepatocytes through bodily fluids, leading to potential CHB, cirrhosis, and HCC due to the persistence of covalently closed circular DNA (cccDNA) in hepatocytes.[156] HCV can present as acute hepatitis and lead to chronic hepatitis and cirrhosis through interactions with hepatocytes via the envelope glycoprotein E2 and immune evasion mechanisms involving the core protein.[23] HDV often co-infects with HBV, resulting in more severe liver diseases and increased mortality.[157] HAV and HEV, transmitted feco-orally, cause self-limiting diseases, with specific mechanism interactions like gangliosides for HAV and immune response dysregulation

for HEV impacting infection outcomes.[35,158] Overall, viral infection serves as a common cause of various liver diseases.

Parasitic infections such as *Schistosomiasis* and *Echinococcosis* primarily occur in rural areas, leading to severe liver complications like fibrosis and portal hypertension via mechanisms like persistent immune response to parasite eggs or direct tissue infiltration by the parasite.[159-161] Amebic liver abscesses are another consequence, originating from amoebas breaching intestinal barriers and leading to hepatic necrosis.[162]

Metabolic stress

MASLD represents the hepatic component of a multisystem disorder and is closely linked to the global rise in obesity, type 2 diabetes mellitus (T2DM), and metabolic dysfunction.[163,164] The increasing prevalence of obesity worldwide has significantly heightened the risk of MASLD development. Studies indicate that individuals with a higher body mass index are more prone to MASLD.[165,166] A recent meta-analysis reported that the prevalence of T2DM among radiologically and histologically defined metabolic dysfunction-associated steatohepatitis (MASH) patients was 22.51% and 43.63%, respectively, underlining the strong association between T2DM and MASLD.[75] Moreover, various manifestations of metabolic dysfunction, including insulin resistance, bile acid metabolism disorders, gut microbiota imbalances, and hyperuricemia, contribute to the pathogenesis of MASLD.[167-169] Patients with early MASLD exhibit hepatic steatosis and steatohepatitis, with some progressing to cirrhosis and HCC.[170] MASLD has emerged as a predominant chronic liver disease with the escalating prevalence of metabolic dysfunction.[171]

Toxic exposure

In Europe and the U.S., nonsteroidal anti-inflammatory drugs (NSAIDs), anti-infective drugs (e.g., amoxicillin-clavulanate potassium), and herbal and dietary supplements are the most common causes of DILI.[43,172] However, liver injuries induced by traditional Chinese medicine, anti-tuberculosis drugs, and other anti-infective medications are more prevalent in Asia.[43] The utilization of anti-cancer drugs and immunomodulators has also been linked to instances of drug-induced liver damage.[173] Drug risk factors such as dose and metabolism increase the risk

of liver injury with certain medications.[174] Moreover, patient genetic predisposition may be another important determinant.[175] Notably, DILI is becoming the main cause of ALF worldwide with increasing proportion.[175] Chronic alcohol consumption leads to ALD, progressing through stages from fatty liver to cirrhosis and HCC, influenced by dosage and individual factors like gender and concurrent conditions like obesity or viral infection.[19,176-180] Certain chemical substances in industrial production are hepatotropic poisons, which cause a susceptible period in the population. For example, exposure to chloroform and phosphorus has been associated with hepatic histological changes leading to toxic hepatitis.[181,182] The use of the hepatotoxic substance carbon tetrachloride (CCl_4) has become a standard method to induce murine liver fibrosis.[183] Moreover, individuals occupationally exposed to chemicals like vinyl chloride and per- and polyfluoroalkyl substances are more sensitive to MASH.[184,185] Of note, several fungi species could also produce mycotoxins with high hepatotoxicity, leading to necrosis of hepatocytes with various liver diseases.[186]

Genetic factors

Hereditary metabolic liver diseases could cause metabolic abnormalities due to the interaction between host and environmental based on genetic defects, mainly including hereditary hemochromatosis, WD, and ATTD.[187] There are 4 types of hereditary hemochromatosis based on gene mutations. Type 1 is a classic hereditary hemochromatosis, known as HFE-associated hemochromatosis. More than 80% of patients have a C282Y mutation or a C282Y/H63D complex heterozygous mutation.[188,189] These patients show hepcidin and transferrin deficiency, which impairs the transport of iron from intracellular storage sites to plasma, resulting in iron deposition in the liver.[190,191] WD results from mutations in the ATP7B gene, causing dysfunctional copper transport and accumulation.[141] Excessive copper accumulation triggers a reactive oxygen species (ROS) reaction followed by hepatic inflammation and cirrhosis.[192] ATTD is an autosomal codominant inheritance with the mutation of the SERPINA1 gene, which encoded alpha-1 antitrypsin. Abnormal conformation of this protein is detained by the rough endoplasmic reticulum, causing cellular stress, and liver disease.[193,194]

Multi-level regulatory mechanisms

Liver diseases encompass a spectrum ranging from acute and chronic liver injuries to end-stage liver diseases, each characterized with distinct pathogenesis. This section outlines the multifaceted mechanisms underlying the initiation and progression of liver diseases, spanning from molecular and cellular levels to organ interactions.

Molecular mechanisms-RIG-1/MAVs signaling

Retinoic acid-inducible gene I (RIG-1) serves as a critical RNA sensor that activates the type I interferon (IFN) response crucial for antiviral defense. RIG-1 is expressed in most cell types and is primarily localized in the cytoplasm.[195] Upon RNA recognition, RIG-1 undergoes conformational changes and translocates to mitochondria, where it interacts with its adaptor protein, mitochondrial antiviral-signaling protein (MAVS). MAVS then transmits signals from RIG-1 and activates downstream components such as TANK-binding kinase 1 (TBK-1) and IκB kinase-ε (IKK-ε). These kinases prompt the phosphorylation and nuclear translocation of interferon regulatory factor 3 (IRF-3) and IRF-7 as well as nuclear factor-κB (NF-κB), key transcription factors for the production of IFN and other cytokines.[196] Secreted IFN activates JAK/STAT signaling within host cells to engage IFN-stimulated genes (ISGs) expression, which perform essential functions in antiviral defense and the immune response (Fig. 3).[197]

HCV is a hepatotropic virus associated with liver inflammation, fibrosis, and HCC. The RIG-1 signaling has been demonstrated to play a vital role in HCV sensing and elimination.[198] Specifically, the HCV RNA genome binds to host RIG-1 to induce IFN production in hepatocytes via RIG-1/MAVS signaling.[199] This course leads to the production of ISGs like ISG15, protein kinase R (PKR), myxovirus resistance 1 (MxA), and 2′-5′-oligoadenylate synthetase (OAS), which are known for hindering cell cycle progression and the replication of HCV by impeding viral transcription and translation, initiating viral RNA cleavage, and modifying viral protein functions.[200-203] However, Li *et al.* demonstrated that HCV nonstructural protein 3-4A (NS3-4A) protease cleaves MAVS protein and diminishes MAVS/IRF-3-dependent IFN and ISGs production,[204] whereas NS3/4A inhibitors restore MAVS proteolysis and the IFN-dependent antiviral response.[205] These data may in part explain the scarcity of endogenous IFN in some HCV-infected individuals and the persistent HCV infection, as well as the importance of RIG-1/MAVS signaling in HCV elimination.

Besides HCV, the HBV DNA is involved in RIG-1/MAVS signaling activation. Pregenomic RNA of HBV is revealed to bind with RIG-1 protein, which induces IFN and ISGs production to prevent HBV infection.[206] Moreover, RIG-1 counteracts the interaction of HBV polymerase, thus suppressing viral replication and production.[207] HBV covalently closed circular DNA (cccDNA), the transcriptional template for viral RNA, is indispensable for HBV persistence and chronic hepatitis. Intriguingly, Lee *et al.* demonstrated that RIG-1/IRF-3 signaling blocks cccDNA formation and amplification in hepatocytes.[208] These findings underscore the multifunctional roles of RIG-1/MAVS signaling in anti-HBV immune response. However, HBV X protein (HBx) is shown to deactivate IFN production by deubiquitinating RIG-1 and its downstream effectors IKK-ε and IRF-3.[209] Evidence also suggests that the activation of RIG-1/MAS signaling by other viruses such as HAV, HDV and HEV combats viral replication.[210-212] In summary, RIG-1/MAVS signaling exerts pivotal roles in limiting virus replication and enhancing innate immune responses, thereby attenuating hepatic viral diseases.

Molecular mechanisms-cGAS/STING signaling

The cGAS/STING signaling pathway plays a crucial role in immune defense by detecting cytoplasmic DNA. The enzyme cyclic GMP-AMP (cGAMP) synthase (cGAS) is activated upon exposure to cytoplasmic DNA from pathogens or damaged host cells. cGAS synthesizes the second messenger cGAMP, which then binds to and activates the stimulator of interferon genes (STING). STING, localized in the ER, initiates downstream signaling to produce interferons (IFNs) and other cytokines, thereby promoting a potent innate immune response[213,214]

In the context of liver disease, cGAS/STING signaling has been implicated in the progression of conditions associated with viral infection and sterile inflammation (Fig. 3). Research indicates that HBV can evade immune surveillance by suppressing DNA sensor pathways, including the cGAS pathway, resulting in reduced expression of cGAS and its effectors during chronic HBV infection. Despite this viral evasion, activation of cGAS signaling can inhibit HBV replication. This is achieved by blocking the amplification of HBV cccDNA and reducing HBV RNA synthesis through the production of STING-mediated cytokines and ISGs, both *in vivo* and *in vitro*.[215-217] Additionally, cGAS/STING signaling has been shown to restrict HCV

replication in hepatocytes and is an important component in the immune response against this virus, as observed in studies involving STING knockdown models.[218]

The pathway is also involved in the progression of sterile inflammatory liver diseases. The release of endogenous DNA, such as mitochondrial DNA (mtDNA), during cellular damage triggers cGAS-STING signaling. This activation leads to the production of inflammatory cytokines. In cases of liver injury induced by substances such as acetaminophen and thioacetamide, hepatocyte-derived mtDNA activates cGAS-STING signaling in macrophages. This promotes an inflammatory phenotype switch in these cells, which exacerbates hepatocyte injury by promoting ferroptosis, a form of programmed cell death associated with iron.[219-221] Additionally, mtDNA-induced cGAS-STING signaling has also been reported in mice with ALD [222] and MASLD,[223] whereas overexpression of RING finger protein 13 (RNF-13) in hepatocytes attenuates liver steatosis, inflammation and fibrosis by degrading the STING protein in a mouse MASLD model.[224] Interestingly, the STING-NF-κB pathway in macrophages leads to metaflammation in lean MASLD mouse, which promotes lipolysis in the adipose tissue and subsequently contributes to liver lipid deposition and injury.[225] The evidence shows the detrimental role of cGAS/STING signaling in the regulation of sterile liver inflammation.

Molecular mechanisms-AMPK signaling

AMP-activated protein kinase (AMPK) is a highly conserved heterotrimeric protein consisting of a catalytic α subunit and regulatory β and γ subunits. This central eukaryotic energy sensor facilitates the maintenance of physiological cellular processes.[226] Upon exposure to excess energy, liver kinase B1 (LKB-1) and Ca^{2+}/CaM-dependent protein kinase kinase β (CAMKK-β), phosphorylate the Thr172 residue on the AMPK α subunit.[227] Then AMPK phosphorylates and stimulates multiple downstream substrates to regulate lipid and glucose metabolism, as well as mitochondrial function.[228] Herein, we propose the primary mechanisms by which AMPK affects liver injury, especially in MASLD and ALD (Fig. 3).

AMPK inhibits lipid synthesis by deactivating acetyl-CoA carboxylases (ACC-1 and ACC-2) and HMG-CoA reductase (HMGCR), the rate-limiting enzymes in fatty acid and cholesterol synthesis, respectively.[229] There is a negative correlation between AMPK and the development

of MASLD and ALD, as shown by the reduced AMPK levels in these fatty liver samples.[230,231] Notably, activation of AMPK by its upstream kinase LKB-1, restores hepatic lipid accumulation by downregulating lipogenesis-mediated genes, such as *Srebp1c*, *Acc*, *Fas*, *Scd1*, and *Hmgcr* in a high-fat diet-induced mouse model.[232] SREBPs, key transcriptional factors for lipid and cholesterol synthesis, are verified to contribute to MASLD and MASH-associated HCC.[233,234] Recently, studies showed that maturation and activity of SREBPs are controlled by adenosine A1 receptor (A1R) and A2R, which could be targeted to relieve MASLD.[234,235] Specific agonist of A1R, 2-chloro-N6-cyclopentyladenosine (CCPA) or screened natural compound, timosaponin AIII showed promising activity in MASLD, especially MASH therapy by activating hepatic A1R.[234] Therefore, hepatic A1R is a novel target for MASLD/MASH therapy with great potential through modulating SREBPs maturation and its controlled fatty acid *de novo* synthesis. Conversely, *in vivo* and *in vitro* studies found that hepatocytes exhibit more severe triglyceride accumulation when AMPK is depleted or blocked by its inhibitor compound C, showing the diminished protective role of AMPK against liver steatosis.[236] These data indicate the pivotal role of AMPK signaling in blocking hepatic lipogenesis.

Furthermore, AMPK promotes the expression of genes related to fatty acid oxidation in an ACC-2-dependent manner. Specifically, AMPK-inhibited ACC-2 catalyzes the generation of malonyl-CoA, which inhibits the activity of carnitine palmitoyltransferase 1 (CPT-1), a rate-limiting enzyme of mitochondrial oxidation.[237] Upon exposure to ethanol and lipids, decreased AMPK activity and fatty acid oxidation flux is observed in mouse hepatocytes.[238,239] In contrast, metformin-driven AMPK activation rescues CPT-1 expression and diminishes lipid accumulation in rat livers impacted by chronic ethanol insult.[240] Likewise, CAMKK-β-induced AMPK activation promotes fatty acid oxidation and mitochondrial biogenesis, thereby attenuating hepatic steatosis in MASLD mice.[241]

AMPK is essential to maintain mitochondrial homeostasis. Upon inflammatory stimuli, decreased AMPK activity together with mitochondrial dysfunction and ROS is observed in MASH models.[242] Growing studies showed that ethanol-induced ROS could be repressed in an AMPK-dependent manner. Impaired mitochondrial structure and increased mtDNA were observed in ethanol-treated hepatocytes, and AMPK is verified to rescue mitochondrial biogenesis and function by increasing mitophagy and ROS removal in hepatocytes.[231,243] High-

fat diet-induced ROS and ER stress were inhibited by activating AMPK/NRF-2/HO-1 signaling, which attenuated hepatic lipid accumulation and inflammation.[244-246] In addition, both *in vivo* and *in vitro* experiments demonstrated that AMPK-Caspase-6 axis relieves mitochondrial function and protect against hepatocellular apoptosis in MASH, as well as ferroptosis in ALD.[247,248] Besides, AMPK plays important roles in linking metabolism to the development of liver cancer. Lower levels of AMPK are associated with poor prognosis in HCC, while activation of AMPK expression regulates metabolic reprogramming in the tumor microenvironment, improving the efficacy of tumor immunotherapy.[249,250] Collectively, the above evidence elucidates the crucial roles of AMPK in liver metabolism and inflammation, which implicates AMPK might be a potential therapeutic target in liver diseases.

Molecular mechanisms-MAPK signaling

The mitogen-activated protein kinase (MAPK) signal transduction pathway is a critical mediator that orchestrates cellular proliferation, differentiation, and death. The core module of MAPK signaling is composed by three-tiered kinase cascade proteins, namely MAPK kinase kinase (MAPKKK), MAPK kinase (MAPKK) and MAPK. MAPKs encompass extracellular signal regulated kinases (ERK-1/2), p38α/β/δ/γ MAPK, and c-Jun-N-terminal kinases (JNK-1/2/3).[251] MAPK signaling is activated in response to extracellular stimuli, including hormones, cytokines, growth factors. Active MAPK phosphorylates and activates downstream effectors, including transcription regulators that translocate to the nucleus to manipulate target gene expression.[252] In the liver, MAPK signaling plays an important role in mediating inflammation, metabolism and cell proliferation (Fig. 4).

Multiple studies have revealed the activation of MAPK signaling in hepatocytes and macrophages during acute or chronic liver injury. Fatty acids, ethanol, and acetaminophen have been implicated in the activation of p38 MAPK, JNK and ERK signaling in hepatocytes.[253-255] The signaling cascade triggers the activation of critical regulators of lipid synthesis, autophagy, and inflammation such as SREBP-1c, sequestosome-1 (SQSTM1/P62) and NF-κB; however, blocking JNK and p38 signaling by degradation of TAK-1, a member of the MAPKKK family, reverses fat accumulation, impaired autophagy flux, inflammation, and apoptosis in hepatocytes.[253] Recently, lysosomal homeostasis and autophagic flux have been recognized as

playing a beneficial role in MASLD.[256] Lysosomal dysfunction can lead to impaired autophagic flux, inducing lipid droplet accumulation in hepatocytes and further activating HSCs in a hepatic steatosis model.[257,258] In contrast, hepatic fat accumulation and liver fibrosis is alleviated when lysosomal dysfunction is restored.[259,260] Additionally, both *in vivo* and *in vitro* studies have demonstrated that the overexpression of JNK signaling and subsequent AP-1 and NF-κB cascades in hepatocytes promotes cell proliferation and migration, thus contributing to MASLD-associated liver cancer.[261] The above evidence suggests that MAPK signaling and its downstream effectors plays pivotal roles in hepatocyte survival and function.

Emerging studies suggest that MAPK signaling in hepatic macrophages acts as a key contributor to liver injury. p38 MAPK signaling in macrophages contributes to the development of nutritional steatohepatitis by promoting M1 macrophage polarization and the release of inflammatory cytokines. In contrast, macrophage p38 MAPK deficiency in mice is associated with a hepatic M2 phenotype characterized by decreased secretion of TNF-α, IL-6, and CXCL-10, which leads to reduced fat accumulation and hepatocyte apoptosis.[262] Consistently, macrophage p38 MAPK-deficient mice are more resistant to drug-induced hepatotoxicity, as evidenced by decreased cytokine production and accelerated hepatocyte regeneration.[263] Moreover, ERK signaling in macrophages is responsible for TGF-β production, thus triggering HSC activation in response to high-fat/high-cholesterol diet.[264] Interestingly, HSC activation is directly promoted by ERK signaling. Specifically, the secretory protein ANGPTL8 from fatty hepatocytes interacts with the LILRB2 receptor on HSCs and activates ERK signaling-dependent autophagy.[265] Increased autophagy flux facilitates the transdifferentiation of HSCs into a myofiblastic phenotype, ultimately contributing to liver fibrogenesis.[266] These observations implicate the importance of MAPK signaling in the evolution of liver diseases.

Molecular mechanisms-PI3K/Akt signaling

The phosphoinositide 3-kinase (PI3K)/protein kinase B (Akt) pathway represents an evolutionarily conserved signaling cascade pivotal in cellular processes such as metabolism, survival, proliferation, and cell death. The pathway consists of two core components: PI3Ks and Akts. Upon stimulation, PI3K catalyzes phosphatidylinositol 4,5-bisphosphate (PIP2) to generate phosphatidylinositol 3,4,5-trisphosphate (PIP3), which serves as a second messenger

to recruit and activate Akt. Activated Akt then phosphorylates numerous downstream substrates to initiate multiple pathways. In this section, we focus on PI3K/Akt signaling during liver diseases (Fig. 4).[267]

PI3K/Akt signaling performs bidirectional roles in response to acute liver injury and liver fibrosis.[268,269] PI3K/Akt signaling impedes liver regeneration by promoting macrophage migration and fostering an inflammatory environment after partial hepatectomy.[270] On the other hand, PI3K/Akt signaling is responsible for the production of hepatocyte growth factor (HGF), epidermal growth factor (EGF), and TGF-β, which are essential for hepatocyte proliferation and survival.[271] In the setting of liver fibrosis, PI3K/Akt signaling in macrophages contributes to profibrotic mediators secretion, thus triggering HSC activation and ECM production.[272] However, another study revealed that PI3K/Akt signaling counteracts the TGF-β/SMAD signaling-an important player in HSC activation-to balance cell survival and proliferation under chronic stimuli.[273] In addition, activated Akt induces the expression of matrix metalloproteinase (MMPs), which plays an importance role in ECM breakdown.[274]All these data demonstrate the dual roles of PI3K/Akt signaling in liver diseases. Considering the different stages of liver diseases, as well as the diverse cellular sources of PI3K/Akt signaling, these controversial results should be interpreted cautiously.

PI3K/Akt signaling is associated with the development of HCC through its regulation of tumor cell glycolysis, growth and apoptosis. HCC cells exhibiting activated PI3K/Akt signaling show increased glucose uptake and lactate production, a phenomenon known as aerobic glycolysis or the Warburg effect, facilitating long-term cancer cell survival. In contrast, suppression of PI3K/Akt signaling transitions aerobic glycolysis to oxidative phosphorylation, accompanied by restored mitochondrial function, which indicates the involvement of PI3K/Akt signaling in metabolic reprogramming during HCC progression.[275] In addition, inhibition of PI3K/Akt signaling elicits increased expression of caspase-3 and caspase-9, apoptotic markers, within HCC cells.[276] Overall, these findings suggest the excitatory role of PI3K/Akt signaling in the evolution of HCC.

Molecular mechanisms-JAK/STAT signaling

The Janus kinase /signal transducer and activator of transcription (STAT) signaling pathway is

a highly conserved pathway that performs crucial roles in cell differentiation, metabolism, growth, and immune response.[277] Once extracellular signals such as cytokines, interferons, and growth factors, bind to their respective receptors, JAK proteins and downstream STAT proteins undergo phosphorylation. Activated STAT proteins translocate to the nucleus, where they bind to DNA sequences to regulate target gene expression.[278,279] In this context, we discuss the dysregulation of the JAK/STAT signaling in liver diseases, particularly in autoimmune and viral hepatitis (Fig. 4).

Upregulated JAK/STAT signaling has been observed in patients with PBC, a chronic autoimmune liver disease.[280] Genome-wide meta-analysis suggested a correlation between JAK/STAT signaling and PBC.[281] Importantly, a JAK-1/2 inhibitor, baricitinib, has shown promising results in reducing alkaline phosphatase (ALP) levels and liver inflammation in PBC patients based on a phase II trial.[282] Mechanistically, IFN-induced JAK/STAT1 signaling triggers the amplification of hepatic $CD4^+$ T cells and $CD8^+$ T cells, the polarization of M1 macrophages and the release of cytokines in experimental autoimmune cholangitis models, eliciting a liver immune response and inflammation.[283] This finding underscores the pivotal role of JAK/STAT signaling in modulating liver autoimmunity.

In addition, JAK/STAT signaling is implicated in IFN-induced viral hepatitis. As mentioned above, secreted IFN binds to its receptor and activates ISG expression in a JAK/STAT-dependent manner.[284] Essential ISGs such as PKR and OAS, which are crucial for restricting HBV replication, are induced by JAK/STAT signaling, whereas the inhibition of this pathway leads to diminished PKR and OAS expression during HBV infection.[285] Similarly, *in vivo* and *in vitro* studies revealed that the JAK/STAT-dependent induction of ISG-12a plays a vital role in inhibiting HCV replication.[286]

In addition to ISGs, numerous mediators involved in liver inflammation and fibrosis are also the target of JAK/STAT signaling.[287] The activation of JAK/STAT signaling in hepatocytes mediates the production of IL-6, CXCL-10 and iNOS, which promotes hepatocyte apoptosis, inflammatory cell infiltration and fibrogenesis in a mouse model of MASLD.[288,289] In summary, JAK/STAT signaling has dual roles in liver diseases: stimulating innate and adaptive immunity while governing virus elimination within distinct disease contexts.

Molecular mechanisms-Wnt/β-catenin signaling

The Wnt/β-catenin signaling performs vital functions in embryonic process and organ development. Upon Wnt proteins bind to frizzled receptors (Fzd) and low-density lipoprotein receptor-related protein 5/6 (LRP-5/6) co-receptors, signals are transduced to β-catenin to trigger downstream events. Under physical conditions, the signaling is tightly regulated through degradation complex in the cytoplasm, where a multiprotein complex involving enzymes such as E3 ubiquitin ligases leads to the degradation of signaling proteins, maintaining an inactive state.[290] Dysregulation of Wnt/β-catenin signaling is currently considered as a crucial factor in oncogenesis. The accumulation and nuclear shuttling of β-catenin result in its interaction with T-cell factor/lymphoid enhancer factor (TCF/LEF) transcription factors, activating proto-oncogenes such as myelocytomatosis oncogene (c-Myc) and cyclin-D1 (CCND-1) and thus promoting cell proliferation and migration.[291] Here, we summarize the crucial roles of Wnt signaling in liver cancer (Fig. 5).

Genetic mutations involving Wnt signaling have been reported in human HCC. Approximately 8-30% of HCC patients exhibit mutations in the β-catenin gene (CTNNB-1), which prevents β-catenin degradation and facilitates its nuclear translocation.[292,293] Integrated multi-omics analyses have revealed pathologically elevated Wnt signaling in human HCC tissues.[294] Notably, the interaction between Wnt-3a and Fzd-7 in human HCC cells drives tumor proliferation and migration by activating β-catenin-dependent signaling.[295] Intriguingly, hepatocyte-specific overexpression and activation of β-catenin protein alone are insufficient to induce HCC.[296] However, the stimulation of β-catenin in conjunction with pathological stimuli could initiate and accelerate HCC progression in mice.[297] In contrast, the prevalence of tumor in the liver with *Ctnnb-1* conditional knockout is 7-fold higher than that in wild type liver, indicating that the absence of β-catenin stimulates carcinogen-induced hepatocarcinogenesis.[298] Mutation of hepatic *Ctnnb-1* drives hepatocarcinogenesis by upregulation of pro-tumorigenic cytokines.[299] It seems contradictory that the presence of the mutated β-catenin and the absence of normal β-catenin, both contribute to the development of HCC. More in-depth studies are needed to clarify the precise mechanism.

In addition to its role in HCC, Wnt/β-catenin signaling also participates in the initiation and progression of intrahepatic cholangiocarcinoma (ICC). Notably, upregulated Wnt-7b levels are

observed in human ICC tumors and mouse ICC models, with evidence suggesting that macrophages are the cellular source of Wnt-7b production *in vivo* and *in vitro*.[300] Pharmacological or genetic inhibition of Wnt-7b-Fzd7-β-catenin signaling has shown promise in mitigating tumor growth and metastasis.[301] In summary, Wnt/β-catenin signaling contributes to the oncogenic process of liver carcinoma.

Molecular mechanisms-TGF-β signaling

Transforming growth factor-β (TGF-β) is a cytokine with three isoforms (TGF-β1, TGF-β2 and TGF-β3), sharing around 80% homology in their amino acid sequences. Upon TGF-β binding, TGF-β receptor 2 (TGF-βR2) recruits and activates TGF-βR1 to synergistically mediate downstream signaling. TGF-β/TGF-βR transmits extracellular stimuli and exhibit cellular transcriptional events by two ways: canonical SMAD-dependent pathway and non-SMAD pathways.[302] TGF-β signaling is well-recognized for inducing fibrosis in multiple organs, including the liver (Fig. 5).[303,304]

Excessive TGF-β expression is documented in both acute and chronic liver diseases across various ell types.[305] In patients with diseases such as AIH and chronic hepatitis C, increased serum and hepatic levels of TGF-β are observed, correlating with disease progression.[306,307] Transcriptome analysis from MASLD model reveal macrophages, LSECs, activated HSCs, and hepatocytes as sources of TGF-β production.[308] Notably, macrophages are identified as the predominant cellular origin of TGF-β in the injured liver.[309]

HSC activation serves as a hallmark event in the initiation of liver fibrosis, with ECM deposition characterizing fibrotic progression. TGF-β, which is induced by liver injury, triggers TGFR activation in HSCs, leading to phosphorylation of downstream effectors, such as small mothers of decapentaplegic (SMAD) proteins. Activated SMAD proteins translocate into the nucleus, where they facilitate transcription of target genes by interaction with DNA-binding transcription cofactors.[310] Literature supports that TGFR/SMAD in HSCs promotes expression of α smooth muscle actin (α-SMA), collagen type I and III, which are involved in HSC activation and extracellular matrix (ECM) composition, respectively.[311,312] In addition, SMAD also triggers the expression of lysyl oxidase-like (LOXL) and tissue inhibitor of metalloproteinases (TIMP) proteins, both of which perform essential functions in ECM

deposition and stabilization.[313,314] In contrast, mice with HSC-specific inactivation of SMAD-2 have increased susceptibility to CCl_4- and DDC-induced liver fibrosis.[315]

In addition to the canonical pathway, TGF-β also contributes to liver fibrosis through the non-SMAD pathway by interplaying with MAPK signaling and PI3K signaling. Recent work has elucidated interactions between TGF-β and p38 MAPK signaling in HSCs, driving kindlin-2 expression and subsequent immune cell adhesion, which in turn promotes HSC activation.[316] In addition, TGF-β induces ADAM12 expression via the PI3K/Akt pathway in cultured human HSCs, contributing to cell adhesion and migration.[317] Taken together, these data indicate the critical roles played by both SMAD and non-SMAD pathways in TGF-β-induced HSC activation and liver fibrosis.

20240930140611103813178797525811

ORIGINALITY REPORT

12%
SIMILARITY INDEX

PRIMARY SOURCES

1	www.science.gov<br>Internet	145 words —	1%
2	www.ncbi.nlm.nih.gov<br>Internet	77 words —	1%
3	www.frontiersin.org<br>Internet	66 words —	1%
4	link.springer.com<br>Internet	63 words —	1%
5	www.mdpi.com<br>Internet	56 words —	< 1%
6	Molecular Pathology Library, 2011.<br>Crossref	44 words —	< 1%
7	worldwidescience.org<br>Internet	38 words —	< 1%
8	www.omicsdi.org<br>Internet	37 words —	< 1%
9	Zobair M. Younossi, Grace Wong, Quentin M. Anstee, Linda Henry. "The Global Burden of Liver Disease", Clinical Gastroenterology and Hepatology, 2023<br>Crossref	36 words —	< 1%

10 www.researchgate.net
Internet
35 words — < 1%

11 Sushil Sharma. "Charnolophagy in Health and Disease - With Special Reference to Nanotheranostics", CRC Press, 2021
Publications
28 words — < 1%

12 dns2.asia.edu.tw
Internet
26 words — < 1%

13 www.wjgnet.com
Internet
26 words — < 1%

14 Weichhart, T.. "The multiple facets of mTOR in immunity", Trends in Immunology, 200905
Crossref
22 words — < 1%

15 "The 21st Conference of the Asian Pacific Association for the Study of the Liver", Hepatology International, 2011
Crossref
21 words — < 1%

16 d.docksci.com
Internet
21 words — < 1%

17 www.nature.com
Internet
20 words — < 1%

18 Weize Zhu, Ying Hong, Zhaowei Tong, Xiaofang He et al. "Activation of hepatic adenosine A1 receptor ameliorates MASH via inhibiting SREBPs maturation", Cell Reports Medicine, 2024
Crossref
19 words — < 1%

19 Yo-ichi Yamashita, Katsunori Imai, Kosuke Mima, Shigeki Nakagawa, Daisuke Hashimoto, Akira
18 words — < 1%

Chikamoto, Hideo Baba. "Idiosyncratic drug-induced liver injury: A short review", Hepatology Communications, 2017
Crossref

20 api.intechopen.com
Internet
18 words — < 1%

21 www.hindawi.com
Internet
18 words — < 1%

22 Andrew M. Moon, Amit G. Singal, Elliot B. Tapper. "Contemporary Epidemiology of Chronic Liver Disease and Cirrhosis", Clinical Gastroenterology and Hepatology, 2019
Crossref
17 words — < 1%

23 Yue Liu, Jian-Ying Sheng, Chun-Fang Yang, Junjun Ding, Yun-Shen Chan. "A decade of liver organoids: Advances in disease modeling", Clinical and Molecular Hepatology, 2023
Crossref
17 words — < 1%

24 www.patentsencyclopedia.com
Internet
17 words — < 1%

25 Ioannidis, Angeliki Diotima. "Investigating the Role of Select Receptors in Radiation-Induced Cellular Plasticity Events in Glioblastoma", University of California, Los Angeles, 2024
ProQuest
16 words — < 1%

26 Juan P. Arab, Giovanni Addolorato, Philippe Mathurin, Mark R. Thursz. "“Alcohol-associated liver disease: integrated management with alcohol use disorder”", Clinical Gastroenterology and Hepatology, 2023
Crossref
16 words — < 1%

ebin.pub

27	Internet	16 words — < 1%
28	www.medrxiv.org<br>Internet	15 words — < 1%
29	Yu Huang, Di Huang, Jiefeng Weng, Shuai Zhang, Qiang Zhang, Zhenhao Mai, Weili Gu. "Effect of reversine on cell cycle, apoptosis, and activation of hepatic stellate cells", Molecular and Cellular Biochemistry, 2016<br>Crossref	14 words — < 1%
30	archive.org<br>Internet	14 words — < 1%
31	citeseerx.ist.psu.edu<br>Internet	13 words — < 1%
32	www.jci.org<br>Internet	13 words — < 1%
33	James H. Tabibian, Anatoliy I. Masyuk, Tetyana V. Masyuk, Steven P. O'Hara, Nicholas F. LaRusso. "Physiology of Cholangiocytes", Wiley, 2013<br>Crossref	12 words — < 1%
34	Kissai, Mildred Apollo. "I. Chemical Biology Tools to Modulate the Innate Immune System: Cyclic GMP-AMP Synthase Inhibitors and Phase Separation Modulators II. Structural Studies of Vimentin-Binding Small Molecules", The Scripps Research Institute, 2024<br>ProQuest	12 words — < 1%
35	Mark Thursz, Patrick S. Kamath, Philippe Mathurin, Gyongyi Szabo, Vijay H. Shah. "Alcohol-related liver disease: Areas of consensus, unmet needs and opportunities for further study", Journal of Hepatology, 2019<br>Crossref	12 words — < 1%

36	Raj Bawa, János Szebeni, Thomas J. Webster, Gerald F. Audette. "Immune Aspects of Biopharmaceuticals and Nanomedicines", Routledge, 2019<br>Publications	12 words — < 1%
37	archive.logos-science.com<br>Internet	12 words — < 1%
38	pubmed.ncbi.nlm.nih.gov<br>Internet	12 words — < 1%
39	balkancsd.net<br>Internet	11 words — < 1%
40	europub.co.uk<br>Internet	11 words — < 1%
41	www.researchandmarkets.com<br>Internet	11 words — < 1%
42	Matteo Biolatti, Valentina Dell'Oste, Sara Pautasso, Francesca Gugliesi et al. "Human Cytomegalovirus Tegument Protein pp65 (pUL83) Dampens Type I Interferon Production by Inactivating the DNA Sensor cGAS without Affecting STING", Journal of Virology, 2018<br>Crossref	10 words — < 1%
43	Roach, Crystal Michelle. "Characterizing the Ovarian Response to Xenobiotic and Environmentally Induced Metabolic Challenges", Iowa State University, 2023<br>ProQuest	10 words — < 1%
44	Xiaoqin Wu, Xiude Fan, Tatsunori Miyata, Adam Kim et al. "Recent Advances in Understanding of	10 words — < 1%

Pathogenesis of Alcohol-Associated Liver Disease", Annual Review of Pathology: Mechanisms of Disease, 2023
Crossref

45	idpjournal.biomedcentral.com<br>Internet	10 words — < 1%
46	res.mdpi.com<br>Internet	10 words — < 1%
47	tdr.lib.ntu.edu.tw<br>Internet	10 words — < 1%
48	www.spandidos-publications.com<br>Internet	10 words — < 1%
49	www.springermedizin.de<br>Internet	10 words — < 1%
50	Ambrazevičiūtė, Ugnė. "Pharmacological Modulation of Endoglin Expression in a Liver Fibrosis Model", Lithuanian University of Health Sciences (Lithuania), 2024<br>ProQuest	9 words — < 1%
51	H Fujie. "Frequent β-catenin aberration in human hepatocellular carcinoma", Hepatology Research, 2001<br>Crossref	9 words — < 1%
52	W.C. Pang, Roberta, and Ronnie T.P. Poon. "Cancer Stem Cell as a Potential Therapeutic Target in Hepatocellular Carcinoma", Current Cancer Drug Targets, 2012.<br>Crossref	9 words — < 1%
53	de Meireles, Paula Cristina Coelho Ribeiro. "Preexposure Prophylaxis for HIV Prevention Among Men Who Have Sex with Men: Understanding Eligibility	9 words — < 1%

and Early Uptake", Universidade do Porto (Portugal), 2024
ProQuest

54 idus.us.es
Internet
9 words — < 1%

55 www.tribune.net.ph
Internet
9 words — < 1%

56 "Protein Kinases and Stress Signaling in Plants", Wiley, 2020
Crossref
8 words — < 1%

57 Friedrich Marks, Ursula Klingmüller, Karin Müller-Decker. "Cellular Signal Processing - An Introduction to the Molecular Mechanisms of Signal Transduction", Garland Science, 2019
Publications
8 words — < 1%

58 Han Zhang, Qi-Dong You, Xiao-Li Xu. "Targeting Stimulator of Interferon Genes (STING): A Medicinal Chemistry Perspective", Journal of Medicinal Chemistry, 2019
Crossref
8 words — < 1%

59 J B Trepel. "FLT3 regulates β-catenin tyrosine phosphorylation, nuclear localization, and transcriptional activity in acute myeloid leukemia cells", Leukemia, 12/2007
Crossref
8 words — < 1%

60 Ja-Jen Chang, Dai-Jung Chung, Yi-Ju Lee, Bo-Han Wen, Hsing-Yu Jao, Chau-Jong Wang. " Polyphenol Extracts Inhibit Hepatic Inflammation, Oxidative Stress, and Lipogenesis in High-Fat-Diet-Treated Mice ", Journal of Agricultural and Food Chemistry, 2017
Crossref
8 words — < 1%

61	John M. Lusa. "The Network Manager's Handbook", CRC Press, 2021<br>Publications	8 words — < 1%
62	Kim, Jieun, and Youngmi Jung. "Potential Role of Thymosin Beta 4 in Liver Fibrosis", International Journal of Molecular Sciences, 2015.<br>Crossref	8 words — < 1%
63	Ningning Rong, Ruyan Yang, Ibrahim Abdel Aziz Ibrahim, Wenlong Zhang. "Cardioprotective Role of Scopoletin on Isoproterenol-Induced Myocardial Infarction in Rats", Applied Biochemistry and Biotechnology, 2022<br>Crossref	8 words — < 1%
64	assets-eu.researchsquare.com<br>Internet	8 words — < 1%
65	digitalcommons.library.umaine.edu<br>Internet	8 words — < 1%
66	mdpi-res.com<br>Internet	8 words — < 1%
67	mymedr.afpm.org.my<br>Internet	8 words — < 1%
68	openrepository.aut.ac.nz<br>Internet	8 words — < 1%
69	www.oncotarget.com<br>Internet	8 words — < 1%
70	Nicholas Harden. "Signaling pathways directing the movement and fusion of epithelial sheets: lessons from dorsal closure in Drosophila", Differentiation, 2002	7 words — < 1%

Crossref

71 Rajkumar Rajendram, Victor R. Preedy, Vinood Patel. "Stem Cells and Bone Tissue", CRC Press, 2019
Publications
7 words — < 1%

72 Yi, Lu. "Dusp4 Regulates Intestinal Inflammation and Tumorigenesis", National University of Singapore (Singapore), 2023
ProQuest
7 words — < 1%

73 Chan, Ying Kai, and Michaela U. Gack. "Viral evasion of intracellular DNA and RNA sensing", Nature Reviews Microbiology, 2016.
Crossref
6 words — < 1%

74 Daniel Q. Huang, Norah A. Terrault, Frank Tacke, Lise Lotte Gluud, Marco Arrese, Elisabetta Bugianesi, Rohit Loomba. "Global epidemiology of cirrhosis — aetiology, trends and predictions", Nature Reviews Gastroenterology & Hepatology, 2023
Crossref
6 words — < 1%

75 Kazuhiro Imai, Sam Li Fong Yau. "Quantitative Proteome Analysis - Methods and Applications", Pan Stanford, 2019
Publications
6 words — < 1%

76 Richard M. Ransohoff, Etty N. Benveniste. "Cytokines and the CNS", CRC Press, 2019
Publications
6 words — < 1%

EXCLUDE QUOTES	OFF	EXCLUDE SOURCES	OFF
EXCLUDE BIBLIOGRAPHY	ON	EXCLUDE MATCHES	OFF
